# Life-span–dependent transcriptional dynamics of the human heart

**DOI:** 10.1126/sciadv.aeg2614

**Published:** 2026-06-17

**Authors:** Hao Jia, Xiao Chen, Yuan Chang, Yifan Wang, Eric L. Lindberg, Hao Cui, Yihang Feng, Ningning Zhang, Xiulin Zhang, Mengda Xu, Dan Shan, Yixuan Sheng, Fengxiang Wei, Xiumeng Hua, Han Mo, Yuhong Hu, Xijia Shao, Han Han, Daniel Reichart, Jiangping Song

**Affiliations:** ^1^ Department of Cardiac Surgery, National Center for Cardiovascular Diseases, Chinese Academy of Medical Sciences and Peking Union Medical College Fuwai Hospital, Beijing, China.; ^2^ Beijing Key Laboratory for Xenotransplantation, Fuwai Hospital, National Center for Cardiovascular Diseases, Chinese Academy of Medical Sciences and Peking Union Medical College, Beijing, China.; ^3^ State Key Laboratory of Cardiovascular Disease, National Center for Cardiovascular Diseases, Chinese Academy of Medical Sciences and Peking Union Medical College Fuwai Hospital, Beijing, China.; ^4^ Department of Cardiology, University Hospital, LMU Munich, Munich, Germany.; ^5^ Gene Center Munich, LMU Munich, Munich, Germany.; ^6^ Interfaculty Center for Endocrine and Cardiovascular Disease Network Modelling and Clinical Transfer (ICONLMU), LMU Munich, Munich, Germany.; ^7^ Partner Site Munich Heart Alliance, DZHK (German Centre for Cardiovascular Research), Munich, Germany.; ^8^ National Clinical Research Center for Chinese Medicine Acupuncture and Moxibustion, First Teaching Hospital of Tianjin University of Traditional Chinese Medicine, Tianjin, China.; ^9^ The Genetics Laboratory, Longgang District Maternity & Child Healthcare Hospital of Shenzhen City, Longgang Maternity and Child Institute of Shantou University Medical College, Shenzhen, China.; ^10^ Shenzhen Key Laboratory of Cardiovascular Disease, Fuwai Hospital, Chinese Academy of Medical Sciences, Shenzhen, China.; ^11^ Department of Cardiac Surgery, Fuwai Yunnan Hospital, Chinese Academy of Medical Sciences, Affiliated Cardiovascular Hospital of Kunming Medical University, Kunming, China.

## Abstract

The human heart undergoes continuous transcriptional remodeling from development through aging, yet the cellular and regulatory features governing this process remain incompletely defined. Here, we generated a single-nucleus RNA sequencing atlas of 442,239 nuclei from 54 nonfailing myocardial tissues of 29 individuals spanning development, adulthood, and aging, covering left and right ventricles. Across all major cell types, we uncovered coordinated yet cell type–specific transcriptional trajectories that converge on progressive loss of gene expression homeostasis, stress responses, and inflammatory signaling over the life span. Cardiomyocytes displayed distinct age-associated transcriptional states enriched for senescence- and disease-related signatures. Regulatory network analysis identified *PRDM16* as a transcriptional regulator whose activity declined with age in cardiomyocytes. Functional perturbation of *PRDM16* in human cardiomyocyte models induced senescence, metabolic dysfunction, and stress responses, whereas its rebalancing in aged mouse hearts improved cardiac function and partially reversed aging-associated transcriptional programs. Last, leveraging life-span–resolved single-nucleus data, we constructed cardiac transcriptomic age prediction models that closely tracked chronological age in nonfailing hearts and revealed deviations consistent with accelerated aging in cardiomyopathies. Together, this study provides a comprehensive single-nucleus resource of the human heart across the life span and delineates cellular and regulatory features associated with cardiac aging.

## Teaser

A human heart cell atlas reveals how transcription changes from development to aging, identifying a key driver of senescence.

## INTRODUCTION

Human life, from early embryogenesis through maturity and into aging, depends on uninterrupted and well-orchestrated cardiac function. The heart is composed of multiple interdependent cell lineages, including cardiomyocytes (CMs), fibroblasts (FBs), endothelial cells (ECs), vascular cells, and immune cells, whose coordinated interactions enable normal contractility, relaxation, and electrical conduction (*1*, *2*). The cellular composition and function states of the lineages change dynamically across the life span.

Cardiac development is a tightly regulated and integrated process. To meet the embryo’s increasing metabolic demands, the heart is the first organ to form and initiate functional activity during the third week of embryonic development (*3*). Although morphological and transcriptional features of the early embryonic human heart have been explored (*4*), comparatively less is known about the gene expression landscape of the human fetal heart after organogenesis. Characterizing transcriptional programs and intercellular interactions during later fetal stages may provide insights into congenital heart disease and cardiac regeneration (*5*, *6*).

As the life cycle progresses into aging, the heart undergoes gradual but sustained transcriptional remodeling, which is associated with declining cardiac functionality and increased susceptibility to cardiac diseases (*7*, *8*). Aging is a major risk factor for the development of cardiac dysfunction (*9*), and cellular senescence plays an important role in cardiac response to injury and stress (*10*). Despite these associations, specific interventions that prevent or reverse cardiac aging remain limited. Identification of key genes and pathways that change across the life span may offer opportunities to preserve cellular homeostasis and mitigate age-associated cardiac decline.

Single-nucleus RNA sequencing (snRNA-seq) enables high-resolution analysis of transcriptional states across diverse cardiac cell types, including CMs that are difficult to capture using single-cell approaches. In addition, structural and functional differences between the left (LV) and right ventricles (RV) influence development trajectories and stress responses during aging (*11*, *12*). However, comprehensive single-nucleus transcriptomic analyses of the nonfailing human heart across the life span, spanning fetal development to aging and encompassing both ventricular chambers, remain limited.

In this study, we generated a large-scale snRNA-seq dataset of nonfailing human hearts covering fetal development, adulthood, and aging, with representation of both LV and RV. Using this dataset, we characterized changes in cellular composition and transcriptional features across the life span, identified regulatory features such as PR domain containing 16 (*PRDM16*) associated with CM aging, and constructed transcriptome-based models to estimate cardiac biological age. Together, this work provides a comprehensive resource for investigating human cardiac development and aging and offers a framework for quantitative assessment of cardiac transcriptional aging.

## RESULTS

### Proliferative progenitor cells are progressively lost during fetal period and depleted in adulthood

**Fig. 1.**
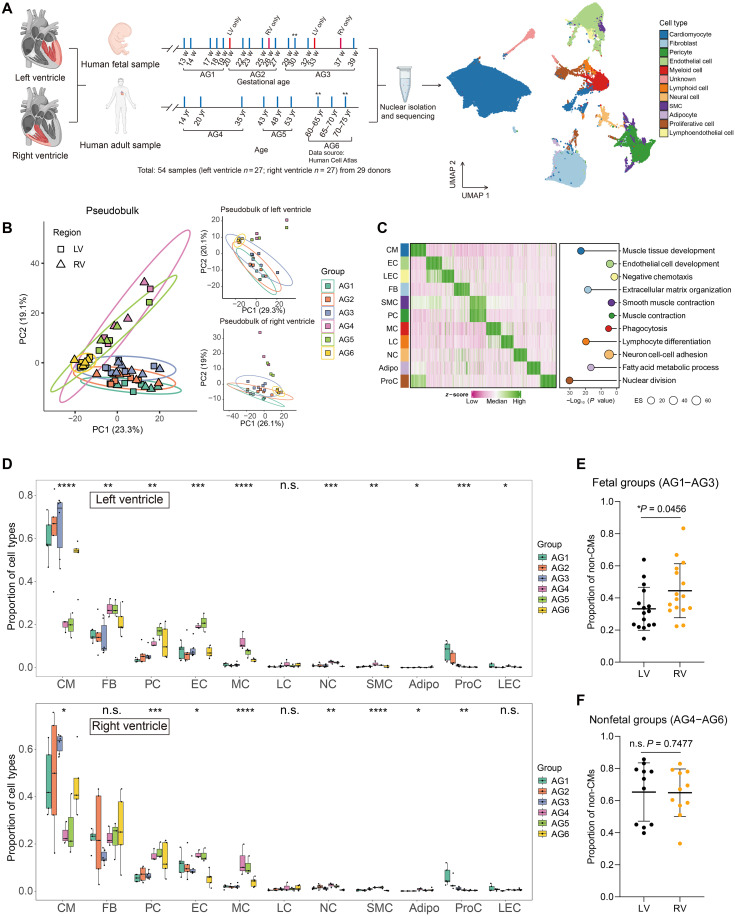
Cellular composition and abundance in the LV and RV of human nonfailing fetal and adult hearts. (**A**) Fifty-four flash-frozen and transmural LV and RV samples were obtained from 29 individuals. Only LV or RV tissues were available in four individuals as indicated by red lines. All cardiac tissues were processed using snRNA-seq. AG6 data were derived from Litviňuková *et al.* (2020). **n = 2; w, gestational weeks; yr., years. Uniform manifold approximation and projection (UMAP) plots embedding of 442,239 single-nuclei delineated 11 major cardiac cell types. Created in BioRender. Jia, H. (2026) https://BioRender.com/nl4awv1. (**B**) Principal components analysis (PCA) plots using a pseudobulk approach across all nuclei depicted similar transcriptional profiles in AG1, AG2, and AG3, as well as homogeneity within LV and RV across all age groups. (**C**) Left: Heatmap depicting the expression signatures of the top 50 marker genes. Right: Representative enriched Gene Ontology (GO) terms for each major cardiac cell type. Length of bar indicating the –log_10_ (*P* value). ES, enrichment score. (**D**) Proportion of each major cell type stratified by the age groups AG1 to AG6. Center line: median; box limits: upper and lower quartiles; whiskers: 1.5× interquartile range. Significance was tested by analysis of variance (ANOVA). LEC, lymphatic endothelial cell; Adipo, adipocyte. (**E** and **F**) Proportion of non-CMs; proliferative cell (ProC) was excluded from these proportion calculations. Data were shown as mean ± SD. Fetal groups AG1 to AG3: significance was tested with two-tailed *t* test (E); nonfetal groups AG4 to AG6: significance was tested with Mann-Whitney test (F). *P < 0.05, **P < 0.01, ***P < 0.001, and ****P <0.0001; n.s. (no significant difference) *P* > 0.05.

snRNA-seq was performed on 54 nonfailing transmural myocardial samples from 29 individuals, encompassing LV and RV (Fig. 1A and table S1) and spanning fetal development to advanced age. Samples were grouped into six age groups (AG1 to AG6), covering gestational weeks 13 to 39 and postnatal ages from 14 to 75 years: 13 to 19 weeks (AG1: LV *n* = 5, RV *n* = 5), 20 to 27 weeks (AG2: LV *n* = 5, RV *n* = 5), 29 to 39 weeks (AG3: LV *n* = 6, RV *n* = 6), 14 to 35 years (AG4: LV *n* = 3, RV *n* = 3), 43 to 53 years (AG5: LV *n* = 3, RV *n* = 3), and 60 to 75 years (AG6: LV *n* = 5, RV *n* = 5). AG6 incorporated publicly available data from Litviňuková *et al.* (*1*). Biological sex was inferred from sex chromosome–linked gene expression, revealing transient *XIST* expression in early male fetal samples (fig. S1A and table S1), consistent with reported dosage compensation dynamics (*13*).

After quality control and doublet removal, 442,239 nuclei were retained for downstream analyses (fig. S1, B and C). Pseudobulk principal components analysis (PCA) demonstrated high transcriptional similarity among fetal samples (AG1 to AG3), which were distinct from postnatal groups (AG4 to AG6), while LV and RV samples showed strong intergroup homogeneity (Fig. 1B). Unsupervised clustering identified 21 clusters, which corresponded to 11 major cardiac cell types based on canonical marker gene expression (Fig. 1, A and C; fig. S2, A to D; and table S2).

Across the life span, CM comprised a larger proportion of nuclei in fetal samples, whereas ECs, pericytes (PCs), smooth muscle cells (SMCs), myeloid cells (MCs), and neuronal cells (NCs) were more abundant in postnatal hearts (Fig. 1D; fig. S3, A and B; and table S3). During fetal development, RV samples exhibited a higher proportion of non-CMs compared with LV samples, potentially reflecting distinct contributions from the first and second heart fields (*14*, *15*). The differences diminished after birth and were no longer significant in adult and aged hearts (Fig. 1, E and F, and table S4). Life-span–association compositional changes were independently validated by multiplex immunohistochemistry (fig. S4, A to C).

Among the major cell types, a distinct proliferative cell (ProC) cluster was identified by expression of cell cycle and development markers, including *APOLD1*, *RRM2*, *MKI67*, and *TOP2A* (*16*, *17*). The proportion of ProCs declined progressively during fetal development, decreasing from 7.2% in AG1 to 1.1% in AG3, and remained below 1% across all postnatal age groups (Fig. 1D; figs. S5, A and B, and S6A; and table S5). Subclustering of ProC revealed eight transcriptionally distinct states (fig. S6B). Several ProC states expressed CM-associated genes (*MYBPC3*, *ANKRD1*, and *MYH6*), whereas others exhibited FB- or MC-associated signatures: *COL1A1*, *DCN*, *RBPJ*, and *F13A1* (fig. S6, C and D, and table S6). During fetal stages, CM-associated ProCs were predominant, while postnatal hearts were enriched for non–CM-progenitor–like states (fig. S6b and table S7).

Notably, the abundance of *MKI67*+ CM-progenitor–like cells declined markedly across fetal development (fig. S7, A and B), indicating a progressive reduction in CM proliferative capacity before birth. In postnatal hearts, residual ProCs were rare and largely associated with FB or immune lineages (*18*). These findings suggest that loss of proliferative CM potential occurs predominantly during fetal development and is largely established by birth (*19*, *20*).

### CM heterogeneity across the human life span

**Fig. 2.**
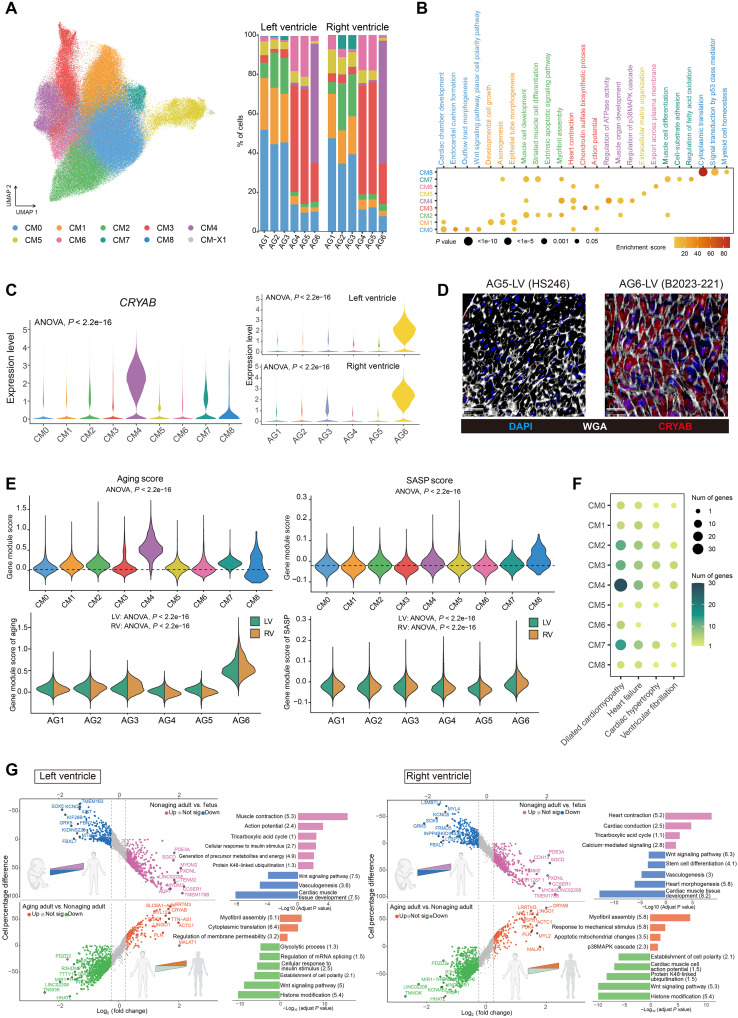
Profiling of the unique transcriptional signature of CMs across life span. (**A**) Left: UMAP embedding depicting nine CM cell states. Right: Proportion of CM cell states across all six age groups AG1 to AG6 stratified by LV and RV. (**B**) Dot plot showing the enrichment of GO biological processes in each CM cell state. ATPase, adenosine triphosphatase; p38MAPK, p38 mitogen-activated protein kinase. (**C**) Violin plots visualizing the LV and RV expression level of *CRYAB* across all CM cell states, as well as across all age groups. (**D**) Two representative images of in situ immunostaining of *CRYAB* expression in CMs showing a significantly increased *CRYAB* expression in B2023-221 (AG6; age 73) compared to HS246 (AG5; age 53). Costained with 4′,6-diamidino-2-phenylindole (DAPI) and wheat germ agglutinin (WGA). Scale bars, 50 μm. (**E**) Violin plots showing CM aging (left) and senescence-associated secretory phenotype (SASP; right) scores across all CM cell states (top) and age groups AG1 to AG6 (bottom). (**F**) Dot plot depicting the number of genes overlapping between the marker genes of each CM cell state and cardiovascular disease–associated genes. Num, number. (**G**) Volcano plots depicting the differentially expressed gene (DEG) signature comparing the fetus-dominant (AG1, AG2, and AG3: CM0, CM1, CM2, and CM7) and adolescent/adult-dominant cell states (AG4 and AG5: CM3 and CM6), as well as AG6-dominant cell states (AG6: CM4). Opaque dots represented DEGs with false discovery rate (FDR) < 0.01 and |log_2_ (fold change)| > 0.25. GO terms showed the functional enrichment of DEGs, colored according to the designations in the volcano plots. Numbers in parentheses represented the gene ratio for the GO terms. sig, significant. Created in BioRender. Jia, H. (2026) https://BioRender.com/nzzzxk9.

A total of 218,404 CM nuclei was analyzed and subdivided into 10 transcriptionally distinct CM states (Fig. 2, A and B; fig. S8, A to C; and table S8). These states exhibited pronounced age-dependent distribution, reflecting structured transition from development through adulthood and aging. Several CM states (CM0, CM1, CM2, and CM5) were enriched in fetal samples (AG1 to AG3). These states expressed genes associated with myocardial differentiation, cytoskeletal organization, and early functional maturation. CM0 showed high expression of developmental regulators, including *ERBB4* and *TBX20* (*21*, *22*). CM2 displayed increased expression of sarcomeric and actin-associated genes (*FLNC*, *NRAP*, and *ENAH*), and relative abundance of CM2 increased across fetal stages, consistent with progressive maturation of contractile function (fig. S8, C to E, and tables S8 and S9).

In contrast, CM3, CM4, and CM6 were predominantly observed in postnatal hearts (AG4 to AG6). CM3 and CM6 exhibited transcriptional characteristics of mature CMs, including genes related to calcium handling (*CACNA2D*) and contraction (*MYOM3* and *MYH6*). CM4 was strongly enriched in aged hearts and represented the dominant CM state in AG6 samples from both ventricles (LV: 61.4 ± 9.0%, RV: 64.0 ± 6.9%) (fig. S8, C to E, and table S8 and S9). In addition, Liu *et al.* (*23*) outlined contraction-related gene patterns of failing CMs, and these genes showed substantial overlap with our CM4 gene signature (table S10).

### An aging-associated CM state enriched for senescence and disease-related signatures

Given its strong enrichment in aged hearts, CM4 was further examined as a potential aging-associated CM state. CM4 exhibited increased *CRYAB* expression, a gene previously linked to CM stress and senescence (*24*, *25*). Elevated *CRYAB* expression was observed both in the CM4 state and AG6 samples and was confirmed by in situ immunostaining (Fig. 2, C and D, and fig. S9, A and B).

Consistent with these findings, CM aging and senescence-associated secretory phenotype (SASP) scores were significantly higher in CM4 and peaked in AG6 (Fig. 2E and table S11) (*26*). Neighbor-based differential abundance analysis supported a progressive transition from fetal and adult CM states toward CM4 with advancing age (fig. S10A).

Gene set enrichment analysis (GSEA) revealed that CM4 was enriched for gene sets associated with aging-related cardiovascular diseases, with dilated cardiomyopathy-associated genes showing particularly strong enrichment (Fig. 2F, fig. S10B, and table S11) (*26*). These transcriptional features suggest that CM4 represents an age-associated CM state characterized by increased cellular stress, senescence-related signatures, and overlap with disease-associated gene expression (*27*, *28*).

### Life-span transcriptional trajectories reveal progressive loss of CM homeostasis

To further characterize transcriptional dynamics underlying CM maturation and aging, we performed temporal clustering analyses across age groups. Distinct gene expression programs were identified that showed continuous decreases, continuous increases, or transient plateaus across the life span in both ventricles (fig. S10, C and D, and table S12).

Genes with progressively decreasing expression were enriched for processes related to neuronal development, muscle differentiation, and transcriptional regulation, whereas genes with increasing expression were associated with stress responses, unfolded protein response, oxidative stress, and apoptotic signaling. A third group of genes exhibited peak expression during adolescent and adult stages (AG4 and AG5), followed by decline in aging, and was enriched for pathways related to histone modification, ubiquitination, metabolic regulation, and cardiac contraction (fig. S10, E and F, and table S13). During aging, ubiquitination was dysregulated, which induced disordered protein folding and misfolded protein accumulation, leading to heart failure and arrhythmias (*29*).

On the basis of transcriptional similarity, age groups were consolidated into fetal (AG1 to AG3), adult (AG4 and AG5), and aged (AG6) stages for pairwise comparisons. The transition from fetal to adult stages was characterized by up-regulation of pathways related to energy metabolism and electrophysiological function, whereas the transition from adult to aged stages was marked by increased stress responses and disruption of protein and RNA homeostasis, including altered ubiquination, histone modification, and mRNA splicing (Fig. 2G and tables S14 and S15). Pseudotime trajectory analyses supported these observations and indicated a gradual progression toward aging-associated transcriptional states (fig. S11, A and B, and table S16).

### Identification of *PRDM16* as a transcriptional regulator of CM aging

To identify regulatory features underlying CM aging, we performed transcriptional regulatory network analysis on CMs across the life span (fig. S11C and table S17) (*30*). Transcription factors (TFs) exhibited distinct age-dependent regulatory activity patterns, with subsets showing continuous increases and decreases across age groups (fig. S11D and table S18).

**Fig. 3.**
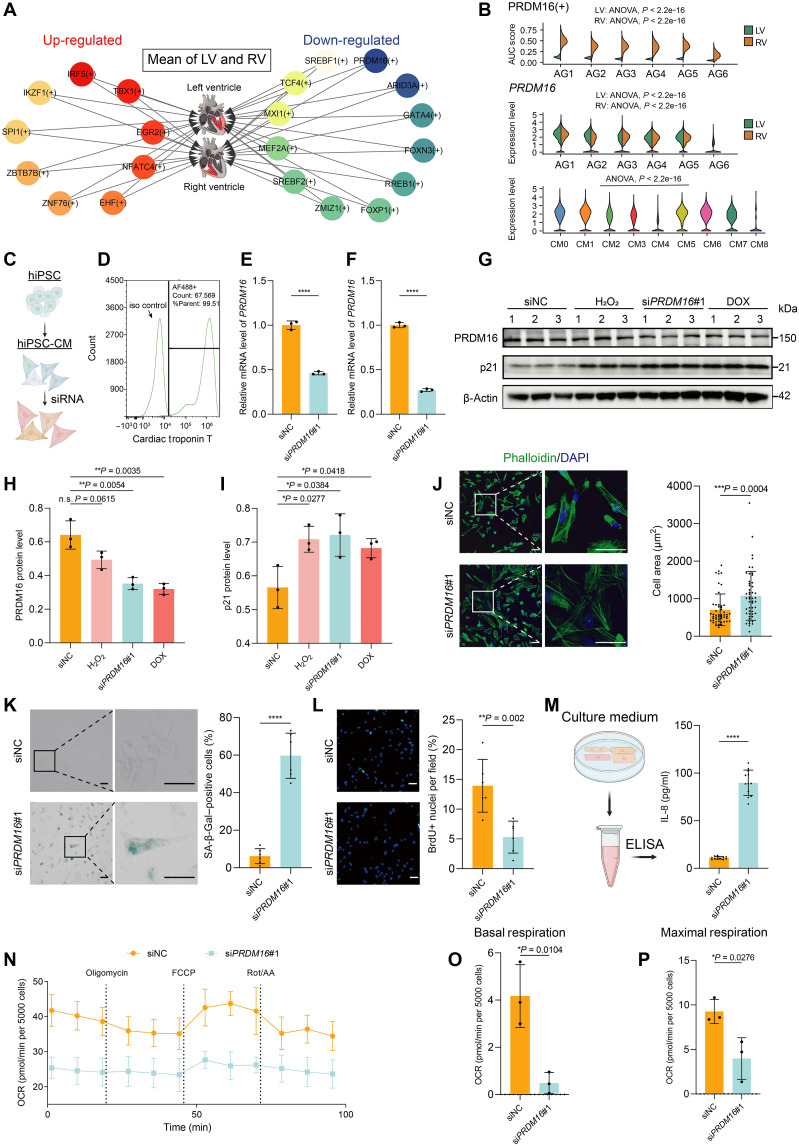
Down-regulation of *PRDM16*-induced senescence in CMs. (**A**) Network of up-regulated (left) and down-regulated (right) core regulatory TFs across life span, matching the results in fig. S11D. Color keys from light to dark indicated the mean values of |log_2_ (AG5 area under curve (AUC) score/AG6 AUC score)| of these TFs from low to high. (**B**) AUC score (top), *PRDM16* expression levels across age groups (middle), and CM cell states (bottom). (**C**) Schematic of generating *PRDM16* knockdown in human-induced pluripotent stem cell (hiPSC)–CMs. Created in BioRender. Jia, H. (2026) https://BioRender.com/c5w2f6i. (**D**) Fluorescence-activated cell sorting analysis of cTnT positive cells. Iso, isotype. (**E** and **F**) Real-time quantitative polymerase chain reaction (RT-qPCR) in hiPSC-CMs using *PRDM16*-primer-1 (E) and -primer-2 (F). *n* = 3 per group; two-tailed *t* tests. siNC, siRNA-negative control. (**G**) Western blot image of PRDM16 and p21 (β-actin control). *n* = 3 per group. DOX, doxorubicin. (**H** and **I**) Semiquantification of PRDM16 (H) and p21 (I) protein levels, matching the result of (G); two-tailed *t* tests. (**J**) Phalloidin staining of hiPSC-CMs (left). Scale bars, 50 μm. Semiquantification per cell area (right); *n* = 50 per group, Mann-Whitney test. (**K**) Senescence-associated β-galactosidase (SA-β-Gal) staining in hiPSC-CMs (left). Scale bars, 50 μm. The percentage of SA-β-Gal–positive cells (right); *n* = 6 per group; two-tailed *t* test. (**L**) 5-bromo-2′-deoxyuridine (BrdU) staining in hiPSC-CMs (left). Scale bars, 50 μm. The percentage of BrdU-positive nuclei (right); *n* = 6 per group; two-tailed *t* test. (**M**) Interleukin-8 (IL-8) concentration in cultured supernatant; *n* = 12 per group; two-tailed *t* test. (**N**) Seahorse Mito Stress Test in hiPSC-CMs. Rot/AA, rotenone/antimycin A. Created in BioRender. Jia, H. (2026) https://BioRender.com/nby7550. (**O** and **P**) Mitochondrial respiration analysis, which matched the results in (N). Basal respiration (O) and maximal respiration (P) were quantified; *n* = 3 per group; two-tailed *t* test. *P < 0.05, **P < 0.01, ***P < 0.001, ****P < 0.0001, and n.s. *P* >0.05. The results shown in (E) to (P) were from biological replicates. The data shown in (E), (F), and (H) to (P) were presented as mean ± SD.

TFs with increasing regulatory activity included *IRF5*, *EGR2*, and *NFATC4*, which have been implicated in inflammatory signaling and senescence-associated processes (*31*–*35*). In contrast, TFs with progressively decreasing activity were enriched for regulators involved in development and cell cycle programs, including *ARID3A* and *RREB1* (Fig. 3A and figs. S11E and S12A) (*36*, *37*). Members of the forkhead box (FOX) family, including *FOXP1* and *FOXN3*, also showed age-dependent loss of regulatory activity (fig. S12, B and C), consistent with observations in nonhuman primate cardiac aging (*26*).

Among all TFs analyzed, *PRDM16* exhibited the most pronounced and consistent age-dependent decline in regulatory activity in both LV and RV (Fig. 3A and fig. S11e). *PRDM16* expression levels were likewise reduced in aged hearts (AG6) (Fig. 3B). Immunofluorescence staining confirmed decreased PRDM16 protein abundance in CMs from aged samples compared with younger adult hearts (fig. S12, D and E). *PRDM16* was extensively explored in adipose tissue aging for its role in fate determination of beige adipocyte differentiation (*38*, *39*). In addition, *Prdm16* deletion in mice led to cardiac adverse remodeling, mitochondrial dysfunction, and heart failure (*40*). Together, these findings nominate *PRDM16* as a candidate transcriptional regulator associated with CM aging–related transcriptional programs.

### *PRDM16* loss functionally contributes to aging-associated CM transcriptional states

Correlation analyses demonstrated that *PRDM16* expression was negatively associated with CM aging scores (*R* = −0.6, *P* < 0.0001) and SASP scores (*R* = −0.37, *P* = 0.006) (fig. S12F and table S11). These associations, together with the consistent age-dependent decline in *PRDM16* activity, suggested that *PRDM16* may functionally contribute to the establishment or maintenance of aging-associated CM transcriptional states.

To directly assess this possibility, we knocked down *PRDM16* in human-induced pluripotent stem cell (hiPSC)–derived CMs and AC-16 cells using small interfering RNAs (siRNAs) (Fig. 3C and fig. S13A). Efficient CM differentiation was confirmed by expression of cardiac troponin T (Fig. 3D and table S19). Among tested constructs, si*PRDM16*#1 achieved effective knockdown of *PRDM16* at both mRNA and protein levels and was used for subsequent experiments (Fig. 3, E to H; fig. S13, B to F; and tables S20 to S22).

*PRDM16*-deficient CMs reproducibly developed multiple senescence-associated phenotypes, indicating that loss of *PRDM16* is sufficient to induce key features of CM aging in vitro. These phenotypes included increased expression of the cell cycle inhibitor *p21* (Fig. 3, G and I; fig. S13, E and G; and table S22), CM hypertrophy (Fig. 3J, fig. S13H, and table S23), elevated SA-β-Gal activity (Fig. 3K, fig. S13I, and table S24), reduced DNA synthesis as measured by 5-bromo-2′-deoxyuridine (BrdU) incorporation (Fig. 3L and table S25), increased secretion of the SASP factor interleukin-8 (IL-8; Fig. 3M, fig. S13J, and table S26), and impaired mitochondrial respiratory capacity (Fig. 3, N to P, and table S27). Furthermore, in a complementary model, PRDM16 expression was reduced following doxorubicin-induced senescence in hiPSC-derived CMs (Fig. 3, G to I, and table S22), further supporting the functional link between *PRDM16* loss and senescence-associated CM states (*41*).

### *PRDM16* loss partially recapitulates aging-associated CM transcriptional programs

**Fig. 4.**
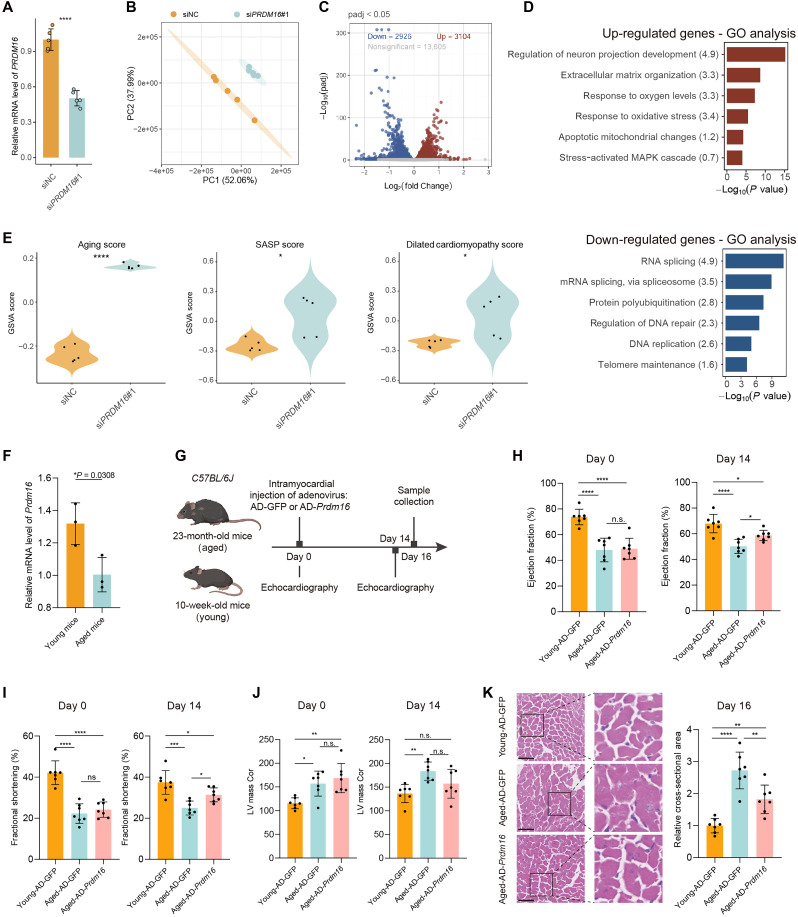
*PRDM16* regulated transcriptional profiles in CMs and cardiac aging phenotype. (**A**) RNA-seq showing silent efficacy of si*PRDM16*#1 in hiPSC-CMs; *n* = 5 per group; two-tailed *t* tests. (**B**) PCA plot of RNA-seq depicting distinct clustering of hiPSC-CM samples from siNC and si*PRDM16*#1 groups. (**C**) Volcano plot showing the distribution of DEGs between si*PRDM16*#1 and siNC groups. padj, adjusted *P*. (**D**) GO terms showing the functional enrichment of DEGs in (C). Numbers in parentheses represented the gene ratio for the GO terms. (**E**) Violin plots showing aging, SASP, and dilated cardiomyopathy (DCM) scores between siNC and si*PRDM16*#1 groups. (**F**) RT-qPCR analysis of relatively mRNA expression levels of *Prdm16* in the hearts of young mice (10-week-old) and aged mice (23-month-old) groups; *n* = 3 per group; two-tailed *t* tests. (**G**) Schematic of intramyocardial injection of adenovirus expressing GFP (control) or *Prdm16* in young (10-week-old) and aged (23-month-old) mice; *n* = 7 per group. Created in BioRender. Jia, H. (2026) https://BioRender.com/gnllkdo. (**H** to **J**) Ejection fraction (H), fractional shortening (I), and LV mass (corrected by weight) (J) of young and aged mice with intramyocardial injection of adenovirus expressing GFP or *Prdm16* at 0 and 14 days; *n* = 7 per group, by ANOVA test and Tukey tests. LV mass Cor, left ventricular mass corrected. (**K**) Hematoxylin and eosin (H&E) staining of young and aged mouse hearts (left). Scale bars, 50 μm. Semiquantification of cross-sectional area of CMs (right); *n* = 7 per group, by ANOVA test and Tukey tests. *P <0.05, **P <0.01, ***P <0.001, ****P <0.0001, and n.s. *P* > 0.05. The results shown in (A) to (K) were from biological replicates. The data shown in (A), (F), (H), (I), (J), and (K) were presented as mean ± SD.

To define transcriptional consequences of *PRDM16* loss, we performed bulk RNA-seq on hiPSC-CMs following *PRDM16* knockdown. Reduced *PRDM16* expression was confirmed in the sequencing data (Fig. 4A and table S28). PCA revealed clear separation between *PRDM16*-deficient and control CMs (Fig. 4B).

Differential expression analysis identified 3104 up-regulated and 2926 down-regulated genes following *PRDM16* knockdown (Fig. 4C and table S28). Up-regulated genes were enriched for pathways related to oxidative stress and apoptosis, whereas down-regulated genes were associated with RNA splicing and DNA homeostasis (Fig. 4D and table S28). Gene set variation analysis (GSVA) demonstrated increased aging, SASP, and dilated cardiomyopathy scores in *PRDM16*-deficient CMs (Fig. 4E and tables S11 and S28). These transcriptional changes substantially overlapped with aging-associated CM signatures identified in human hearts, indicating that *PRDM16* loss partially recapitulates the molecular features of CM aging.

### *PRDM16* gain of function attenuates age-associated CM dysfunction in vivo

To evaluate conservation and functional relevance in vivo, we analyzed mouse heart snRNA-seq data spanning embryonic to aged stages (fig. S14, A to C, and table S29). Among the seven CM states, an aging-associated CM4 state characterized by *Cryab* expression was identified in aged mouse hearts, analogous to the CM4 state observed in humans. Both *Prdm16* expression and regulatory activity were reduced in this aging-associated state (fig. S14, D to F, and table S29). After conversion to equivalent human age, the onset of cardiac aging in mice, marked by the relatively higher proportion of CM4, occurred earlier than in humans (fig. S14D). Mfuzz analysis of mouse CMs revealed that genes with progressively decreased expression over the life span were enriched in RNA homeostasis and protein ubiquitination processes (cluster A). Genes with progressively increased expression were enriched in inflammatory and stress response processes (cluster B) (fig. S14, G and H, and table S29). These findings were largely consistent with those observed in the human data.

To test whether *PRDM16* restoration could counteract age-associated cardiac changes, we overexpressed *Prdm16* in aged mouse hearts using adenovirus delivery (Fig. 4, F and G, and table S30). *Prdm16* overexpression led to substantial improvements in cardiac systolic function, as evidenced by increased ejection fraction and fractional shortening compared to control animals (Fig. 4, H and I, and table S31). In addition, CM hypertrophy was significantly attenuated in *Prdm16*-overexpressing hearts (Fig. 4, J and K, and table S31).

### *PRDM16* restoration counteracts aging-associated transcriptional features in vivo

**Fig. 5.**
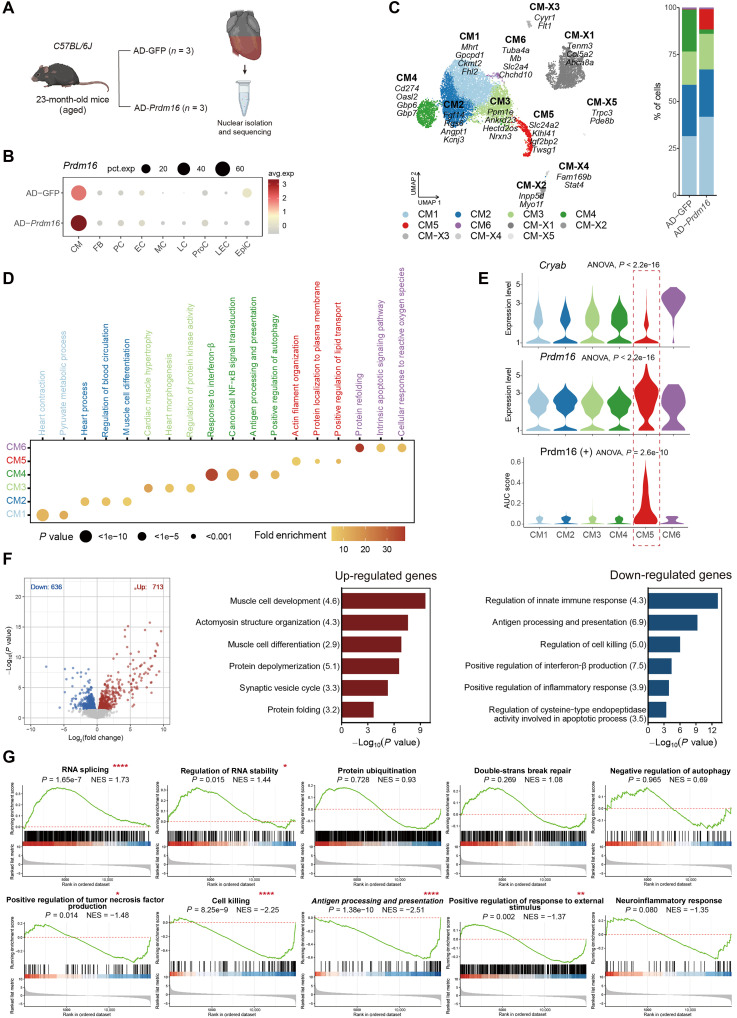
Transcriptional profiles of *Prdm16* overexpression in the aged mouse heart. (**A**) Schematic diagram of snRNA-seq, including AD-GFP group (*n* = 3) and AD-*Prdm16* group (*n* = 3). (**B**) Expression levels of *Prdm16* between the two groups and across major cell types. pct.exp, percent expressed; avg.exp, average expression. Created in BioRender. Jia, H. (2026) https://BioRender.com/jru880w. (**C**) Left: UMAP embedding depicting six CM cell states. Right: Proportion of CM cell states between the two groups. (**D**) Dot plot showing the enrichment of GO biological processes in six CM cell states. NF-κB, nuclear factor κB. (**E**) Violin plots visualizing the expression level of *Cryab* (top) and *Prdm16* (middle) and the AUC score of *Prdm16* (bottom) across six CM cell states. (**F**) Volcano plot showing the distribution of DEGs between AD-*Prdm16* and AD-GFP groups in CMs (left). GO terms showing the functional enrichment of up-regulated DEGs (middle) and down-regulated DEGs (right) in AD-*Prdm16* group. Numbers in parentheses represented the gene ratio for the GO terms. (**G**) GSEA plots showing the changes in biological processes after overexpression of *Prdm16*. NES, normalized enrichment score. *P < 0.05, **P < 0.01, ****P < 0.0001, and n.s. *P* > 0.05.

snRNA-seq was performed on hearts from aged mice expressing control [AD–green fluorescent protein (GFP)] and *Prdm16* (AD-*Prdm16*) adenovirus (Fig. 5A and fig. S15A). A total of 73,733 nuclei was analyzed, revealing comparable proportions of major cardiac cell types between groups (fig. S15, B to D, and table S32). Increased *Prdm16* expression was confirmed in CMs from AD-*Prdm16* hearts (Fig. 5B).

CMs were clustered into six distinct cell states, with one state (CM5) enriched in AD-*Prdm16* hearts (Fig. 5, C and D; fig. S15, E and F; and table S32). This state was characterized by reduced expression of *Cryab*, increased *Prdm16* expression, and elevated *Prdm16* regulatory activity (Fig. 5E). Differential expression and pathway analyses demonstrated partial reactivation of transcriptional programs that decline with age, including RNA splicing and RNA stability, alongside suppression of inflammatory and stress-associated pathways (Fig. 5, F and G, and table S32). These findings indicate that *PRDM16* gain of function counteracts multiple molecular features of CM aging in vivo.

Collectively, these results indicate that *PRDM16* is a functional regulator of CM aging. Loss of *PRDM16* is sufficient to induce senescence-associated phenotypes and aging-like transcriptional programs in human CMs, while restoration of *PRDM16* in aged hearts attenuates age-associated functional decline and partially reverses aging-related transcriptional states.

### Life-span transcriptional dynamics of non-CM populations

#### ECs exhibit age-associated inflammatory remodeling

Five EC states were identified (fig. S16A), including EC0, EC1, and EC2 as capillary ECs (*CA4*, *RGCC*, and *BTNL9*), venous ECs (*PLVAP*, *IGFBP5*, and *NR2F2*), and arterial ECs (*EFNB2*, *SEMA3G*, *GJA5*, and *DLL4*) (*1*, *42*); EC3 was identified as dividing ECs with high expression of cell cycle–related genes (*RRM2* and *MKI67*); EC4 was considered as a proinflammatory cell state with its highly expressed genes primarily enriched in antigen processing and presentation, as well as immune cell–related pathways (fig. S16, B and C, and table S33). The proliferative EC (EC3) state declined significantly across the life span (fig. S16D and table S34), consistent with reduced vasculogenic activity following development (*43*). In contrast, the proinflammatory EC state increased in abundance in aged hearts (AG6) and exhibited elevated aging and SASP scores (fig. S16, D to G, and table S34). EC4 represented a potential chronic inflammation phenotype in cardiac aging, which might be associated with a higher susceptibility to coronary artery diseases (CAD) and sudden plaque rupture in the elderly (*44*, *45*).

PCA indicated that transcriptional differences between age groups outweighed LV-RV differences (fig. S17A). Temporal analyses revealed a developmental-to-aging trajectory marked by progressive attenuation of EC developmental and adhesion-related programs and a concomitant increase in inflammatory and stress responses during aging (fig. S17, B to F, and table S35). Pairwise comparisons and pseudotime analyses further supported up-regulation of oxidative stress response and damage-related processes in aged ECs (figs. S18 and S19, A and B, and tables S36 and S37). Regulatory network analysis identified increased activity of inflammation-associated TFs, including *JUNB* (*46*), during aging, alongside reduced activity of homeobox family members (*HOXB4* and *HOXB5*), which are involved in vascular remodeling and angiogenesis (fig. S19, C to E, and table S38) (*47*). Overall, these findings indicate progressively inflammatory remodeling of the EC compartment with age.

#### FBs undergo maturation followed by inflammaging-associated transcriptional shifts

Six FB states were identified on the basis of distinct marker gene expression (fig. S20A). FB1, FB2, and FB4 states associated with development, morphogenesis, and Wnt signaling predominated during fetal stages (AG1 to AG3), whereas FB0, a mature FB state, became dominant beginning in late fetal development (AG3) and persisted into adulthood and aging (AG4 to AG6) (fig. S20, B to D, and tables S39 and S40).

In aged hearts (AG6), a distinct FB state (FB5) characterized by *APOD* expression emerged and exhibited enrichment of antigen processing, immune activation, and cytotoxic-related pathways (fig. S20, C to F, and table S40) (*48*). This state also showed elevated aging and SASP scores, consistent with a senescent FB phenotype (fig. S20G). Transcriptional analyses indicated that FB gene expression profiles matured early, with substantial overlap between late fetal and postnatal stages (fig. S21A) (*49*), followed by age-associated divergence driven primarily by inflammatory and oxidative stress pathways (figs. S21, B to F, S22, and S23, A and B; and tables S41 to S43). Regulatory network analysis revealed declining activity of stress-protective TFs, including *BACH2* (*50*), alongside increased activity of inflammation-associated TFs such as *JUN* (fig. S23, C to E, and table S44) (*46*). These changes suggest accumulation of stress and reduced adaptive capacity in FBs with advancing age.

#### Vascular SMCs and PCs converge on inflammatory phenotypes in aging

Three SMC cell states were identified, corresponding to previously described functional and proliferative SMC populations (fig. S24, A to D, and table S45) (*1*). An aging-enriched SMC state (SMC2) emerged in AG6, characterized by expression of interferon-responsive genes and immune-related pathways, including antigen processing and presentation (fig. S24, C to G, and tables S45 and S46). PCs exhibited relatively stable distributions across early and midlife stages, but in aged hearts (AG6), a shift toward PC2 state enriched for immune-related pathways (replacing PC1, which was characterized by cell differentiation) was observed (fig. S25, A to E, and tables S47 and S48).

#### Immune cell composition shifts toward proinflammatory stages with age

Lymphoid cell (LC) population comprised T and natural killer cells (LC0, LC1, LC2, LC4, and LC7), B cells (LC3), mast cells (LC5), and progenitor-like cells (LC6) (fig. S26, A and B, and table S49). B cells were more abundant during fetal development (fig. S26, B to D, and table S50), consistent with reported roles in CM proliferation and regeneration (*51*). *IL2RA*+ regulatory T cell (LC7; T_reg_ cell) was enriched in fetal hearts but declined progressively across the life span (*52*), whereas *CD8*+ T cells (LC4) increased and became the dominant lymphoid population in aged hearts (AG6) (fig. S26, B to E, and table S50) (*53*, *54*). T_reg_ cells are important effector cells in fetal growth and induced maternal-fetal immune tolerance to prevent fetal loss (*55*, *56*), which was supported by the high abundance of LC7 in fetal groups, especially in AG1.

MCs showed dynamic changes across the life span (fig. S27, A and B, table S51), with a developmental MC state (MC1) enriched for angiogenic and cytokine signaling predominating during fetal stages (AG1 to AG3), followed by emergence of inflammatory MCs in adulthood and aging (fig. S27, B and C, and tables S51 and S52) (*57*). In aged hearts (AG6), *CD14*+ monocyte–derived MCs (MC2) increased, consistent with immune remodeling in response to stress and damage (fig. S27, B to E, and tables S51 and S52) (*58*).

### Dynamic changes in intercellular communication across the human life span

To investigate age-associated alterations in intercellular communication in the human heart, we investigated ligand-receptor–based signaling interactions across all 11 major cardiac cell types using CellChat (*59*–*61*). Across the life span, both the overall number and the interaction strength of inferred cell-cell communication progressively declined from fetal development to aging (fig. S28, A and B, and tables S53 and S54). Intercellular communication contributes to the maintenance of myocardial integrity, function, and cardiac repair after injury (*62*), and its disruption might impair these processes. Our previous life cycle study on the lungs revealed similar cellular interaction results (*63*), and this theory was further supported by aging studies in the reproductive and nervous systems (*64*, *65*).

Despite the overall decline in communication strength, analysis of dominant signaling pathways revealed similarities between fetal hearts (AG1 to AG3) and aged hearts (AG6). Both stages exhibited enrichment of signaling pathways associated with stress and injury response, including thrombospondin (*66*), fibronectin1 (*67*), and amyloid-β precursor protein (*68*) signaling. In contrast, adolescent and adult hearts (AG4 and AG5) displayed a distinct signaling landscape, with dominant pathways related to growth, metabolic regulation, and vascular remodeling, including insulin-like growth factor (IGF) (*69*), ADIPONECTIN (*70*), and angiopoietin (figs. S29, A and B, and S30, A to D; and tables S53 and S54) (*71*). These patterns suggest that midlife hearts maintain a more homeostatic and growth-supportive intercellular communication network, which becomes progressively attenuated with aging. Together, these analyses indicate that cardiac intercellular communication undergoes systematic remodeling across the life span, characterized by a reduction in signaling intensity and a shift in dominant signaling pathways.

### Single-nucleus transcriptomic models predict cardiac biological age

**Fig. 6.**
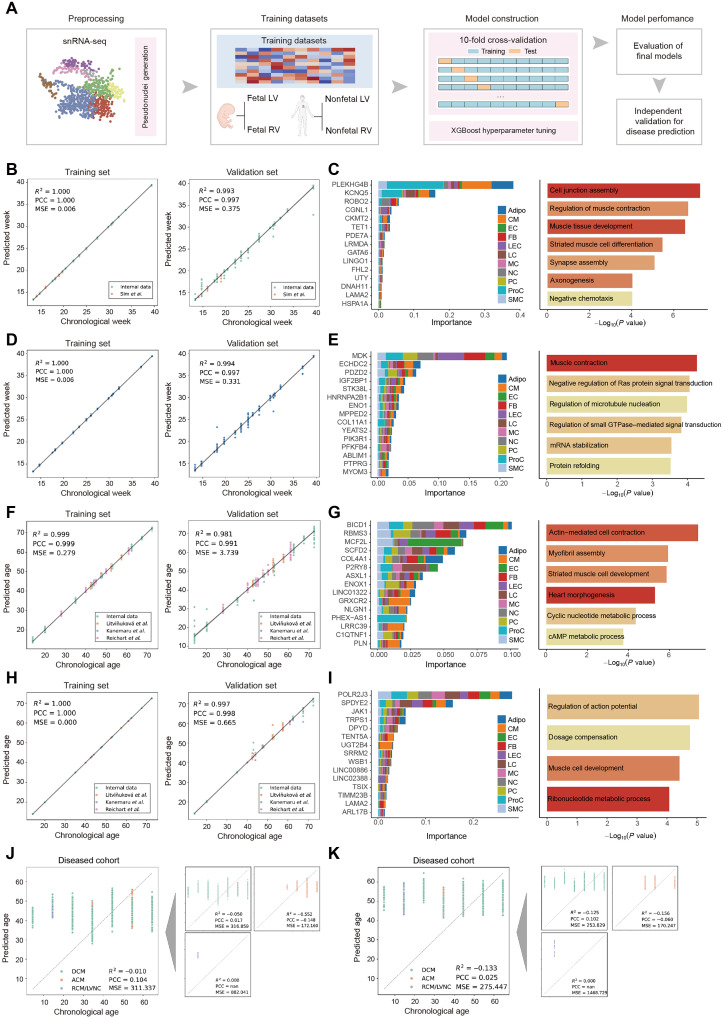
Single-nucleus heart age prediction model. (**A**) Workflow of the heart gestational week or age prediction model construction. Created in BioRender. Jia, H. (2026) https://BioRender.com/ogam4q5. (**B**) Scatter plot showing the correlation of single-nucleus predicted week with chronological week of the fetal LV in the training set (left) and validation set (right). (**C**) Bar plot showing the top 15 key genes that contributed the highest importance to the XGBoost model of the fetal LV (left). Bar plot depicting GO functional enrichment of these top key genes (right). (**D**) The training set and validation set of the fetal RV. (**E**) Key genes with the highest importance in model construction and their GO functional enrichment of the fetal RV. (**F**) The training set and validation set of the nonfetal LV. (**G**) Key genes with the highest importance in model construction and their GO functional enrichment of the nonfetal LV. cAMP, adenosine 3′,5′-monophosphate. (**H**) The training set and validation set of the nonfetal RV. (**I**) Key genes with the highest importance in model construction and their GO functional enrichment of the nonfetal RV. (**J** and **K**) Scatter plot showing the correlation of predicted age with chronological age in the diseased-LV (J) cohort using nonfetal LV model and in the diseased-RV cohort using nonfetal RV model (K). Merged scatter plot of various types of cardiomyopathies: Green dots represented DCM, blue dots represented restrictive cardiomyopathy (RCM)/left ventricular noncompaction cardiomyopathy (LVNC), and red dots represented arrhythmogenic cardiomyopathy (ACM) (left). Separated scatter plots of each cardiomyopathy type (right), with colors matching those in the left merged plot. Each dot in the scatter plots [(B), (D), (F), (H), (J), and (K)] represented a pseudobulk subset, and each single-cell dataset was divided into 60 pseudobulk subsets.

The life-span–resolved single-nucleus transcriptomic dataset revealed consistent and progressive transcriptional changes across developmental and aging stages, suggesting the feasibility of constructing quantitative models to estimate cardiac biological age. To this end, we developed transcriptome-based heart age prediction models using snRNA-seq features derived from nonfailing human hearts. Given the distinct age ranges and transcriptional characteristics of fetal and postnatal samples, separate prediction models were constructed for fetal and nonfetal hearts. In addition, models were generated independently of the LV and RV to account for chamber-specific transcriptional features (Fig. 6A).

#### Fetal cardiac age prediction models accurately reflect gestational age

The fetal LV prediction model showed strong performance in nonfailing samples, with predicted transcriptomic age showing a near-perfect correlation with chronological gestational age (Pearson correlation coefficient (PCC) = 0.997, Mean squared error (MSE) = 0.375) (Fig. 6B). Genes contributing most strongly to model performance were enriched for biological processes related to muscle contraction, CM development and differentiation, and axonogenesis (Fig. 6C and table S55), consistent with key transcriptional programs active during fetal heart maturation.

Similarly, the fetal RV prediction model accurately tracked gestational age (PCC = 0.997, MSE = 0.331) (Fig. 6D). Key genes in the RV model were enriched for pathways related to muscle contraction and mRNA and protein homeostasis (Fig. 6E and table S55), indicating chamber-specific transcriptional features during fetal development.

#### Nonfetal cardiac age prediction models capture adult and aging trajectories

In postnatal samples, both LV and RV age prediction models exhibited robust performance. The nonfetal LV model showed a strong correlation between predicted and chronological age (PCC = 0.991, MSE = 3.739), while the RV model demonstrated similarly high accuracy (PCC = 0.998, MSE = 0.665) (Fig. 6, F to I). Genes contributing to the LV model were primarily associated with myocardial contraction and development, whereas genes contributing to the RV model were enriched for pathways related to action potential generation and electrophysiological processes (Fig. 6, F to I, and table S55).

#### Cardiac aging clocks reveal transcriptional age dysregulation in cardiomyopathies

To evaluate whether the cardiac age prediction models could detect deviations from physiological aging, we applied the models to cardiomyopathy samples. In both LV and RV, predicted transcriptomic age deviated significantly from chronological age in diseased hearts (Fig. 6, J and K). These deviations were consistent with accelerated or dysregulated transcriptional aging, indicating that cardiomyopathy is associated with disruption of age-associated transcriptional homeostasis.

Together, these results demonstrate that single-nucleus transcriptomic features can be leveraged to construct robust cardiac age prediction models that accurately reflect chronological age in nonfailing hearts. Deviations from predicted age in cardiomyopathy samples highlight the sensitivity of these models to pathological remodeling and support their utility for quantifying cardiac biological age.

## DISCUSSION

In this study, we generated a comprehensive single-nucleus transcriptomic atlas of the nonfailing human heart spanning fetal development, adulthood, and aging, with representation of LV and RV. This dataset enabled systematic investigation of cardiac dynamics across the life span, including changes in cellular composition, transcriptional states, regulatory programs, and intercellular communication. By integrating human and mouse data, we further assessed the extent to which murine models recapitulate key aspects of human cardiac development and aging.

A central observation of this work is the pronounced decline of ProC populations across the life span. A subset of ProCs exhibited CM-like transcriptional signatures and was predominantly detected during fetal development, with a significant reduction in adolescent and adult hearts. In mammals, myocardial regenerative capacity is present at birth but rapidly diminished postnatally, as demonstrated in mice, opossums, and pigs (*6*, *72*, *73*). Consistent with these findings, most regenerated CMs originate from preexisting CM lineages rather than from unspecified stem cell populations (*6*, *73*). Our data indicate that the decline of ProCs—particularly those with CM fate bias—occurs largely during fetal development, suggesting that loss of regenerative potential is established before birth.

Beyond early developmental changes, we observed progressive transcriptional remodeling associated with aging. Loss of proteostasis, epigenetic alterations, transcriptional and splicing dysregulation, impaired nutrient sensing, and stem cell exhaustion emerged as prominent features of cardiac aging. These processes accumulated gradually across adulthood and were accompanied by a marked enrichment of senescent cell populations after ∼60 to 65 years of age (*74*). Aging hearts also exhibited widespread transcriptional signatures of chronic inflammation, particularly within non-CM populations (*59*). ECs, SMCs, and PCs displayed pronounced inflammaging features in late life, consistent with increased susceptibility to CAD in elderly individuals. Chronic vascular inflammation and immune cell recruitment are well-recognized contributors to atherosclerotic plaque formation (*75*, *76*), providing mechanistic context for these observations.

Within CMs, we identified *PRDM16* as a transcriptional regulator whose activity declines with age. Functional perturbation experiments demonstrated that *PRDM16* loss is sufficient to induce SASP and aging-like transcriptional programs in human CMs in vitro, while restoring *Prdm16* expression in aged mouse hearts attenuated age-associated functional decline and partially reversed aging-related transcriptional features in vivo. These findings indicate that *PRDM16* contributes to CM aging and stress responses. Notably, *PRDM16* has also been implicated in cardiac development and cardiomyopathy, including LV noncompaction and metabolic cardiomyopathy (*77*, *78*), suggesting that its cardioprotective role extends across the life span.

Analysis of intercellular communication revealed a progressive reduction in the number and strength of ligand-receptor interactions with age, reflecting attenuation of coordinated cellular signaling in the aging heart. This decline showed a linear relationship with age and was accompanied by shifts in dominant signaling pathways. While adolescent and adult hearts were enriched for growth- and metabolism-associated signaling, fetal and aged hearts shared stress- and injury-related signaling programs.

Last, leveraging life-span–resolved single-nucleus transcriptomic data, we developed cardiac aging clocks that accurately tracked chronological age in nonfailing hearts and revealed deviations consistent with accelerated transcriptional aging in cardiomyopathies (*79*, *80*). These models underscore the complexity of cardiac aging, support the value of biological age estimation beyond chronological measures, and provide a quantitative framework for summarizing cardiac transcriptional states and for assessing deviations associated with disease.

Several limitations should be noted. Sex-specific effects were not systematically analyzed and will require future studies with larger cohorts. In addition, this study focused on nonfailing hearts; integration with spatial and longitudinal datasets will be necessary for investigating region-specific and temporal aspects of cardiac aging. Nevertheless, the fetal and aging datasets presented here offer a valuable resource for identifying cardioprotective and rejuvenation-associated factors across the human life span.

In summary, this study defines cellular and transcriptional features governing human cardiac development and aging, identifies *PRDM16* as a functional contributor to CM aging, and establishes single-nucleus transcriptomic aging clocks for the human heart. Together, these findings frame cardiac aging as a coordinated, multicellular process and provide tools for studying cardiac aging in health and disease.

## MATERIALS AND METHODS

### Data reporting

No statistical methods were used to predetermine sample size. The experiments were not randomized, and investigators were not blinded to allocation during experiments and outcome assessment.

### Usage of public database

The snRNA-seq data for the AG6 group was the publicly available data from Litviňuková *et al.* (*1*). LV- and RV-derived snRNA-seq data from individuals D2, D4, D5, D6, and D7 were used. Tissues were acquired from donors after circulatory death (DCD). D2 and D7 were in the 60-to-65 age bracket, D5 was in the 65-to-70 age bracket, and D4 and D6 were in the 70-to-75 age bracket (table S1). Research ethics, tissue acquisition and processing, single nuclei isolation, and single-cell preparation methods were disclosed in the study (*1*).

### Research ethics for donor tissues

Human fetal heart tissues (donors PT-13w, PT-14w, PT51, PT49, PT66, PT45, PT21, PT90, PT19, PT42, PT25, PT102, PT7, PT12, PT18, PT85, PT96, and PT50) were processed at the Longgang District Maternity and Child Healthcare Hospital of Shenzhen City after Research Ethics Committee approval (no. LGFYYXLL-018). The tissues were obtained with the informed consent of the pregnant women and the donor families. All the fetal samples were obtained according to the Chinese legal requirements. Termination of pregnancy for fetuses older than 28 weeks of gestational age complied with Article 18 of Chapter III of the Maternal and Child Health Law of the People’s Republic of China, which states the condition under which the pregnancy can be terminated: “Fetus has serious developmental defects” and “continuation of pregnancy may endanger the life safety of the pregnant woman or seriously endanger the health of the pregnant woman due to serious diseases”(table S1). Cardiac developmental defects were not included in this study.

Nonfetal heart tissues (donors HS316, HS257, HS235, HS197, HS173, HS246, B2023-221, B2023-275, B2023-94, B2023-223, B2023-238, and B2023-176) were collected at Fuwai Hospital after Research Ethics Committee approval (no. 2013-496). The donor hearts were obtained from those initially considered for heart transplantation (HTx) but excluded because of heart size or blood type mismatch. Informed consent from donor families was acquired. We conformed to the principles outlined in the Declaration of Helsinki.

### Sample processing and preservation

Heart tissues were acquired from aborted fetus and nonfetal donors. The aborted fetus was confirmed as circulatory dead (DCD) by two senior obstetrician or neonatal intensive care unit doctors. Heart tissues from nonfetal donors were obtained after their brain death (DBD). For fetal DCD hearts, after death was declared, the chest was opened, major blood vessel was cross-clamped, and fetal hearts were obtained without cardioplegia. For nonfetal DBD donors, heart samples from these donors were obtained in accordance with the standard procedure for obtaining donor heart for HTx, the aorta was cross-clamped, cardioplegia (HTK solution, Dr. Franz Köhler Chemie) was used to arrest heart beating, and the hearts were acquired and temporarily stored in cold saline. Heart samples were dissected within five min after removal. Transmural full-thickness (containing endo-, myo- and epicardium) heart sections from the LV and RV were collected. Cardiac tissues for single nuclei isolation were snap frozen in liquid nitrogen and stored at −80°C. Formalin-fixed tissues were prepared for in situ immunostaining.

### Single nuclei isolation

Frozen cardiac tissues were used for single nuclei isolation. Samples with an RNA integrity number (RIN) value above 8 were used for single nuclei isolation and included in this study to avoid bias introduced by mRNA degradation. Heart tissues were dissected in 4°C 1× phosphate-buffered saline (PBS) buffer (Gibco, C10010500BT). The tissue fragments were submerged in 30 ml of lysis buffer [0.32 M sucrose, 5 mM CaCl_2_, 3 mM C_4_H_6_MgO_4_, 0.5 mM EGTA, 10 mM tris-HCl (pH 8.0), 2 mM EDTA, 1 mM phenylmethylsulfonyl fluoride (PMSF), 1 mM dithiothreitol (DTT), and RNA inhibitor (80 U/ml)] (*81*). Fragments were then homogenized using a Dounce tissue grinder (pestle B). The supernatant was filtered through 100- and 70-μm strainers (Corning). After centrifugation (700*g* for 10 min at 4°C), the supernatant was removed, and the pellet was resuspended using 20 ml of preservation buffer [2.1 M sucrose, 3 mM C_4_H_6_MgO_4_, 10 mM tris-HCl (pH 8.0), 1 mM PMSF, 1 mM DTT, and RNA inhibitor (80 U/ml)] (*81*). After recentrifugation (13,000 rpm for 1 hour at 4°C), the supernatant was removed, and the pellet was resuspended using 1× PBS buffer. The nuclei suspension was mixed with trypan blue (1:1), and nuclei were counted using a hemocytometer (Thermo Fisher Scientific, Countess 3).

### Preparation of nuclei for snRNA-seq

After isolation, nuclei were processed using the Chromium Controller (10x Genomics) according to the manufacturer’s protocol with Chromium Single Cell 3′ Reagent Kits (v3.1, 10x Genomics). Sequencing was performed using the NovaSeq 6000 system (Illumina) with targeted sequencing saturation of 0.7 per sample, which corresponded to an average targeted read number of 50,000 per nucleus.

### Quality control and processing of snRNA-seq data

The snRNA-seq data across 44 heart samples compiled in this study were aligned to the human genome (version refdata-gex-GRCh38-2020-A, 10x Genomics) using the Cell Ranger pipeline from 10x Genomics (version 6.1.2) to obtain raw and filtered feature expression matrices per sample. DecontX, a Bayesian method (*82*), was used to computationally estimate and remove ambient mRNA signals. The filtered count matrix was used as input, and raw count matrix was used to provide background information. Then, the background-removed expression matrices obtained from our 44 heart samples were merged along with the 10 publicly available snRNA-seq data using the Seurat package (version 4.1.1).

Cell-identifying barcodes detected with genes >300 and <6000 (300 < *n*_genes < 6,000) and with total unique molecular identifier counts >500 or <15,000 (500 < *n*_counts < 15,000) were kept. Nuclei in which more than 5% of transcripts mapped to mitochondrial genome encoded genes were also excluded. After quality control, the filtered gene expression matrix was normalized by the “NormalizeData” function in Seurat (version 4.1.1) with scale.factor set to 10,000, followed by a log transformation using the “LogNormalize” function. The top 2000 features with high cell-to-cell variation were identified using the FindVariableFeatures function and “vst” method. We used the “RunHarmony” function in the Harmony package (version 1.0) to remove batch effects among different samples after performing PCA on the highly variable genes. A shared nearest neighbor graph was built using the top 20 principal components; cells were clustered using the Louvain algorithm on the base of Harmony embeddings.

### Differentially expressed gene identification, Gene Ontology enrichment, and gene score calculation

Differential gene expression testing was performed using the presto package in R with the “wilcoxauc” function based on the normalized count matrix. Only genes expressed in at least 10% cells in a tested cluster with a maximum adjust *P* value of 0.01 and minimum |log_2_ (fold change)| of 0.25 were regarded as statistically significant differentially expressed genes (DEGs). Gene Ontology (GO) enrichment analysis was performed by the R package clusterProfiler (version 4.1.0). SASPscore and Agingscore of nuclei were calculated by the “AddModuleScore” function from Seurat (version 4.1.1). The gene sets used for the calculation of SASPscore and Agingscore were derived from previous study by Zhang *et al.* (*26*).

### Cell cycle analysis

The cell cycle phase of each nucleus was predicted by “CellCycleScoring” function in Seurat, which could assign each cell a score based on its expression of G_2_-M and S phase markers, along with predict classification of each nuclei in G_2_-M, S, or G_1_ phase. The list from Kowalczyk *et al.* (*83*) was used as a cell cycle phase marker reference.

### PCA of pseudobulk RNA-seq

Data were aggregated with the “aggregateData” function in R package to generate a pseudobulk expression matrix from snRNA-seq data. Subsequently, the top 500 most highly variable features (hvgs) were identified. The PCA was performed on scaled data (500 hvgs * samples) with the basic R function “prcomp.”

### Identification of disease-associated CM cell states

Here, we counted the overlapped genes between CM cell state markers and reported heart disease–associated gene sets (*26*). Genes with average log_2_ fold change > 0.3, adjust *P* < 0.01, and expressed in at least 10% of nuclei were regarded as cell state marker genes.

### Milo *k*-nearest neighbor differential abundance testing

To determine the developmental and aging state of each cell within the uniform manifold approximation and projection (UMAP) embedding, we used a method called “MiloR” to perform the differential abundance analysis on *k*-nearest neighbor graph from our single-cell datasets. First, we built graph and neighborhoods using “buildGraph” and “makeNhoods.” Then, we calculated distances, counted cells according to the experimental design, and performed differential abundance testing with “calcNhoodDistance,” “countCells,” and “testNhoods.” Last, we visualized the log_2_–(fold change) from the differential neighborhood abundance testing on the UMAP embedding with the “plotNhoodGraphDA” function.

### Trajectory analysis

To characterize the potential lineage differentiation between cell states of nuclei, we applied the “Monocle” algorithm (version 2). Nuclei were ordered through the inferred pseudotime to indicate their differentiation trajectory. Distinct gene expression patterns that changed among the trajectory branch were identified by the “branched expression analysis modeling” (BEAM) algorithm. Genes with *q* < 1 × 10^–20^ from the BEAM test were used for clustering and GO enrichment analysis.

### Inference of gene-regulatory networks using single-cell regulatory network inference and clustering

“Single-cell regulatory network inference and clustering” (SCENIC) analysis was performed to investigate the transcriptional activity signature of different clusters. Nuclei of each age group were randomly downsampled to 40,000 (keep all the nuclei if there were less than 40,000 nuclei) before extracting normalized expression matrix and phenotype data. The regulons were inferred from TFs and genes that were directly regulated. We analyzed the differences of the AUCell scores of each group with the Wilcoxon rank sum test. Top 10 activated regulons in each group were shown in the heatmap. Differentially activated regulons with *P* <0.05 were selected for further Mfuzz analysis.

### Mfuzz analysis

To reveal the time-relevant gene modules hidden in large gene expression datasets, we performed 15 times of differential expression tests to identify all genes that change between any two groups in AG1 to AG6 (pairwise combinations result in a total of 15 tests). After combining all DEGs from 15 times of tests and removing duplicates, we calculated the average expression level of these genes in AG1-AG6 groups and got the gene expression matrix with genes as rows and age brackets as columns. As to the age-relevant regulon module identification, we calculated the average area under curve (AUC) score of activated regulons in AG1 to AG6 nuclei predicted by “SCENIC” analysis and got a time-course AUC matrix with regulons as rows and time points as columns.

Depending on the constructed time-course gene expression or AUC matrix, the time-series gene modules or regulon modules were identified using the “clusterData” function from the ClusterGVis package, with the “mfuzz” clustering algorithm. Mfuzz was more sensitive to time-series data and more noise robust compared to the other tested algorithms (including “TCseq,” “ImpulseDE2,” and “maSigPro”).

### Correlation analysis

We calculated the average normalized expression level of *PRDM16* and average SASP/aging score of CM nuclei in each sample and performed Spearman correlation analysis. Scatter plots were generated using the “ggscatter” function in the ggpubr package. Each dot in the scatter plots represented one sample in our dataset.

### Cell-cell communication analysis

Cell-cell interactions (CCI) between assigned nuclei population of different comparisons were inferred using CellChat (version 1.1.3). The CellChat database was obtained from the official repository of CellChat (https://github.com/sqjin/CellChat). CCI analysis was performed using the log-transformed normalized gene counts (default settings). Different group data used for intergroup comparisons were used as input to the CCI analysis workflow, and a merged data object was created by the “mergeCellChat” function for further identification of significant CCI changes between the compared groups. The “rankNet” function was used to generate interaction barplot for information flow. The “netAnalysis_signalingRole_heatmap” function in R package was used to represent the relative signaling strength of a signaling pathway across nuclei groups. The “netVisual_circle” function was used to show the strength of the communication between different nuclei groups.

### GSEA analysis

GSEA was performed using the clusterProfiler (version 4.14.6) on user-defined gene sets provided in TERM2GENE format, with multiple testing adjusted for the Benjamini-Hochberg false discovery rate (FDR). Enrichment results were visualized with enrichplot (version 1.26.6) by generating enrichment curves.

### Research ethics of animal experiment

All animal procedures were approved and supervised by the Institutional Animal Care and Use Committee of Fuwai Hospital [approval no. 0108-7-800-ZX(X)-019].

### Mouse intramyocardial injection of adenoviruses

Mice were anesthetized by 2% isoflurane. The left thoracotomy was performed. Adenoviruses (WZ Biosciences) expressing GFP [1 × 10^10^ plaque-forming units (PFU)/ml] or *Prdm16* (1 × 10^10^ PFU/ml) were intramyocardial injected at five sites in the anterior and lateral walls of LV (30 μl per mouse).

### Echocardiography of mouse heart

The cardiac structure and function were evaluated using a Vevo 2100 ultrasound system (VisualSonics) under anesthetic. Real-time two-dimensional B-mode cine loops and M-mode images of short-axis views of the LV were obtained to evaluate cardiac structure and function.

### Preparation of mouse heart nuclei for snRNA-seq

Publicly available embryonic mouse heart snRNA-seq data from the study by Leonard *et al.* (*84*) were included as the mouse age group 1 (mAG1) individual (embryonic day 14.5, wild type, C57BL/6; 10x Genomics Chromium Next GEM Single Cell Multiome Reagent Kit). To align with the human age stages in this study, we included 4-month-old mice (mAG2, male, wild type, C57BL/6, *n* = 3, matching the human age range of 20 to 30 years), 13-month-old mice (mAG3, male, wild type, C57BL/6, *n* = 3, matching the human age range of 40 to 50 years), and 23-month-old mice (mAG3, male, wild type, C57BL/6, *n* = 3, matching the human age range of 60 to 75 years) (*85*).

The 23-month-old aged mice hearts intramyocardially injected with adenoviral control (AD-GFP, male, wild type, C57BL/6, *n* = 3) and adenovirus overexpressing *Prdm16* (AD-*Prdm16*, male, wild type, C57BL/6, *n* = 3) were used for snRNA-seq.

The single nuclei isolation and sequencing procedures were consistent with the described methods of human data preparation. In terms of reagent usage, the mouse hearts of mAG2, mAG3, mAG4, AD-GFP, and AD-*Prdm16* were processed using the Chromium Single Cell 3′ Reagent Kits (v4, 10x Genomics). The snRNA-seq data were aligned to the mouse genome (version refdata-gex-GRCm39-2024-A, 10x Genomics) using the Cell Ranger pipeline (version 9.0.1, 10x Genomics) to obtain raw and filtered feature expression matrices per sample. For quality control, the same processing standards applied to human snRNA-seq data were adopted, with the only modification being the mitochondrial gene threshold, which was adjusted to 25% based on the analytical framework established by Leonard *et al.* (*84*). The subsequent analysis methods for mouse snRNA-seq were consistent with those for human data.

### Immunohistochemical staining of marker of proliferation Ki-67 (Ki67)

Paraffin-embedded heart sections (5-μm-thick) were deparaffinized in xylene and hydrated with an alcohol gradient and tap water. Antigen retrieval was conducted using EDTA solution (ZSJQ-BIO, ZLI-9068). The heart sections were incubated in 3% hydrogen peroxide for 20 min to block endogenous peroxidase activity. Sections were then incubated with goat serum (Beyotime, P0220) for 1 hour to block nonspecific binding. After preparation, sections were incubated with a primary Ki67 antibody (Proteintech, 27309-1-AP) in blocking buffer overnight (4°C). The secondary antibody (ZSJQ-BIO, PV-6001) conjugated to anti-rabbit horseradish peroxidase (HRP) was then incubated for 1 hour (room temperature). DAB Staining Kit (ZSJQ-BIO, ZLI-9019) was then used according to the manufacturer’s protocol. Whole slide images were captured using an automatic digital slide-scanning system (Hamamatsu, NanoZoomer S60).

### Immunofluorescence staining and opal multiplex staining

Paraffin-embedded sections (5-μm-thick) were baked at 68°C for 45 min and then deparaffinized. Antigen retrieval was performed in the EDTA solution (ZSJQ-BIO, ZLI-9068) with pH 9.0 for 90 s, followed by cooling to room temperature. Sections were washed three times with PBS solution (Gibco, C10010500BT) for 5 min. Blocking solution (Beyotime, P0220) was used for incubation for 1 hour. Sections were incubated with primary antibodies in blocking buffer overnight at 4°C. Samples were then washed three times with phosphate buffered saline with Tween 20 solution for 5 min. Wheat germ agglutinin (WGA) staining (Sigma-Aldrich, L4895) was used for incubation for 30 min, followed by PBST washing three times for 5 min. Immunofluorescence secondary antibodies (Thermo Fisher Scientific, A-11008, A-11001, A-21428, and A-21422) were used for incubation for 1 hour, followed by PBST washing three times for 5 min. Sections were mounted with a 4′,6-diamidino-2-phenylindole (DAPI)–containing mounting medium (MedChemExpress, HY-K1047) and scanned using an automatic digital slide-scanning system (Hamamatsu, NanoZoomer S60). The semiquantitative analyses of relative fluorescence density were performed using the ImageJ software [version 1.53, National Institutes of Health (NIH)]. Opal multiplex staining was performed according to the manufacturer’s protocol: (i) slide preparation; (ii) epitope retrieval; (iii) blocking; (iv) incubation with primary antibody; (v) incubation with HRP-conjugated secondary antibody; (vi) signal amplification; (vii) antibody stripping via microwave treatment. For detection of the next target protein, the protocol was restarted at step 3. After all targets were detected, counterstaining and mounting were performed. The slides were analyzed by Vectra Quantitative Pathology Imaging Systems (PerkinElmer).

### Antibodies for immunohistochemical and immunofluorescence staining

Anti-Ki67 (Proteintech, 27309-1-AP; 1:1000 dilution for immunohistochemical; 1:100 dilution for immunofluorescence staining), anti-myosin binding protein C3 (MYBPC3) (Proteintech, 67608-1-Ig; 1:200 dilution), anti-PRDM16 (Affinity, DF13303; 1:100 dilution), anti-crystallin alpha B (CRYAB) (Proteintech, 15808-1-AP; 1:50 dilution), anti-CD31 (Proteintech, 28083-1-AP; 1:8000 dilution), anti-CD74 (Abcam, ab9514; 1:50 dilution), anti-apolipoprotein D (AOPD) (Proteintech, 10520-1-AP; 1:100 dilution), anti-vimentin (VIM) (Proteintech, 60330-1-Ig; 1:500 dilution), anti-CD45 (Proteintech, 20103-1-AP, 1:1000 dilution), and anti-myosin heavy chain 11 (MYH11) (Proteintech, 21404-1-AP; 1:50 dilution).

### Masson’s trichrome staining

Paraffin-embedded sections (5-μm-thick) were prepared by deparaffinization and rehydration. Sections were rinsed and stained with potassium dichromate solution overnight (room temperature). Sections were stained with iron hematoxylin solution for 5 min (room temperature), followed by staining with ponceau acid fuchsin solution for 5 min (room temperature). Sections were incubated in the phosphomolybdic-phosphotungstic acid solution for 2 min (room temperature) and stained in aniline blue solution for 2 min (room temperature). After being rinsed with distilled water, sections were dehydrated and mounted. The staining section images were obtained using an automatic digital slide-scanning system (Hamamatsu, NanoZoomer S60). Image-Pro Plus (Version 6.0, Media Cybernetics) was used to semiquantify the area ratio of CMs (red), adipose tissue (white), and fibrosis (blue).

### Hematoxylin and eosin staining

Paraffin-embedded sections (5-μm-thick) were deparaffinized using xylene, hydrated in gradient alcohols, stained with hematoxylin solution, rinsed with distilled water, and differentiated with 1% acid alcohol. Sections were stained with eosin, dehydrated in graded ethanol, rendered transparent with xylene, and mounted. The staining sections were scanned using an automatic digital slide-scanning system (Hamamatsu, NanoZoomer S60).

### Cell culture and maintenance

AC-16 (American Type Culture Collection, CRL-3568) cells were cultured in Dulbecco’s modified Eagle’s medium (Gibco, 11965126) mixed with 10% fetal bovine serum (Gibco, A5669401) in 5% CO_2_ at 37°C. The hiPSCs were derived from a healthy Asian female, 30 years old. hiPSCs were cultured in mTeSR Plus medium (STEMCELL Technologies, 100-0274), and the cell culture plates were coated with Corning Matrigel hESC-Qualified Matrix (Corning, 354277). After hiPSC expansion, hiPSCs were induced to differentiate to cardiomyocytes (hiPSC-CMs) according to the following steps: iPSCs were cultured for 4 days, at which point they reached ∼80% confluence. iPSCs were dissociated into single-cell suspension using the Gentle Cell Dissociation Reagent (STEMCELL Technologies, 07174) and cultured in 12-well plates at a density of 5 × 10^5^ in a 5% CO_2_ incubator at 37°C for 24 hours. Twenty-four hours later, cells were differentiated using an RPMI 1640–based medium (Thermo Fisher Scientific, 11875), supplemented with 55 μM β-mercaptoethanol, 2-phospho-l-ascorbic acid trisodium salt (64 μg/ml), bovine serum albumin (BSA) (Sigma-Aldrich, A3311), glutamax, bone morphogenetic protein 4 (BMP4; 10 ng/ml), activin A (10 ng/ml), and 4 μM CHIR99021 for 24 hours. Twenty-four hours later, the medium was completely replaced with an RPMI 1640–based medium (Thermo Fisher Scientific, 11875), supplemented with 55 μM β-mercaptoethanol, 2-phospho-l-ascorbic acid trisodium salt (64 μg/ml), BSA, and glutamax. Forty-eight hours later, the medium was replaced with an RPMI 1640–based medium (Thermo Fisher Scientific, 11875), supplemented with 55 μM 2-mercaptoethanol, 2-phospho-l-ascorbic acid trisodium salt (64 μg/ml), BSA, glutamax, BMP4 (10 ng/ml), fibroblast growth factor 2 (8 ng/ml), 2 μM Wnt-C59, and 0.5 μM retinoic acid. After 4 days, the hiPSC-CMs began beating and cultured in hiPSC-CM–specific medium (Guidon Pharmaceutics).

### Senescence induction in hiPSC-CMs

The hiPSC-CMs were treated with 0.1 μM doxorubicin (Selleck, S1208) or 500 μM H_2_O_2_ (Sigma-Aldrich, 88597) for 24 hours to induce senescence (*41*).

### Characterization by flow cytometry

Digested single-cell hiPSC-CMs were divided into three equal portions, with ∼7.5 × 10^5^ cells per portion. One fraction served as a blank control (BC) group, one as a control group with only secondary antibodies (negative control; NC), and the tested group. We added Dulbecco’s phosphate-buffered saline (DPBS) (100 μl; Thermo Fisher Scientific, 14190144) to the BC fraction and added 0.25 μl of LIVE/DEAD red (Thermo Fisher Scientific, L3224) in DPBS system to the NC and tested fraction. Suspensions were mixed and incubated at 4°C for 30 min (keep in dark place). Incubaion was terminated using 1% BSA solution, followed by a washing step using a centrifuge (4°C, 2300 rpm, 1 min). Fixation/Permeabilization Concentrate (1×, 200 μl; Thermo Fisher Scientific, 00-5123-43) was added in each group and fixed for 30 min (room temperature, keep in dark place). After centrifugation (4°C, 2300 rpm, 1 min), the cell pellet was resuspended in Permeabilization Buffer (1×, 200 μl, Thermo Fisher Scientific, 00-8333-56). For the tested group, Anti-Cardiac Troponin T (Abcam, ab209813; 1:500 dilution) was added into Permeabilization Buffer system. The cell pellet was retained and mixed with Permeabilization Buffer (1×, 100 μl) before incubation with Permeabilization Buffer for 30 min (room temperature). For NC and tested fractions, Goat Anti-Rabbit IgG-AF488 (SouthernBiotech, 4030-30; 1:1000 dilution) was added into the Permeabilization Buffer. After incubation with the Permeabilization Buffer for 30 min (room temperature), the cell pellet was retained and mixed with BSA solution (1%, 200 μl). Flow cytometric experiments were conducted on the Cytek Flow Cytometry System (Cytek, NL-CLC B14; Software: SpectroFlo Version 1.3.3).

### siRNA transfection system

The AC-16 cells and hiPSC-CMs were cultured in 12- or 24-well plates for 24 hours before transfection treatment. Transfection complexes were preformed with the siRNAs (RiboBio, SIGS0012946-4) and Lipofectamine RNAiMAX Transfection Reagent (Thermo Fisher Scientific, 13778150) and Opti-MEM (Thermo Fisher Scientific, 31985070). The final concentration of oligonucleotides in treated cells was 50 nM. The groups included the blank negative control group (NC), the positive control group transfected with siRNA-*GAPDH* (siPC), the negative control group transfected with non-targeting siRNA (siNC), and the experimental groups transfected with siRNA-*PRDM16*.

### RNA isolation and real-time quantitative polymerase chain reaction

Total RNA of cells was isolated using the TRIzol Reagent (Thermo Fisher Scientific, 15596018) according to the manufacturer’s instructions. cDNA was prepared from 500 ng of total RNA using the cDNA synthesis kit (Takara, RR036A). Real-time quantitative polymerase chain reaction (RT-qPCR) was conducted with the PowerUp SYBR Green Premix (Thermo Fisher Scientific, A25742). Expression of each target mRNA was calculated relative to that of *RPL5* based on the threshold cycle (*C*_t_), as *r* = 2^−∆∆*C*t^. The primer design of the target genes was based on University of California, Santa Cruz (UCSC) Genome Browser (https://genome.ucsc.edu/index.html). RT-qPCR was performed with QuantStudio 5 Real-Time System (Thermo Fisher Scientific, A28140). *PRDM16*-primer-1, 5′-GCTGGCTCAAGTACATCCGT-3′ (forward) and 5′-TCACCTGGCTCAATGTCCTT-3′ (reverse); *PRDM16*-primer-2, 5′-AGTGCACCTGGGATGACAAA-3′ (forward) and 5′-ACAGGTTGGAGAACTGCGTG-3′ (reverse); *RPL5*, 5′-TCGTATAGCAGCATGAGCTTTC-3′ (forward) and 5′-TGTTGCAGATTACATGCGCT-3′ (reverse); *GAPDH*, 5′-AGAAGGCTGGGGCTCATTTG-3′ (forward) and 5′-AGGGGCCATCCACAGTCTTC-3′ (reverse).

### Western blotting

Total protein was extracted using radioimmunoprecipitation assay (Beyotime, P0013B) and then quantified using the bicinchoninic acid protein assay kit (Thermo Fisher Scientific, 23227). The protein solution was separated using SDS–polyacrylamide gel electrophoresis gels (4 to 12%) and electrotransferred to nitrocellulose membranes using the eBlot L1 Fast Wet Transfer System (GenScript, L00686C). After incubation with the blocking buffer, membranes were probed with primary antibodies and subsequently incubated with a secondary antibody conjugated with HRP. Visualization of the blots was achieved through chemiluminescence (Thermo Fisher Scientific, A38555, for anti-PRDM16 and anti-p21 blots; Epizyme Biotech, SQ101L, for anti–β-actin blots). The semiquantitative analyses of blots were performed using the ImageJ software (version 1.53, NIH).

### Antibodies for Western blotting

Anti-PRDM16 (Proteintech, 55361-1-AP; 1:500 dilution), anti-p21 (Proteintech, 10355-1-AP; 1:1000 dilution), and anti–β-actin (Proteintech, HRP-60008; 1:5000 dilution).

### Enzyme-linked immunosorbent assay

The protein levels of IL-8 in the culture medium were assessed using the Highly Sensitive Human IL-8 ELISA kit (Elabscience, E-HSEL-H0004) following the manufacturer’s instructions. Plates were scanned at an optical density of 450 nm with a Synergy H1 microplate reader (BioTek). Sample duplicates were averaged for each standard and sample, and then the average zero standard optical density was subtracted.

### SA-β-Gal staining

The activity of SA-β-gal of cells was determined by the Senescence β-Galactosidase Staining Kit (Cell Signaling, 9860) following the manufacturer’s instructions. Images were acquired with an All-in-One Microscope (Keyence, BX-X800LE), and the percentage of SA-β-gal–positive cells was calculated by the ImageJ software (version 1.53, NIH).

### BrdU assay

BrdU (Sigma-Aldrich, B5002-1G) was used to assess the cell cycle. After incubating the cells with BrdU, detection was performed using a BrdU detection kit (Applygen, B3035) according to the manufacturer’s instructions. Fluorescence was observed using a microscope (Zeiss, Axio Observer D1), and the percentage of BrdU-positive nuclei was calculated by the ImageJ software (version 1.53, NIH).

### Phalloidin staining

The phalloidin staining kit (Yeasen, 40734ES75) was used according to the manufacturer’s protocol. Cells were fixed with 4% formaldehyde solution for 10 min (room temperature) and then stained with phalloidin solution after fixation and rinsing. Fluorescence signals were acquired under an All-in-One Microscope (Keyence, BX-X800LE). The cross-sectional areas of cells were calculated using ImageJ (version 1.53, NIH).

### Measurements of mitochondrial function by Seahorse

Mitochondrial function was assessed by the level of oxygen consumption rate (OCR). OCR was evaluated using the Seahorse XF Cell Mito Stress Test Kit (Agilent, 103015-100) and the Seahorse XFe24 Analyzer (Agilent, 102342-100). Different reagents were added as follows: 1.5 μM oligomycin, 1 μM carbonyl cyanide *p*-trifluoromethoxyphenylhydrazone (FCCP), and 0.5 μM rotenone/antimycin A.

### RNA-seq analysis of hiPSC-derived CMs

RNA-seq was performed to investigate the transcriptional profile of *PRDM16*-knockdown hiPSC-derived CMs. Cell samples treated with siRNA-negative control (siNC) (five biological replicates) and si*PRDM16*#1 (five biological replicates) were included. Total RNA of cell samples was extracted by TRIzol Reagent (Thermo Fisher Scientific, 15596018). RNA integrity was confirmed by Bioanalyzer Agilent 5400 system and used for RNA library construction using NEBNext Ultra RNA Library Prep Kit for Illumina (New England Biolabs, E7770).

Differential expression analysis was conducted using DESeq2 (version 1.44.0), and genes were filtered for an adjusted *P* value threshold of <0.05. Volcano plot was generated using the ggplot2 package (version 3.5.2), and PCA was performed using the DESeq2 package. GO enrichment analysis was carried out with clusterProfiler (version 4.12.6). Gene set scores related to aging, SASP, and dilated cardiomyopathy were calculated using GSVA (version 1.52.3).

### Construction of single-nucleus heart age prediction model

To predict the gestational week or age of the heart from transcriptional features, pseudobulk gene expression profiles were generated from snRNA-seq. For the construction of the gestational week prediction models for fetal hearts, the snRNA-seq data included the internal data of this study and the publicly available nonfailing fetal heart data from Sim *et al.* (*86*). For the construction of the age prediction models for nonfetal hearts, the snRNA-seq data included the internal data of this study and publicly available nonfailing adult heart data from the Human Cell Atlas project (*1*, *2*, *87*). The snRNA-seq data from diseased cohorts were obtained and used to evaluate perturbations of heart transcriptional age under disease conditions (*2*). Then, nuclei encompassing all major cell types were randomly partitioned into 60 subsets within each individual, and gene expression was averaged in each subset to generate pseudobulk samples. This approach mitigated single-nucleus variability while capturing age-related transcriptional patterns.

Multiple regression models were trained using the XGBoost algorithm (version 2.1.4) with a squared error objective, implemented via the scikit-learn interface (version 1.6.1). The pseudobulk samples were randomly partitioned into training (70%) and test (30%) sets, where each pseudobulk subset was treated as an independent sample without imposing individual-level constraints on the train-test split. Model selection and performance evaluation were performed within the training sets through 10-fold cross-validation. Hyperparameter tuning was conducted using grid search, and the optimal model was selected on the basis of the highest Pearson correlation coefficient between predicted and chronological gestational week or age during cross-validation. The contribution importance of the genes to the models were identified and ranked, and GO enrichment analysis was performed on the top 100 genes by the R package clusterProfiler (version 4.1.0). The selected optimal models were then applied to the test sets and to independent diseased cohorts for heart age prediction.

### Statistics and reproducibility

Representative micrographs in Figs. 2D, 5D, and 6D and figs. S4A, S6A, S8 (A and B), S11 (D and E), S14 (A and B), S18 (A and B), and S21F were selected from the scanned slides for AG1 group (LV, *n* = 5; RV, *n* = 5), AG2 group (LV, *n* = 5; RV, *n* = 5), AG3 group (LV, *n* = 6; RV, *n* = 6), AG4 group (LV, *n* = 3; RV, *n* = 3), AG5 group (LV, *n* = 3; RV, *n* = 3), and AG6 group (LV, *n* = 5; RV, *n* = 5). For non–snRNA-seq analyses, for two-group comparisons, Shapiro-Wilk tests were used to identify the data normality. According to the characteristics of data normality and variance homogeneity, two-tailed *t* tests and Mann-Whitney tests were used, respectively. Multiple-group comparisons were made by analysis of variance (ANOVA)/Kruskal-Wallis and Tukey/Dunnett tests. Holm-Bonferroni method was used to control for type I error for multiple comparisons. Statistical analyses of non–snRNA-seq were performed by the SPSS software (version 23.0, IBM Corp.) and GraphPad Prism (version 8.0.2, GraphPad Software). Statistical significance was accepted if *P* < 0.05.

## Supplementary Materials

### The PDF file includes:

Figs. S1 to S30 Legends for tables S1 to S55 Uncropped image of Fig. 3G Uncropped image of fig. S13E
