# Supplementary material for: Life-span–dependent transcriptional dynamics of the human heart
Source: Sci Adv. 2026 Jun 17;12(25):eaeg2614. doi: 10.1126/sciadv.aeg2614 (PMC13274588; doi:10.1126/sciadv.aeg2614)

Science Advances
AAAS

Supplementary Materials for

Life-span–dependent transcriptional dynamics of the human heart

Hao Jia *et al.*

Corresponding author: Daniel Reichart, daniel.reichart@med.uni-muenchen.de;
Jiangping Song, fwsongjiangping@126.com

Sci. Adv. **12**, eaeg2614 (2026)
DOI: 10.1126/sciadv.aeg2614

The PDF file includes:

Figs. S1 to S30
Legends for tables S1 to S55
Uncropped image of Fig. 3G
Uncropped image of fig. S13E

Other Supplementary Material for this manuscript includes the following:

Tables S1 to S55

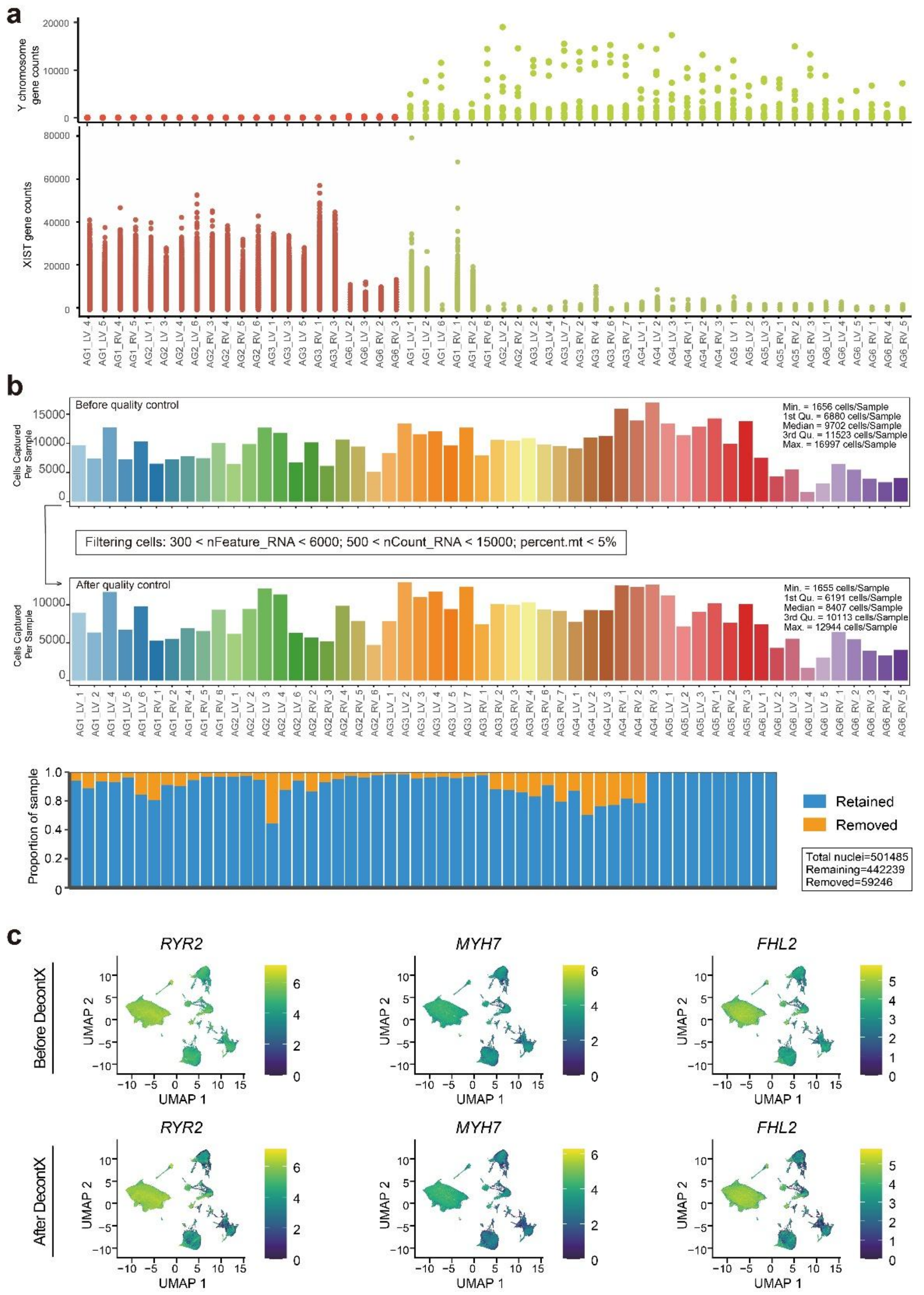

Fig. s1 | Gender analyses and sample quality control assessment. a. Total counts of Y chromosome (top) and *Xist* (bottom) transcripts classifying females (red) and males (green) across all samples (n = 54). **b.** The number of nuclei per sample detected before (top) and after (middle) quality control. Min, minimum value; 1st Qu, lower quartile; 3rd Qu, upper quartile; Max, maximum value. Proportion of retained and removed nuclei per sample (n=54) after quality control (bottom). **c.** Contamination (top) and decontamination (bottom) of ambient RNA using DecontX.

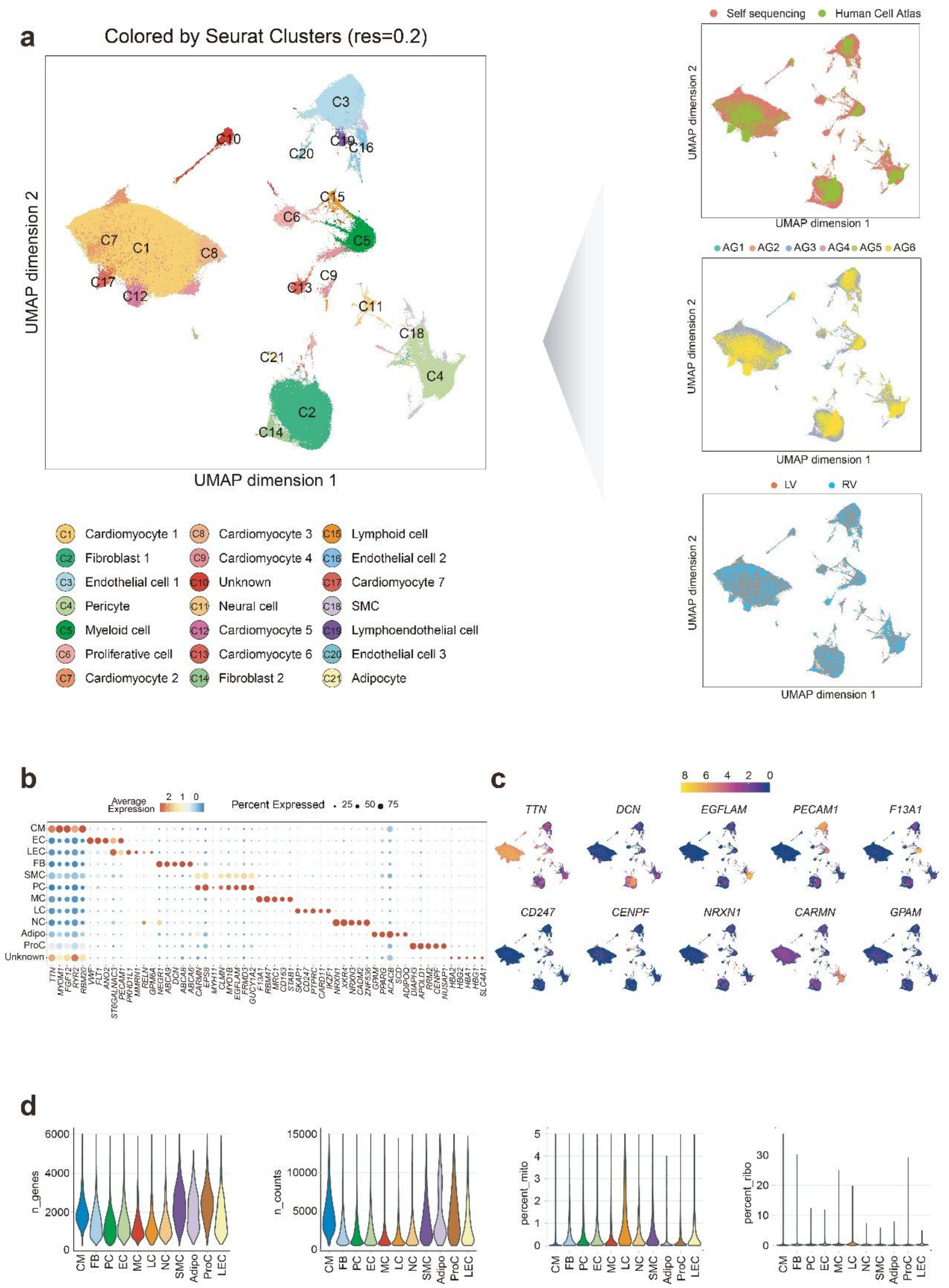

Fig. s2 | Cardiac tissue snRNA-seq profiling. a. Left: Uniform manifold approximation and projection (UMAP) plots embedding of 442,239 single nuclei delineated the main cell clusters. Cell type annotations were added in addition. UMAPs of nuclei were colored by data sources (right-top), age group assignments AG1-6 (right-middle), and anatomical sites LV vs. RV (right-bottom). **b.** Dot plots visualizing the marker genes of each major cardiac cell type. CM, cardiomyocyte; EC, endothelial cell; LEC, lymphatic endothelial cell; FB, fibroblast; SMC, smooth muscle cell; PC, pericyte; MC, myeloid cell; LC, lymphoid cell; NC, neural cell; Adipo, adipocyte; ProC, proliferative cell. **c.** Feature

plot highlighting marker genes of each major cardiac cell type. **d.** Violin plots depicting the number of genes (n_genes), number of UMIs (n_counts), percent UMIs mapping to mitochondrial genes (percent_mito), and percent UMIs mapping to ribosomal genes (percent_ribo) stratified by each major cell type.

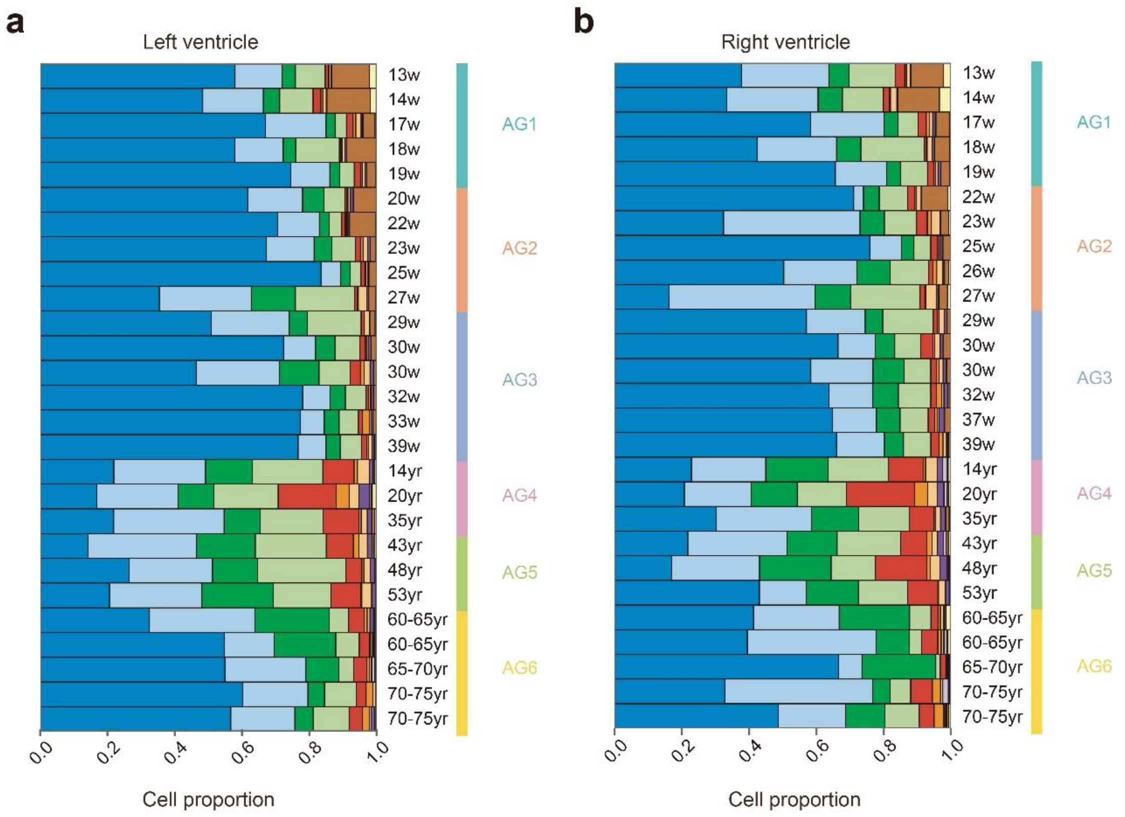

Fig. s3 | Cellular abundance in the left ventricle (LV) and right ventricle (RV). Stacked bar plot depicting the cell type proportion of each sample (n = 54) and age groups AG1-6 with color coding reflecting the major cardiac cell type visualized in **Fig. 1A.** LV **(a)**, RV **(b)**.

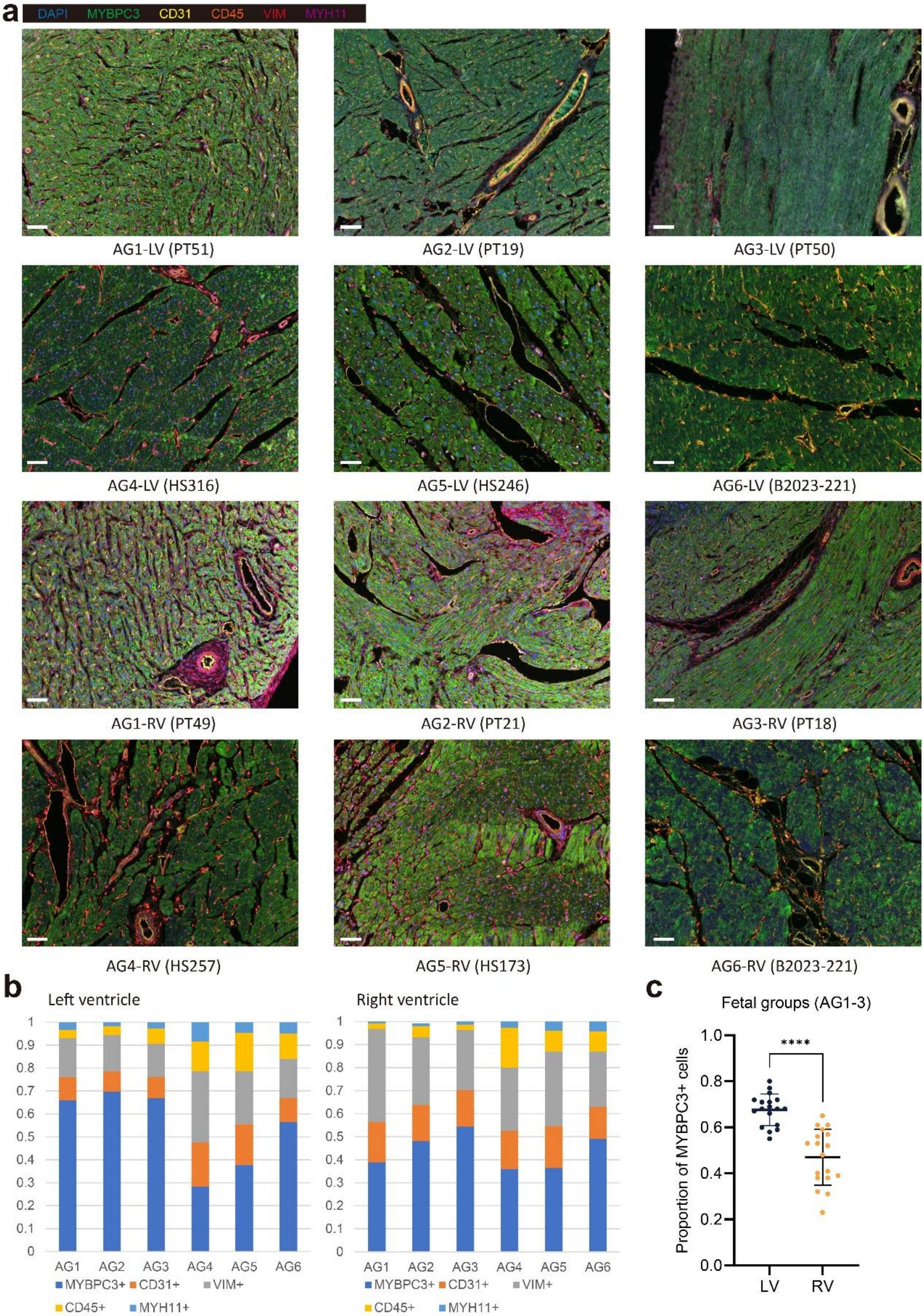

Fig. s4 | Opal multiplex immunohistochemistry staining of major cell types. a. Representative images of opal multiplex immunohistochemistry staining across the 6 age groups. Scale bars, 50 μm. **b.** Proportion of major cell types across all 6 age groups AG1-6. **c.** Proportion of MYBPC3+ CMs in fetal groups AG1-3, significance was tested with two-tailed t test, data were shown as Mean ± SD. **** *P* value < 0.0001.

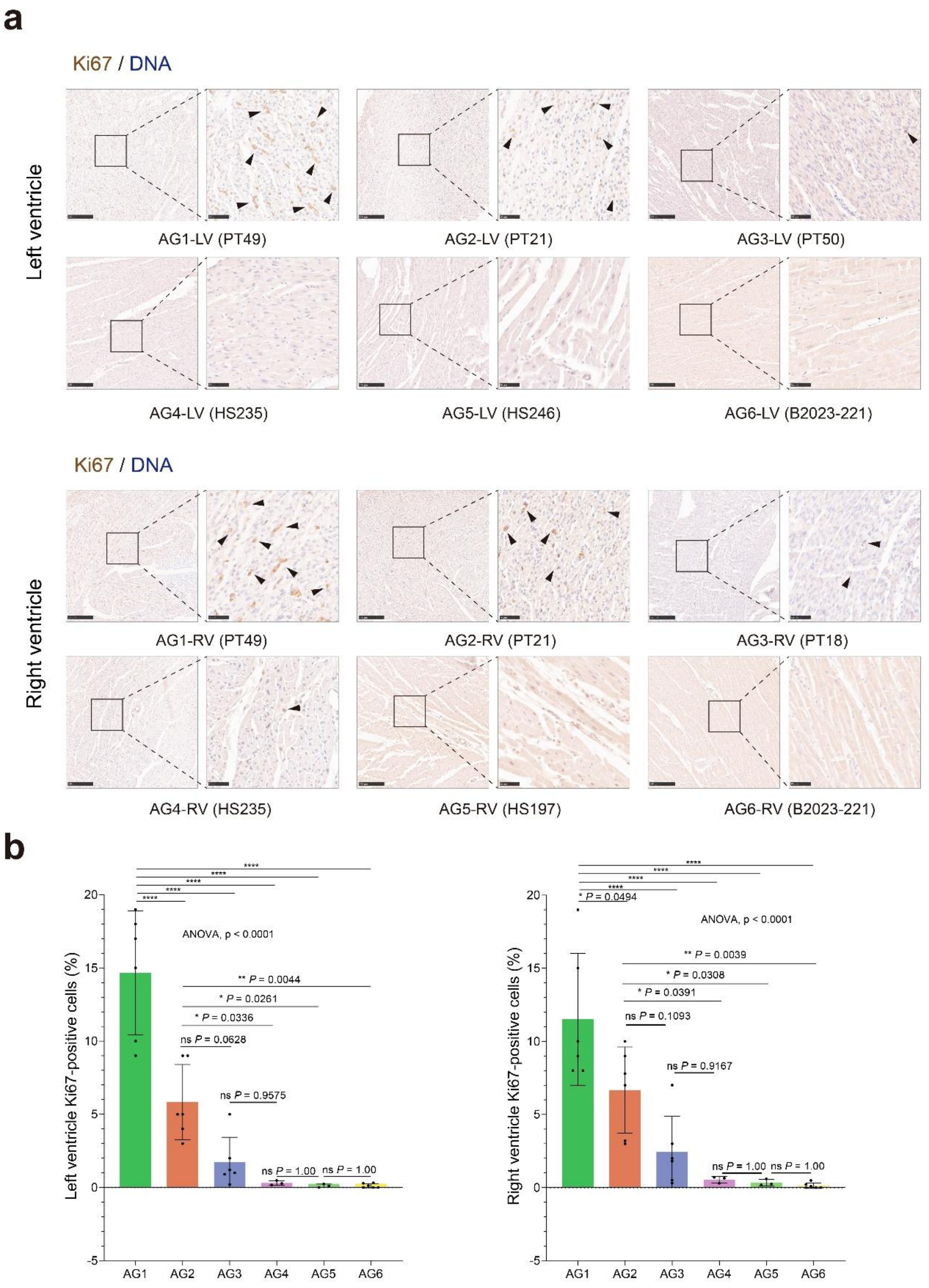

Fig. s5 | Immunohistochemical analysis of Ki67. a. Representative images of Ki67 staining across the 6 age groups. AG1-6 Scale bars, 250 μm (left) and 50 μm (right, magnification). **b.** Quantification of Ki67-staining positive cell. Significance calculated by ANOVA test followed Tukey tests. Data were shown as Mean ± SD. All statistically significant inter-group comparisons were annotated, not annotated meant no significance. * *P* value < 0.05; ** *P* value < 0.01; **** *P* value < 0.0001; no significant difference *P* value > 0.05.

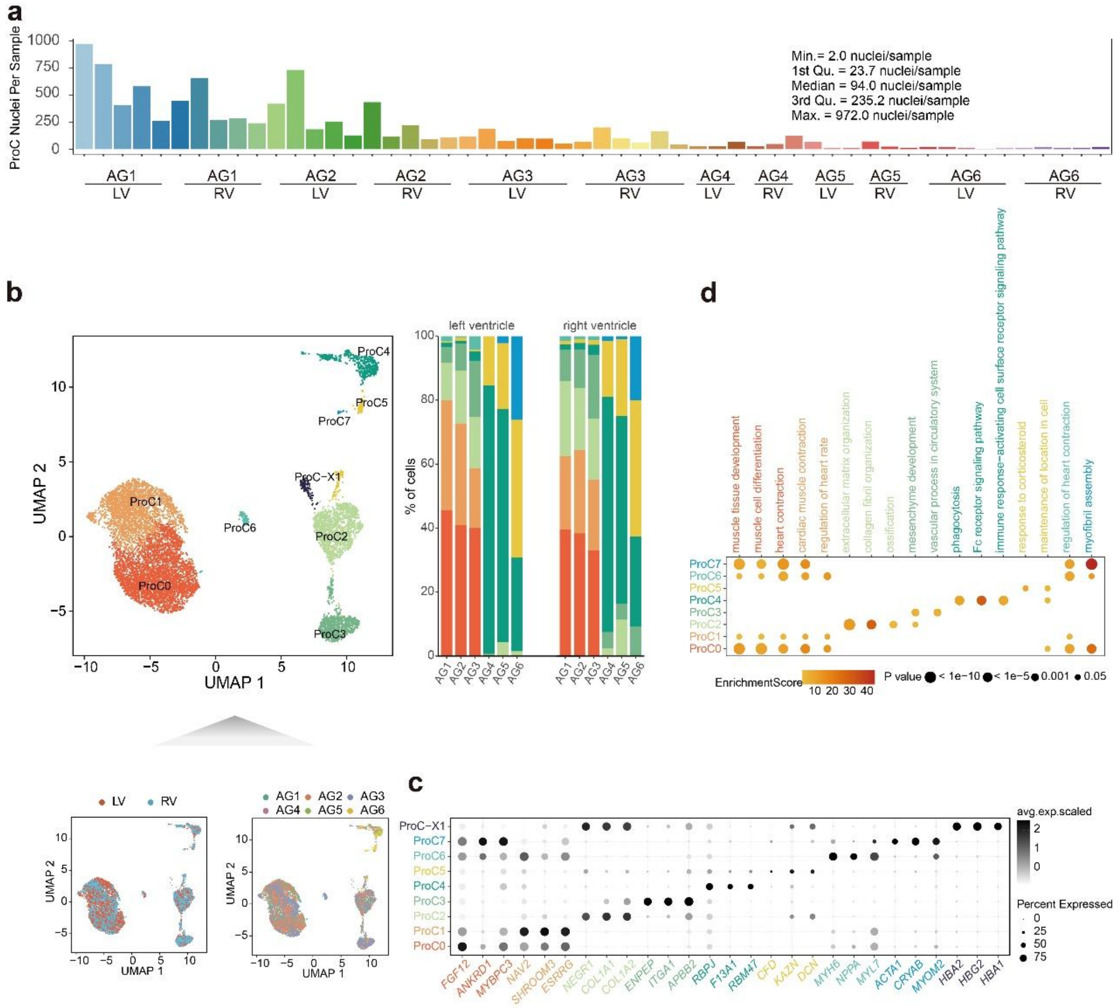

Fig. s6 | Characterization of proliferative cells (ProC). a. ProC nuclei number per sample. **b.** Left: UMAP plot depicting the subclustering and cell state annotations of the ProC population (n = 9,502 excluding ProC-X1). Bottom: UMAPs of ProC were colored by the anatomical sites LV vs. RV (bottom-left) and age group assignment AG1-6 (bottom-right). Proportion of ProC cell states across all 6 age groups AG1-6 (right). **c.** Dot plot depicting Gene Ontology (GO) biological process enrichment for each ProC cell state. **d.** Dot plots showing the marker genes of each ProC cell state.

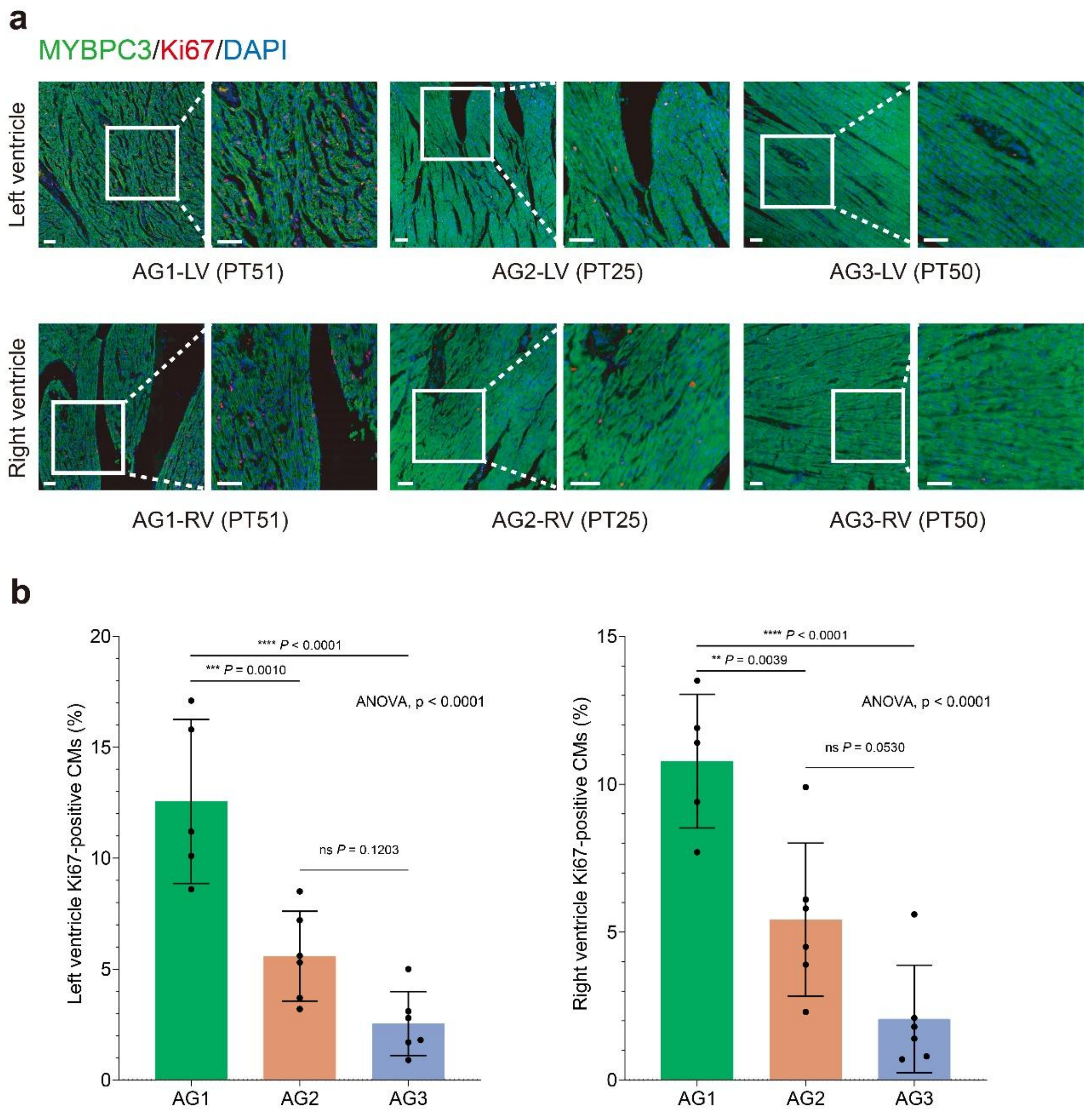

Fig. s7 | Immunostaining images of CM-progenitor cell. a. Representative images of MYBPC3/Ki67/DAPI staining across the 3 fetal age groups. Scale bars - 50 μm. **b.** Quantification of Ki67-staining positive CMs, n=6 per group, biological replicates. Significance calculated by ANOVA test followed Tukey tests. Data were shown as Mean ± SD. ** *P* value < 0.01; *** *P* value < 0.001; **** *P* value < 0.0001; no significant difference *P* value > 0.05.

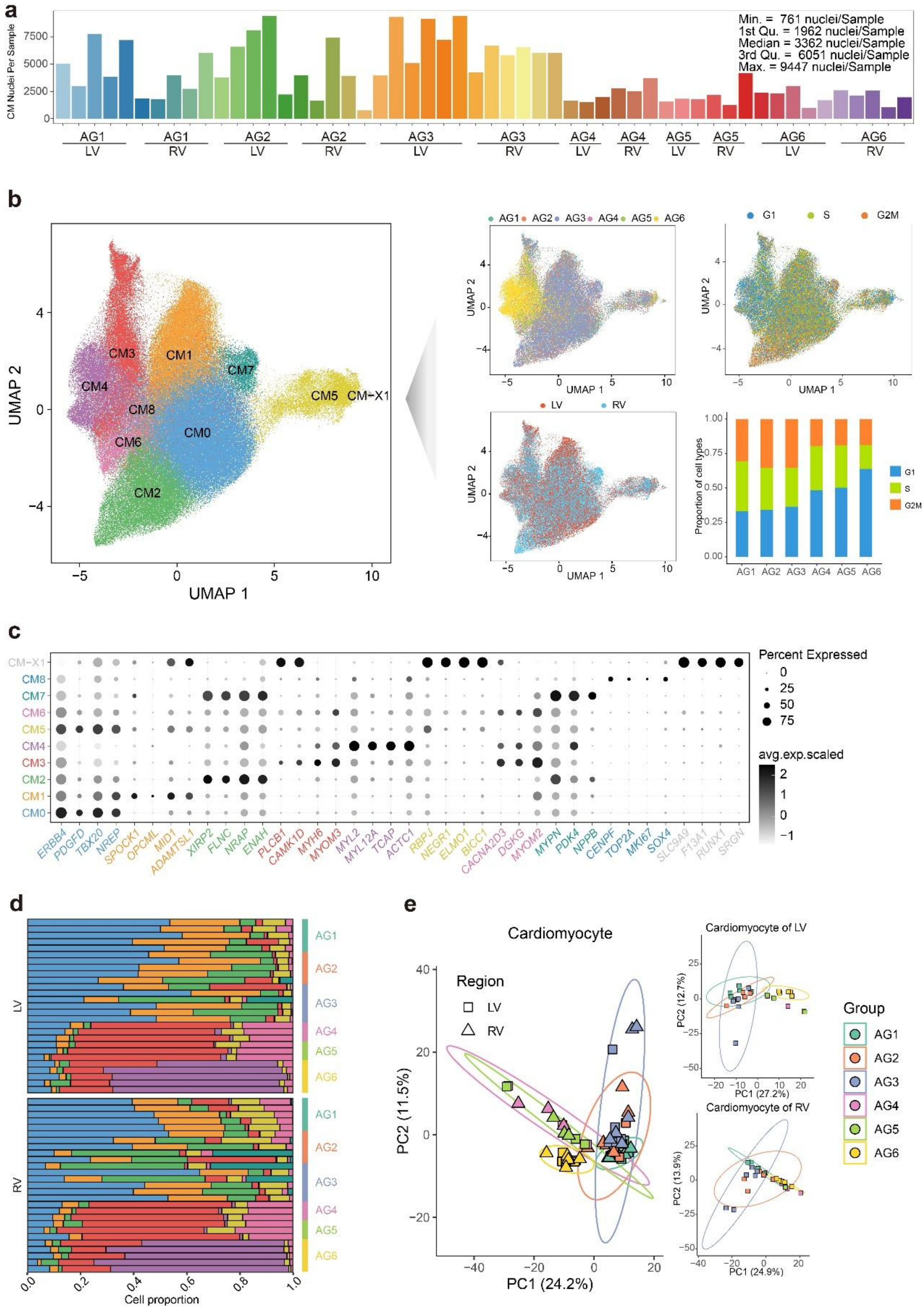

Fig. s8 | Characterization of cardiomyocytes (CM). a. CM nuclei number per sample. **b.** Left: UMAP plot depicting the subclustering and cell state annotations of the CM lineage (n = 218,404 excluding CM-X1). UMAPs of CM nuclei were colored by the age group assignment AG1-6 (middle-top), anatomical sites LV vs. RV (middle-bottom), and cell cycle phases (right-top). Composition of each cell cycle phase stratified by the age groups AG1-6 (right-bottom). **c.** Dot plots showing the marker genes of each CM cell

state. **d.** Stacked bar plot depicting the cell type composition of each sample (n = 54) with color coding reflecting each CM cell state. Colored according to **(b)**. **e.** PCA plots of pseudo-bulk RNA-seq analyses including all CM nuclei.

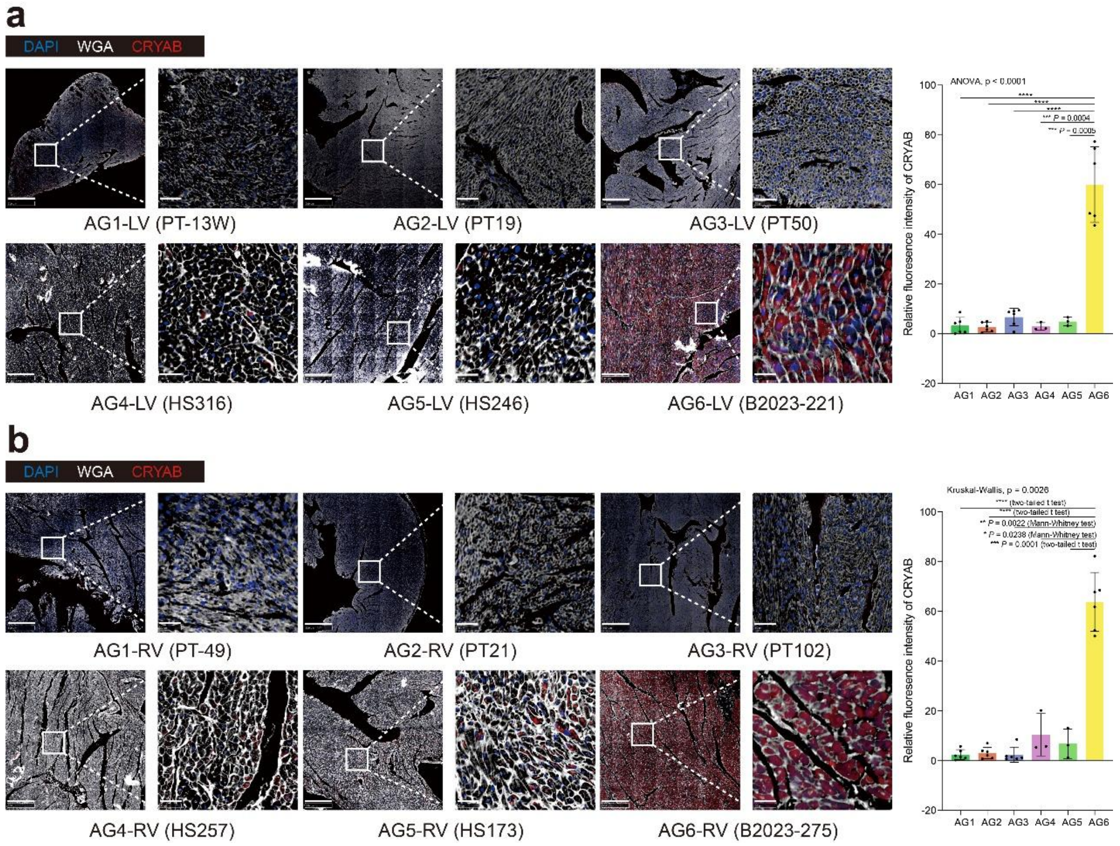

Fig. s9 | Additional immunostaining images of CRYAB expression. Representative images of CRYAB/WGA/DAPI staining across the 6 age groups AG1-6 (left). Scale bars, 500 μm and 50 μm (Magnification). Quantification and statistical analysis of the previous images (right). Depending on data normality, ANOVA test or Kruskal-Wallis test was used. Two-tailed t tests or Mann-Whitney tests were used to compare AG6 with other groups. Data were shown as Mean ± SD. * *P* value < 0.05; ** *P* value < 0.01; *** *P* value < 0.001; **** *P* value < 0.0001; no significant difference *P* value > 0.05. **a.** Left ventricle, the representative images for AG5 and AG6 were reused from **Fig. 2D**. **b.** Right ventricle.

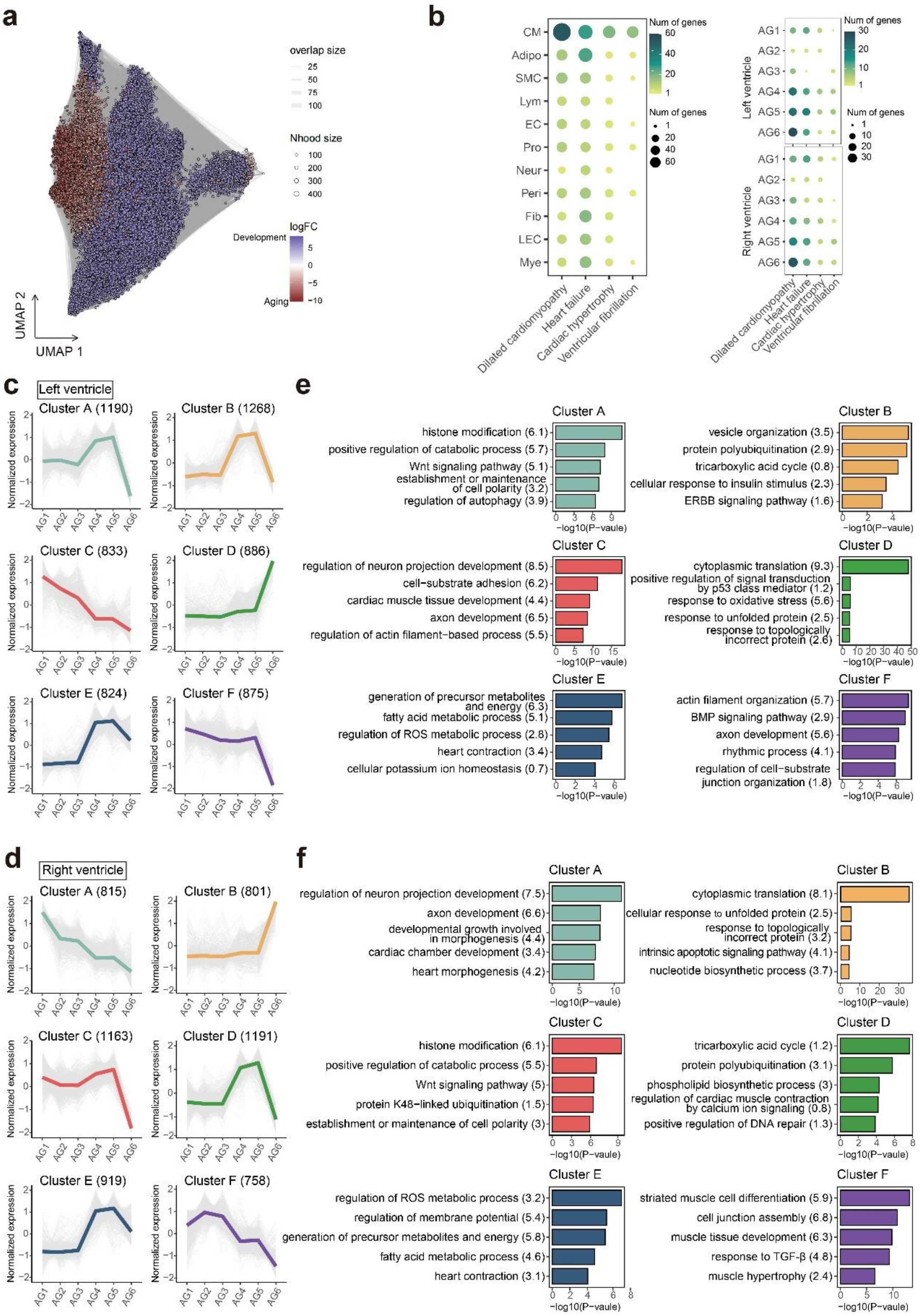

Fig. s10 | SnRNA-seq showing the unique transcriptional signature of cardiomyocytes (CM) across lifespan. a. Embedding of Milo *k*-nearest neighbour differential abundance testing in CMs. All nodes represented neighbourhoods. The layout of nodes was determined by the UMAP embedding, as shown in **Fig. s8b**. **b.** Left: Dot plot depicting the number of genes that overlap between marker genes of each main

cell types and cardiovascular disease-associated genes. Right: Dot plot depicting the number of genes that overlap between the marker genes of each group and the cardiovascular disease- associated gene sets. **c, d.** Feature pattern curves by Mfuzz depicting dynamic gene expression changes across all 6 age groups in CM of LV **(c)** and RV **(d)**. **e.** Six gene clusters (based on dynamic gene expression changes in **(c)**) were identified using GO analysis. **f.** Six gene clusters in (based on dynamic gene expression changes in **(d)**) were identified using GO analysis. Numbers in parentheses in **c** and **d** represented the number of genes included in analyses. Numbers in parentheses in **e** and **f** represented the GO term gene ratio.

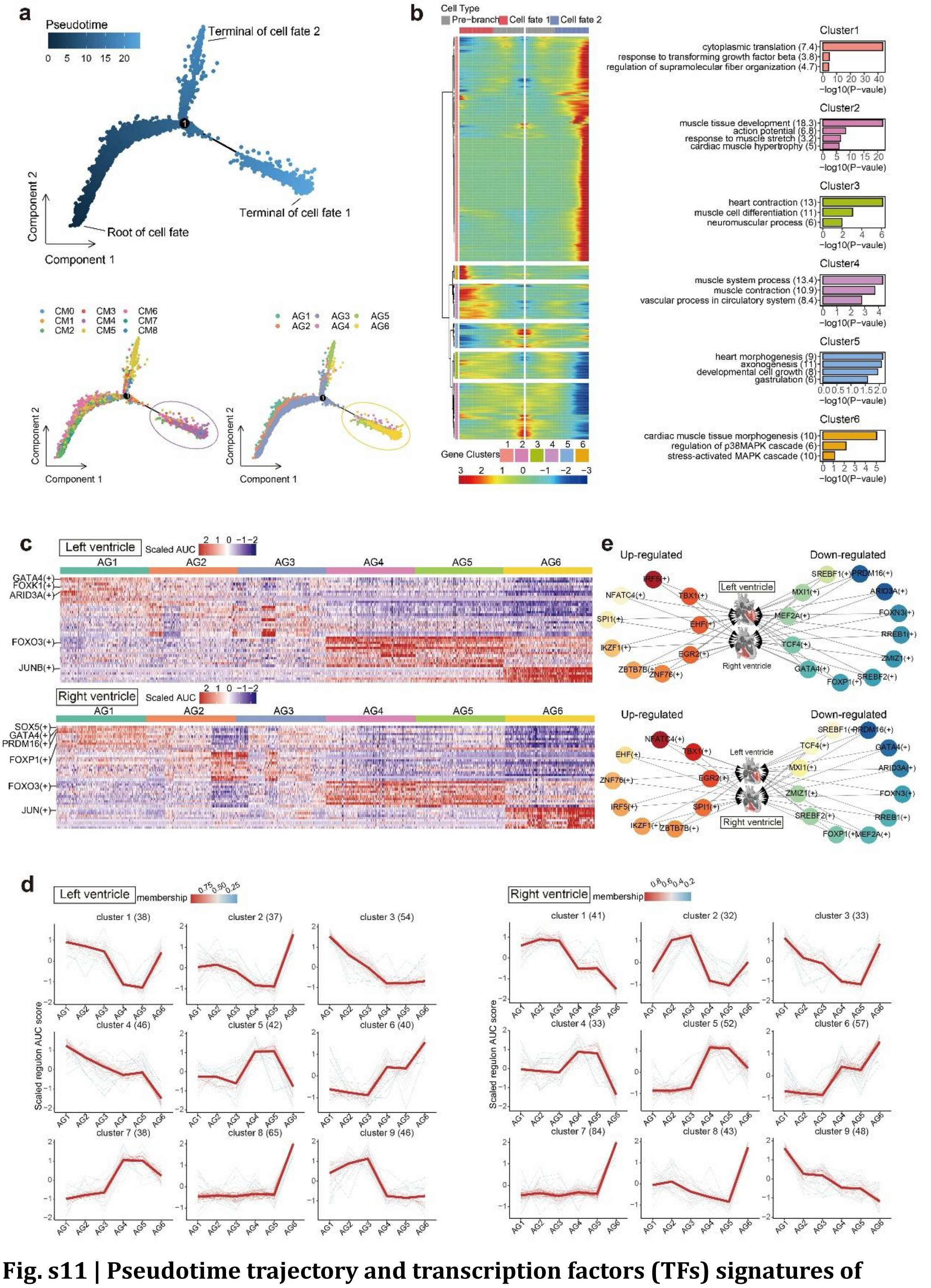

Fig. s11 | Pseudotime trajectory and transcription factors (TFs) signatures of cardiomyocytes (CM). a. Pseudotime trajectory of CMs inferred by using monocle 2. Circles represented the regions where CM4 and AG6 groups were located. **b.** Heatmap showing differentially expressed genes (DEGs) alone with the pseudotime as in **(a)**, cataloged into six gene module clusters (left). Six gene clusters were identified with GO analysis (right). Numbers in parentheses represented the gene ratio for the GO terms. **c.** Heatmap depicting the Area Under Curve (AUC) scores of genes regulated by TFs in CMs. **d.** Feature pattern curves of CMs visualized by Mfuzz depicting dynamic AUC score

changes of TFs across all 6 age groups AG1-6. Left: LV. Right: RV. **e.** Network visualization of up- (left) and down-regulated (right) core regulatory TFs across lifespan, matching the results in **(d)**. Top: Set of continuously up-regulated TFs in LV represented by Cluster 6, and the set continuously down-regulated represented by Cluster 4. Bottom: Set of continuously up-regulated TFs in RV represented by Cluster 6, and the set continuously down-regulated represented by Cluster 9. Color keys from light to dark indicated the |Log2 (AG5 AUC score/AG6 AUC score) | of these TFs from low to high.

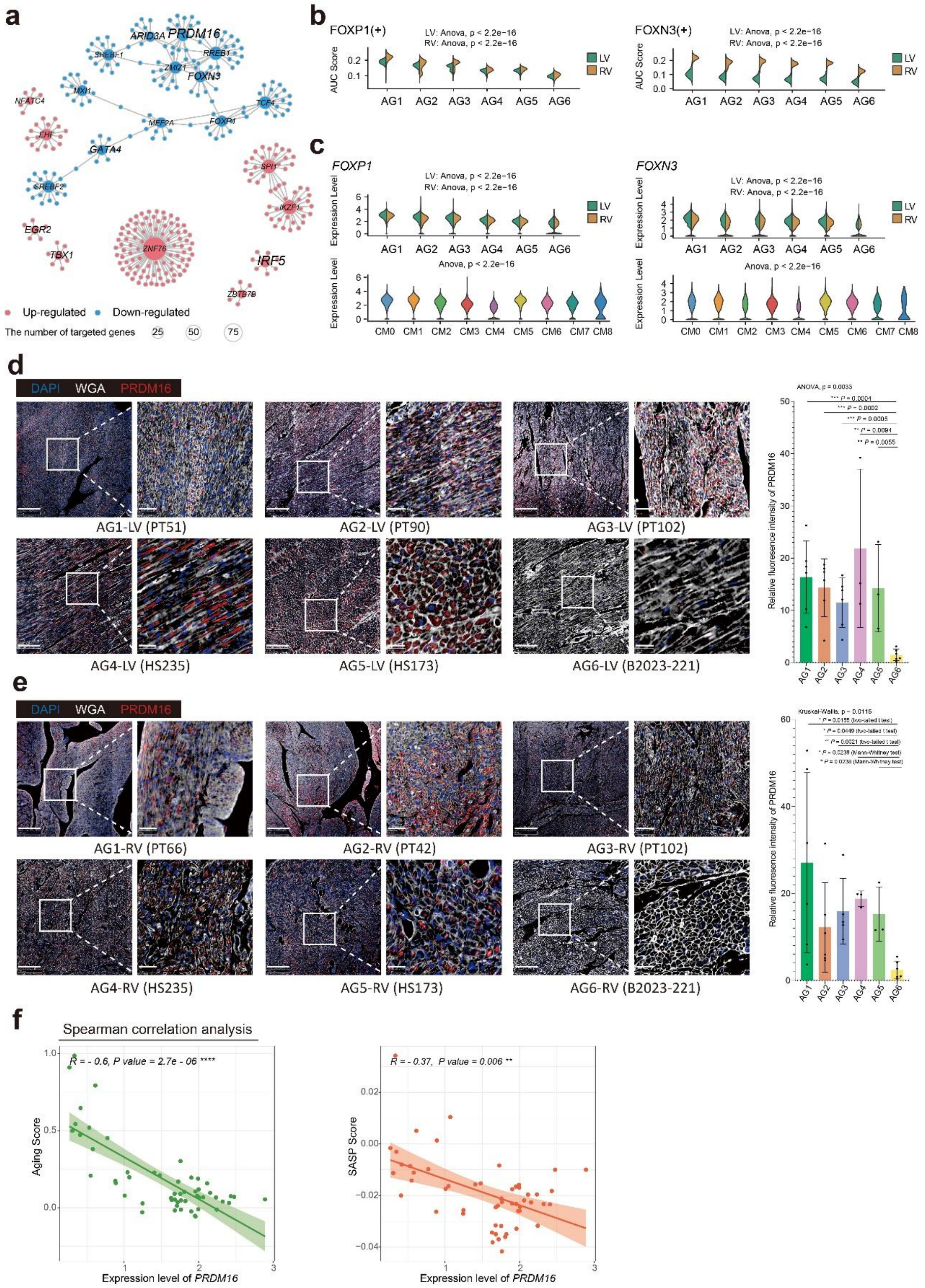

Fig. s12 | Profiling of the transcription factors (TFs) signatures and *PRDM16* in cardiomyocytes (CM) across lifespan. a. Regulatory networks visualizing the key transcriptional regulators in CM across lifespan. Smaller nodes represent target genes and larger nodes represent TFs, matched the results in **Fig. 3A**. The node size of TFs positively correlated with the number of target genes it regulated. Red nodes, up-regulated; blue nodes, down-regulated. **b.** Violin plots showing the AUC score of *FOXP1*

(left) and *FOXN3* (right) in CMs between groups. **c.** Violin plots showing the expression of *FOXP1* (left) and *FOXN3* (right) in CM in groups (top) and cell states (bottom). **d.** Representative images of PRDM16 immunofluorescence staining between 6 groups in left ventricle (left). Scale bars, 250 μm and 50 μm (Magnification). Quantification and statistical analysis of image results (right), by ANOVA test. Two-tailed t tests were used to compare AG6 with other groups. Data were shown as Mean ± SD. All statistically significant inter-group comparisons were annotated, not annotated meant no significance. **e.** Representative images of PRDM16 staining between 6 groups in right ventricle (left). Scale bars, 250 μm and 50 μm (Magnification). Quantification and statistical analysis of image results (right), by Kruskal-Wallis test. Two-tailed t tests or Mann-Whitney tests were used to compare AG6 with other groups. Data were shown as Mean ± SD. All statistically significant inter-group comparisons were annotated, not annotated meant no significance. **f.** Correlations between the *PRDM16* expression levels and aging scores (left) / SASP scores (right) in CMs. Aging scores and SASP scores matched the results in **Fig. 2E**. Each scatter represented the data of an individual sample. Spearman correlation analysis was used to perform the tests. * P value < 0.05, ** P value < 0.01, **** P value < 0.0001, no significant difference P value > 0.05.

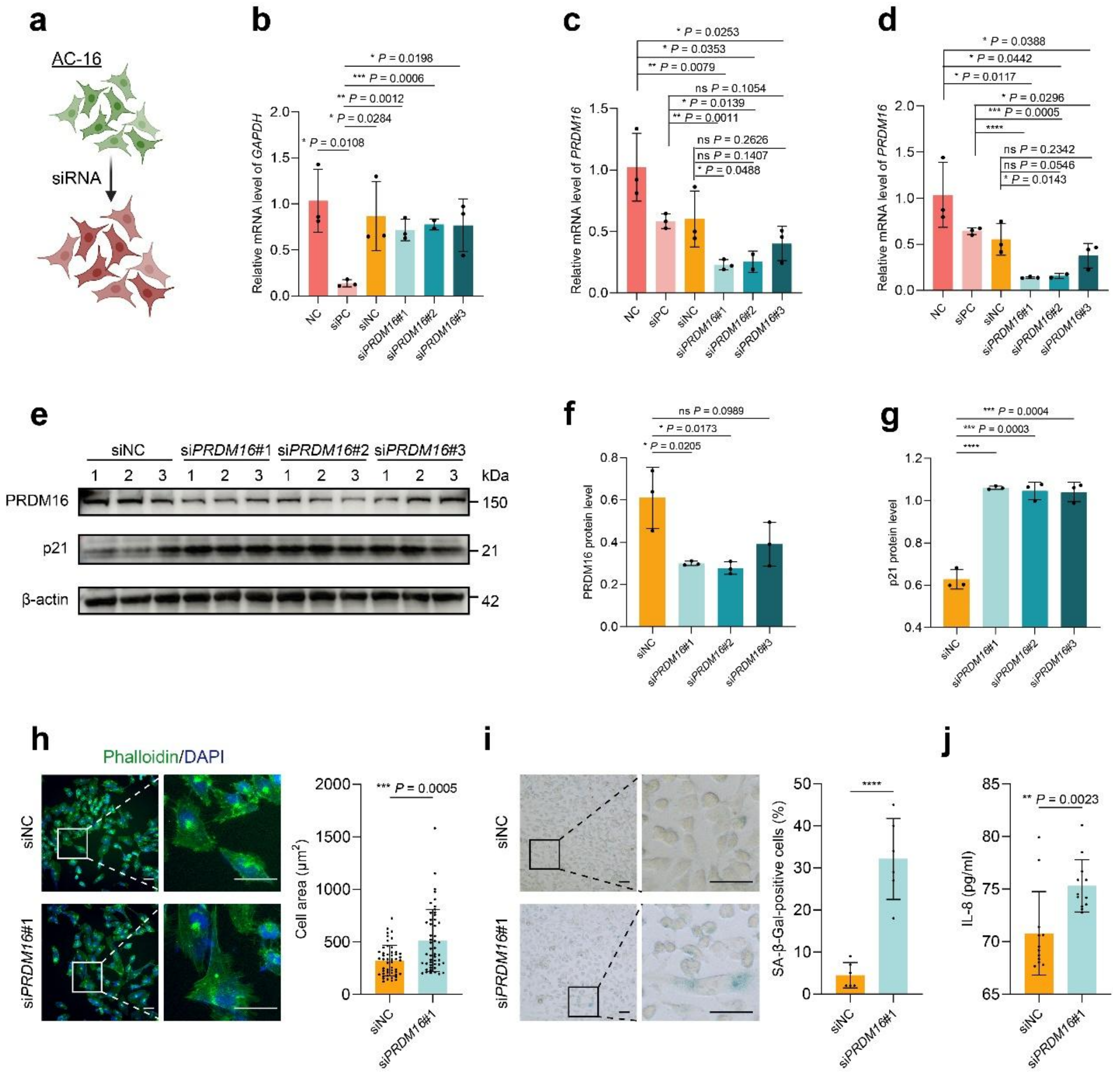

Fig. s13 | Down-regulated *PRDM16* induced cellular senescence in AC-16. a. Schematic diagram of generating *PRDM16* knock-down (KD) in AC-16 cells using siRNA system. Created in BioRender. Jia, H. (2026) https://BioRender.com/iinvojk. **b.** RT-qPCR detecting silent efficacy of si-positive control (PC) (siRNA-*GAPDH*) in AC-16 cells. Two-tailed t tests were used to compare group siPC with other groups. n=3 per group, biological replicates. Data were shown as Mean ± SD. **c, d.** RT-qPCR detecting silent efficacy of si*PRDM16* in AC-16 by using *PRDM16*-primer-1 **(c)** and *PRDM16*-primer-2 **(d)**. n=3 per group, biological replicates. Two-tailed t tests were used to compare groups si*PRDM16* with other groups. Data were shown as Mean ± SD. **e.** Western blotting of PRDM16 and p21. β-actin was used as the control. n=3 per group, biological replicates. **f, g.** Semi-quantitative data of PRDM16 **(f)** and p21 **(g)** protein level, matched the result in **(e)**. Two-tailed t tests were used to compare group siNC with other groups. Data were shown as Mean ± SD. **h.** Representative fluorescence images of phalloidin staining in AC-16 (left). Scale bars, 50 μm. Semi-quantitative data of cell area (right), n=50 per group, biological replicates, Mann-Whitney test. Data were shown as Mean ± SD. **i.** Representative images of SA-β-Gal staining in AC-16 (left). Scale bars, 50 μm. The percentage of SA-β-Gal-positive cells (right), n=6 per group, biological replicates, two-tailed t test. Data were shown as Mean ± SD. **j.** ELISA results of IL-8 concentration in the culture supernatant, n=12 per group, biological replicates, Mann-

Whitney test. Data were shown as Mean ± SD. * *P* value < 0.05, ** *P* value < 0.01, *** *P* value < 0.001, **** *P* value < 0.0001, no significant difference *P* value > 0.05.

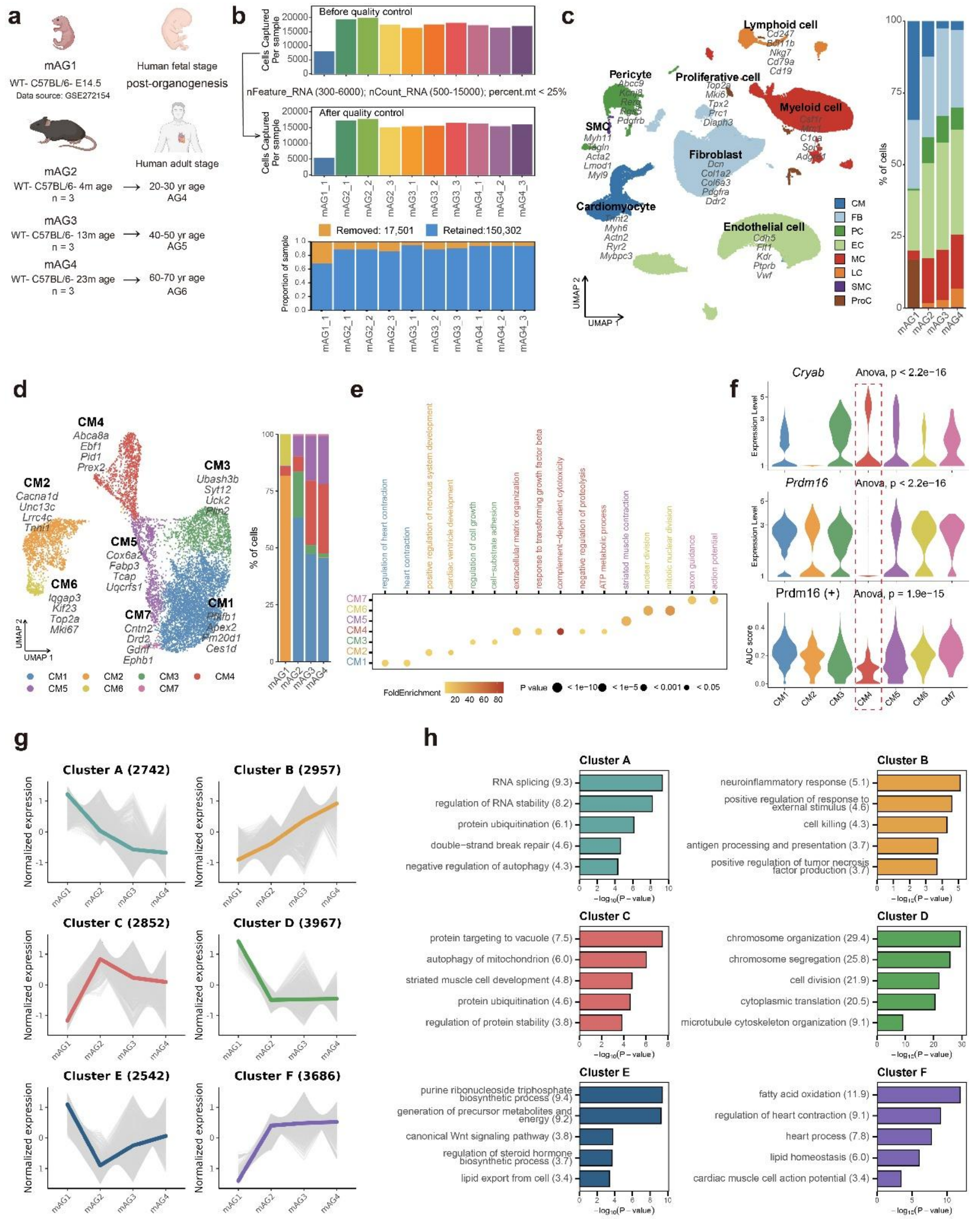

Fig. s14 | Single-nucleus atlas of mouse non-failing fetal and adult hearts and its comparison with the human heart atlas. a. Schematic diagram of mouse sample inclusion. Created in BioRender. Jia, H. (2026) https://BioRender.com/h0xu32t. **b.** The number of nuclei per sample detected before (top) and after (middle) quality control. Proportion of retained and removed nuclei per sample (n=10) after quality control (bottom). **c.** Left: Uniform manifold approximation and projection (UMAP) plots embedding of 150,302 single-nuclei delineated 8 major cardiac cell types. SMC, smooth muscle cell. Right: Proportion of major cell types across mAG1-4. **d.** Left: UMAP embedding depicting 7 CM cell states. Right: Proportion of CM cell states across mAG1-4. **e.** Dot plot showing the enrichment of Gene Ontology (GO) biological processes in each CM cell state. **f.** Violin plots visualizing the expression level of *Cryab* (top) and *Prdm16*

(middle) and the AUC score of *Prdm16* (bottom) across all CM cell states. **g.** Feature pattern curves by Mfuzz depicting dynamic gene expression changes across all 4 age groups in CM. **h.** Six gene clusters in (based on dynamic gene expression changes in **(g)**) were identified using GO analysis. Numbers in parentheses in **g** represented the number of genes included in analyses. Numbers in parentheses in **h** represented the GO term gene ratio.

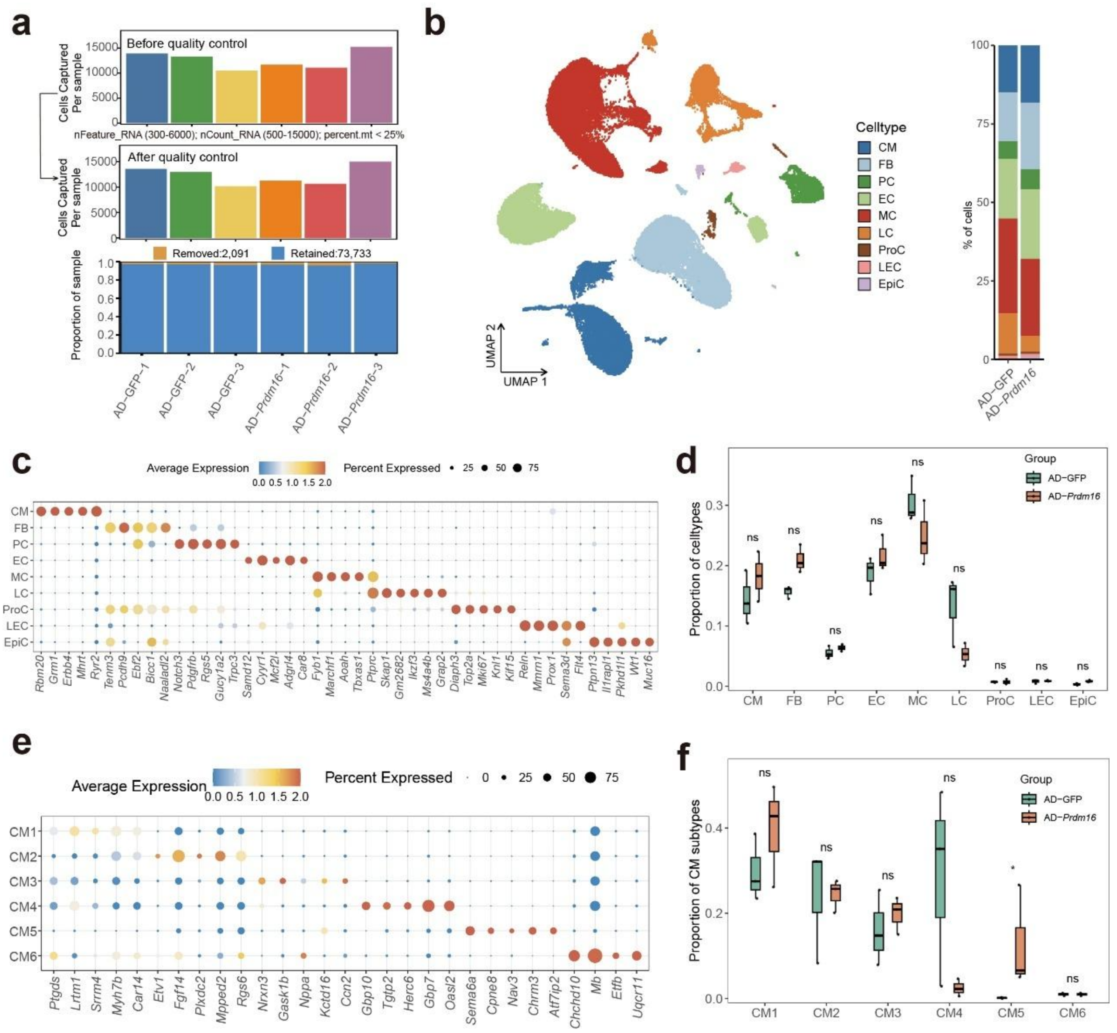

Fig. s15 | Additional Transcriptional features of *Prdm16* overexpression in the aged mouse hearts. a. The number of nuclei per sample detected before (top) and after (middle) quality control. Proportion of retained and removed nuclei per sample (n=6) after quality control (bottom). **b.** Left: UMAP plots embedding of 73,733 single-nuclei delineated 9 major cell types. Right: Proportion of major cell types between the two groups. CM, cardiomyocyte; EC, endothelial cell; LEC, lymphatic endothelial cell; FB, fibroblast; PC, pericyte; MC, myeloid cell; LC, lymphoid cell; ProC, proliferative cell; EpiC, epicardial cell. **c.** Dot plots visualizing the marker genes of each major cell type. **d.** Proportion of each major cell type between the two groups. Center line, median; box limits, upper and lower quartiles; whiskers, 1.5x interquartile range, two-tailed t tests. **e.** Dot plots visualizing the marker genes of each CM cell state. **f.** Proportion of each CM cell state between the two groups. Center line, median; box limits, upper and lower quartiles; whiskers, 1.5x interquartile range, two-tailed t tests. **P* value < 0.05; ns, no significant difference, *P* value > 0.05.

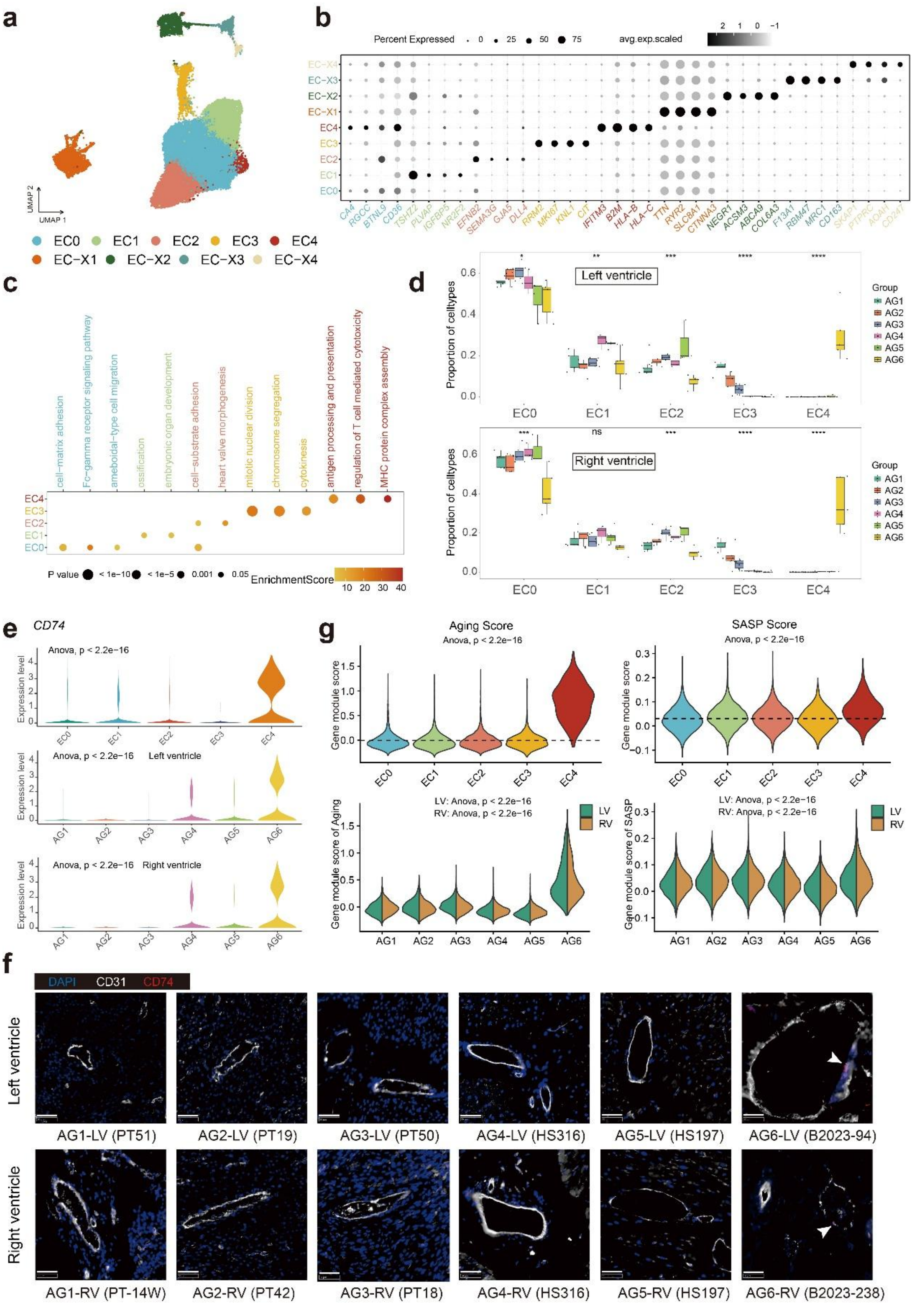

Fig. s16 | Characterization of ECs. a. UMAP embedding depicting 5 EC cell states. **b.** Dot plots showing the marker genes of each EC cell state. **c.** Dot plot showing enrichment of Gene Ontology (GO) biological processes in each EC cell state. **d.** Proportion of each EC cell state stratified by age groups AG1-6. Center line, median; box limits, upper and lower quartiles; whiskers, 1.5x interquartile range. Statistically credible changes in cell states were tested with ANOVA. **e.** Violin plots depicting *CD74* expression levels across

all EC cell states (top) and age groups AG1-6 in LV (middle) and RV (bottom). **f.** *In situ* Immunostaining for CD74, co-stained with DAPI and CD31. Scale bars, 50 μm. Arrowheads showed co-expression of CD31 and CD74. **g.** Violin plots showing increased aging and SASP scores in EC4 and AG6, when comparing each EC cell state (top) and age group (bottom). **P* value < 0.05, ** *P* value < 0.01, *** *P* value < 0.001, and **** *P* value < 0.0001; ns, no significant difference, *P* value > 0.05.

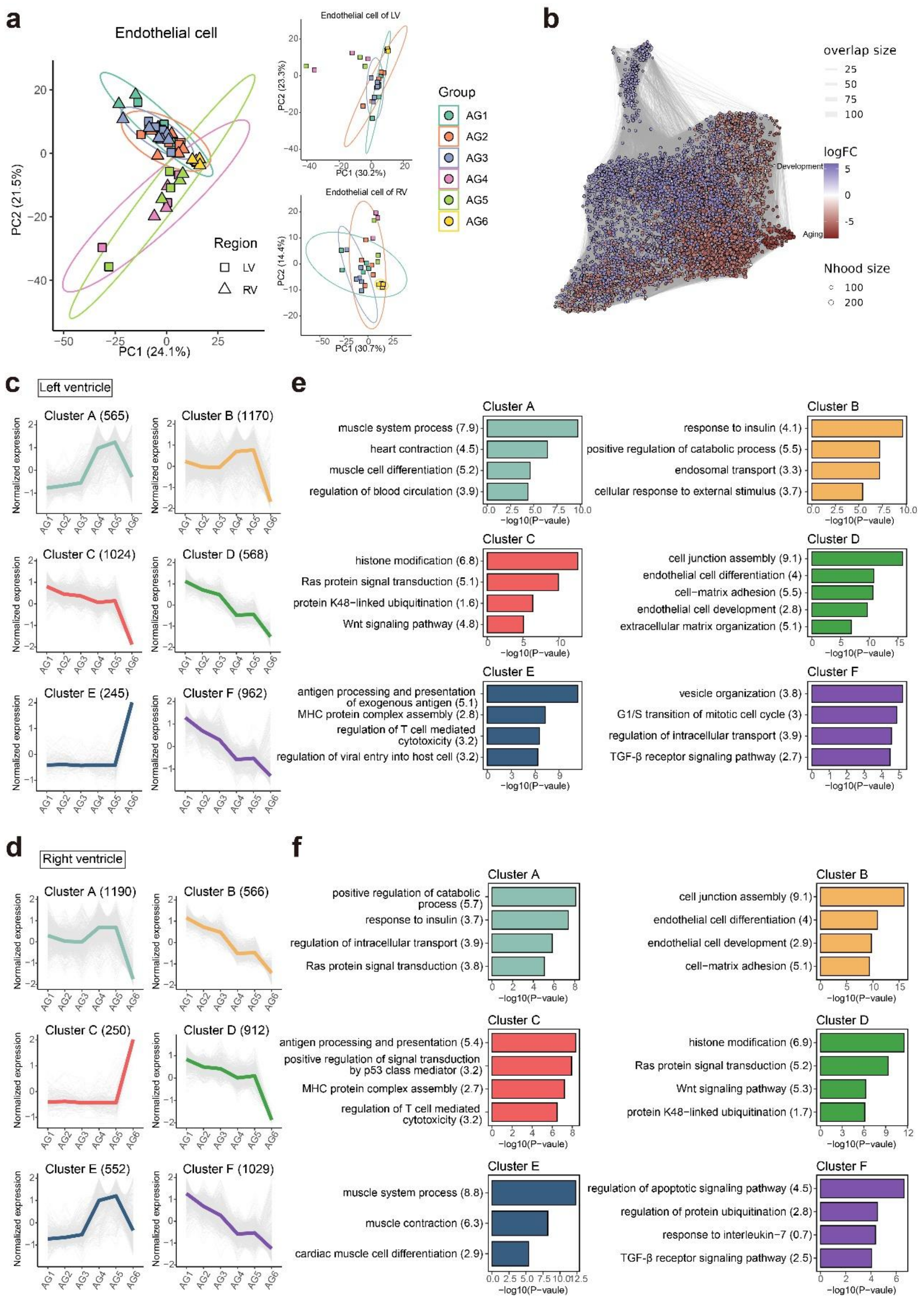

Fig. s17 | snRNA-seq showing the unique transcriptional signature of ECs across lifespan. a. PCA plots of pseudo-bulk RNA-seq analyses including all EC nuclei. **b.** Embedding of Milo *k*-nearest neighbour differential abundance testing in EC nuclei. All nodes represented neighbourhoods. The layout of nodes was determined by UMAP embedding, as shown in **Fig. s16a.** Feature pattern curves generated by Mfuzz depicting dynamic gene expression changes across all 6 age groups in EC of LV **(c)** and RV **(d)**. **e.**

Six gene clusters in (based on dynamic gene expression changes in **(c)**) were identified using GO analysis. **f.** Six gene clusters in (based on dynamic gene expression changes in **(d)**) were identified using GO analysis. Numbers in parentheses in **c** and **d** represented the number of genes. Numbers in parentheses in **e** and **f** represented the GO term gene ratio.

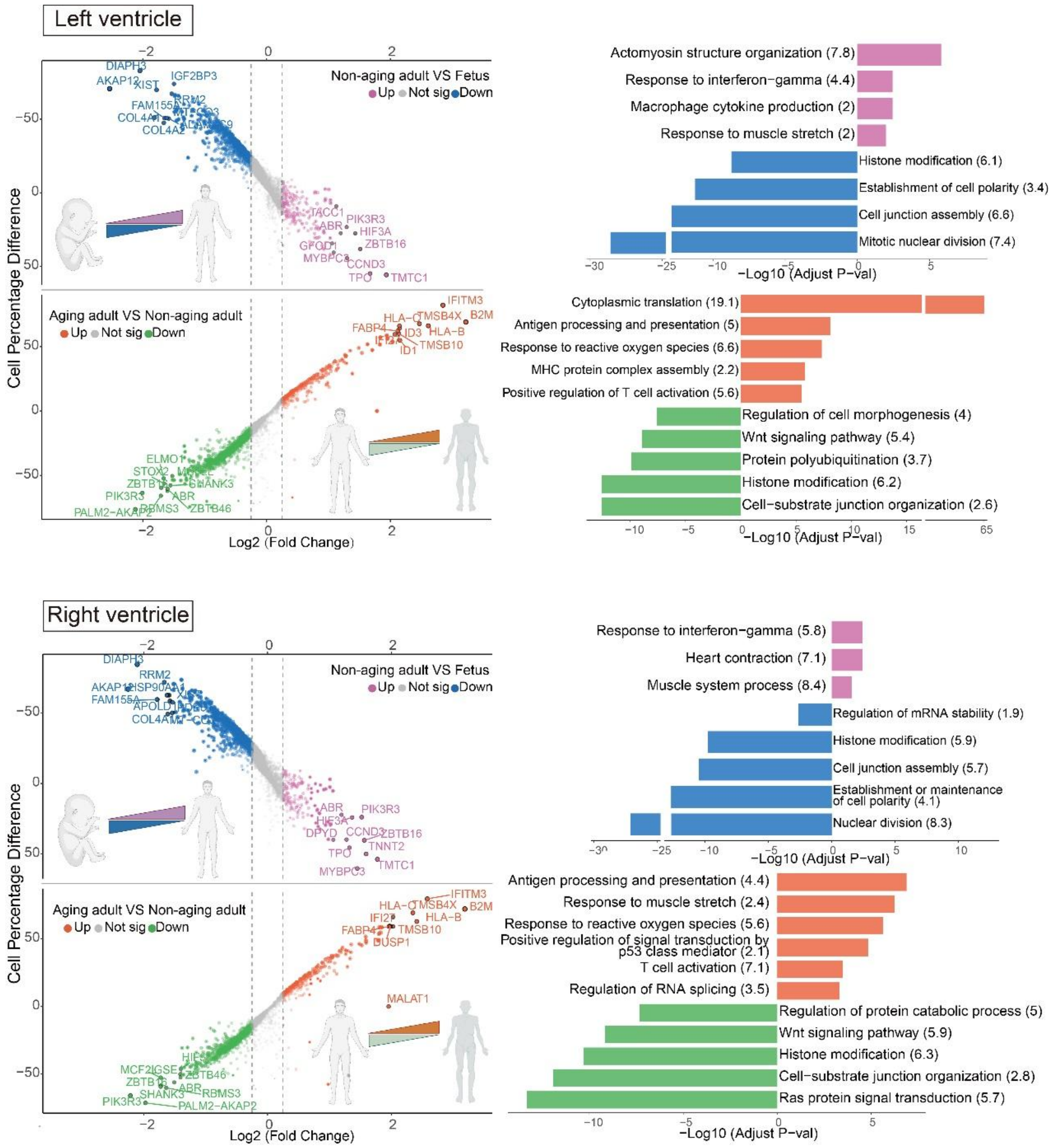

Fig. s18 | Pairwise comparisons of ECs across lifespan. Volcano plots depicting the differentially expressed genes (DEGs) signatures between the dominant cell states of fetus (AG1, AG2, AG3 - EC3) and adolescent/adult (AG4, AG5 - EC0, EC1, EC2) and elderly (AG6 - EC4). Opaque dots represented DEGs with FDR < 0.01 and |Log2 (fold change) | > 0.25. GO terms showing the functional enrichment of DEGs, colored according to the designations in the volcano plots. Numbers in parentheses represented the gene ratio for the GO terms. Created in BioRender. Jia, H. (2026) https://BioRender.com/2my5i2t.

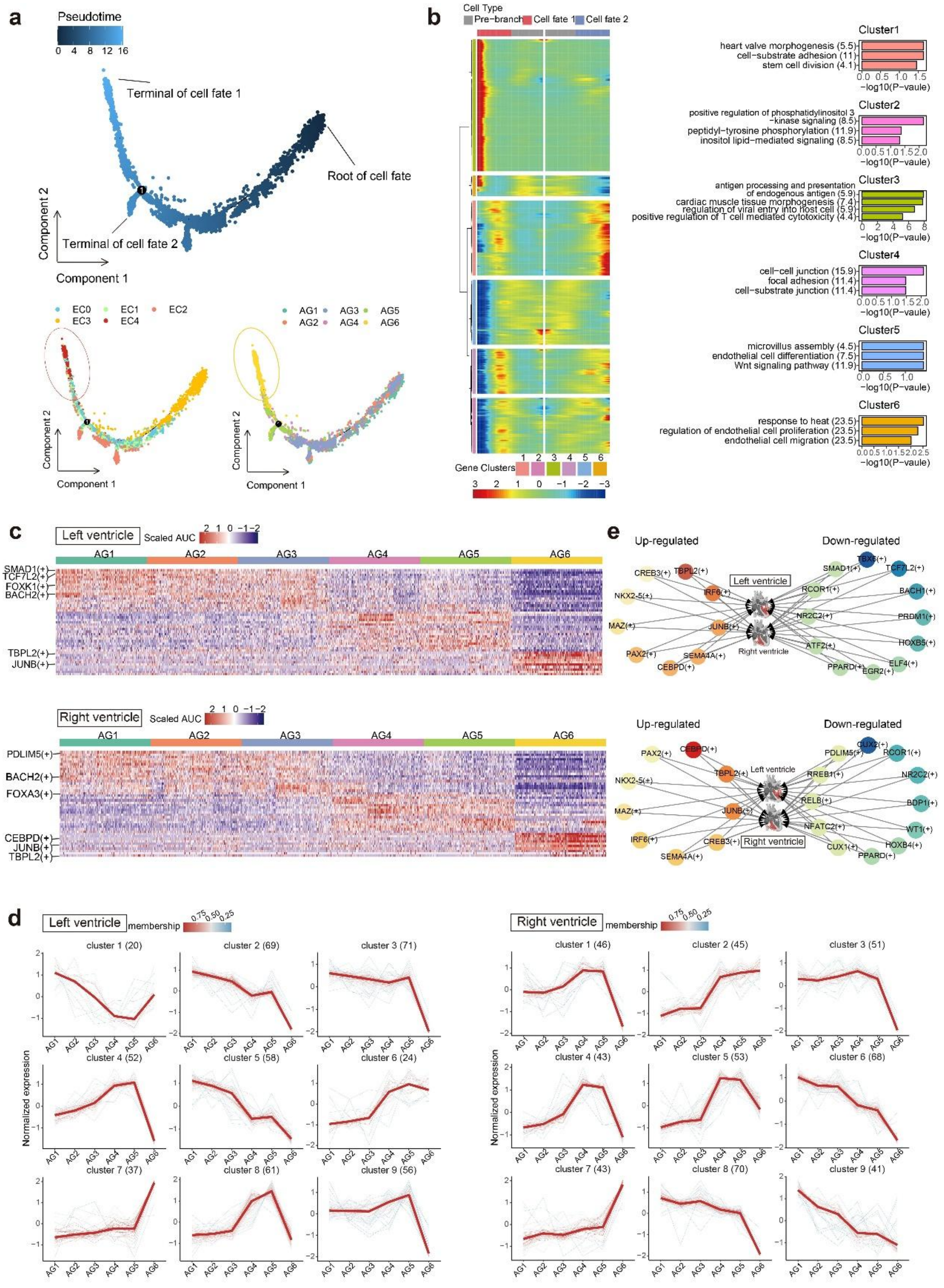

Fig. s19 | Pseudotime trajectory and transcription factors (TFs) signatures of ECs. **a.** Pseudotime trajectory of ECs. Circles represented the regions where EC4 and AG6 groups were located. **b.** Heatmap showing differentially expressed genes (DEGs) alone with the pseudotime as in **(a)**, cataloged into six gene module clusters (left). Six gene clusters were identified with GO analysis. (right). Numbers in parentheses represented the gene ratio for the GO terms. **c.** Heatmap depicting the Area Under Curve (AUC) scores for the expression of genes regulated by TFs in ECs. **d.** Feature pattern curves of ECs

visualized by the Mfuzz depicting dynamic AUC score changes of TFs across the 6 age groups AG1-6. Left:LV; Right: RV. **e.** Network visualization of up- (left) and down-regulated (right) core regulatory TFs across lifespan, matching the results in **(d)**. The set of continuously up-regulated TFs in LV was Cluster 7, and the sets continuously down-regulated were Cluster 2 and Cluster 5 (Top). The sets of continuously up-regulated TFs in RV were Cluster 2 and Cluster 7, and the sets continuously down-regulated were Cluster 6, 8, and 9 (bottom). Color keys from light to dark indicating the |Log2 (AG5 AUC score/AG6 AUC score) | of these TFs from low to high.

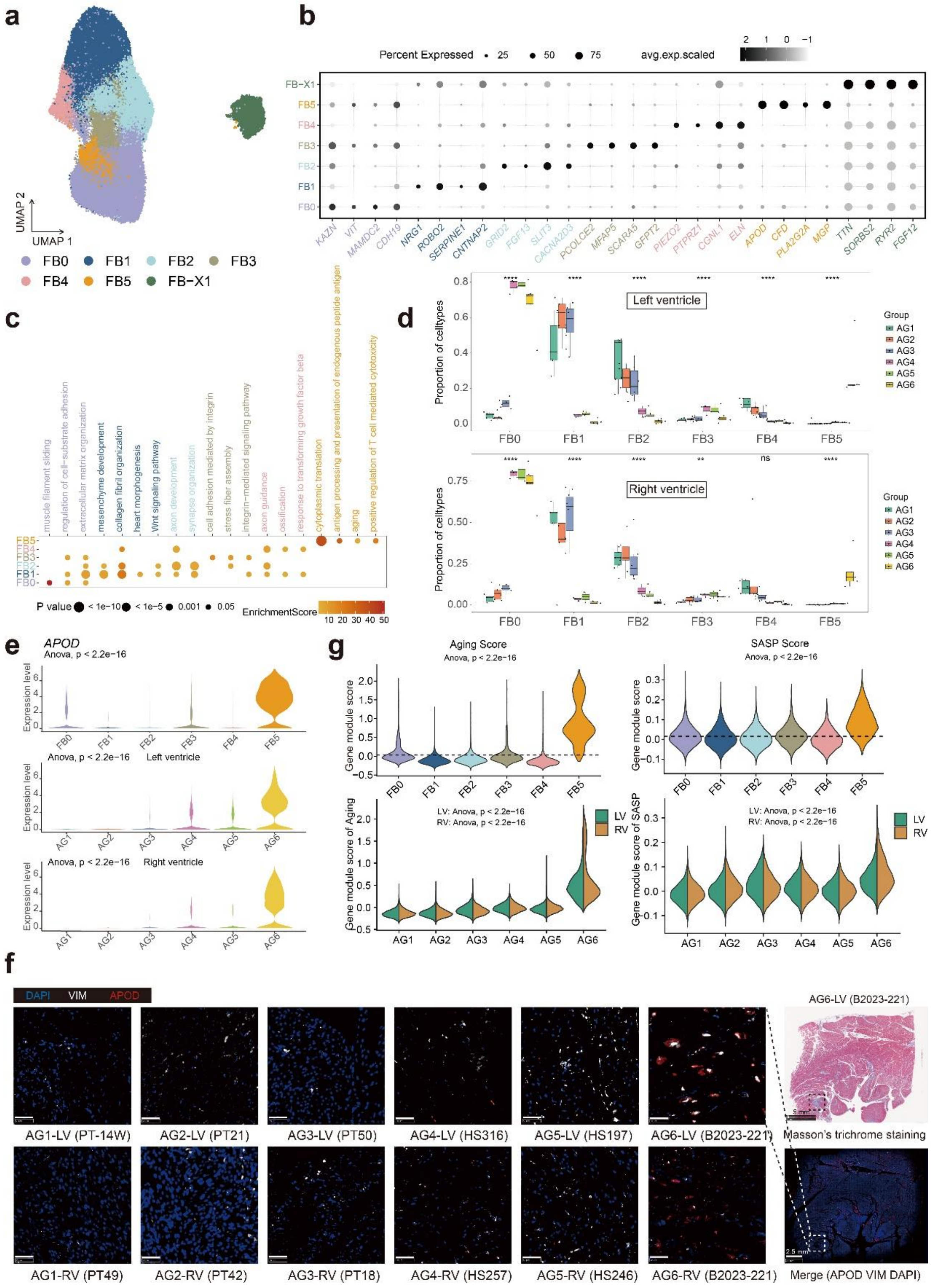

Fig. s20 | Characterization of FBs. a. UMAP embedding depicting 6 FB cell state. **b.** Dot plots showing the marker genes of each FB cell state. **c.** Dot plot showing enrichment of Gene Ontology (GO) biological processes in each FB cell state. **d.** Proportion of each FB cell state stratified by age groups AG1-6. Center line, median; box limits, upper and lower quartiles; whiskers, 1.5x interquartile range. Statistically credible changes in cell states were tested with ANOVA. **e.** Violin plots depicting the expression levels of *APOD*

for FB. **f.** *In situ* immunostaining for APOD, co-stained with DAPI and Vimentin (VIM). Scale bars, 50 μm. **g.** Violin plots showing increased aging and SASP scores of FB5 and AG6 by inter-subcluster comparison (top) or inter-group comparison (bottom). ** *P* value < 0.01 and **** *P* value < 0.0001; ns, no significant difference, *P* value > 0.05.

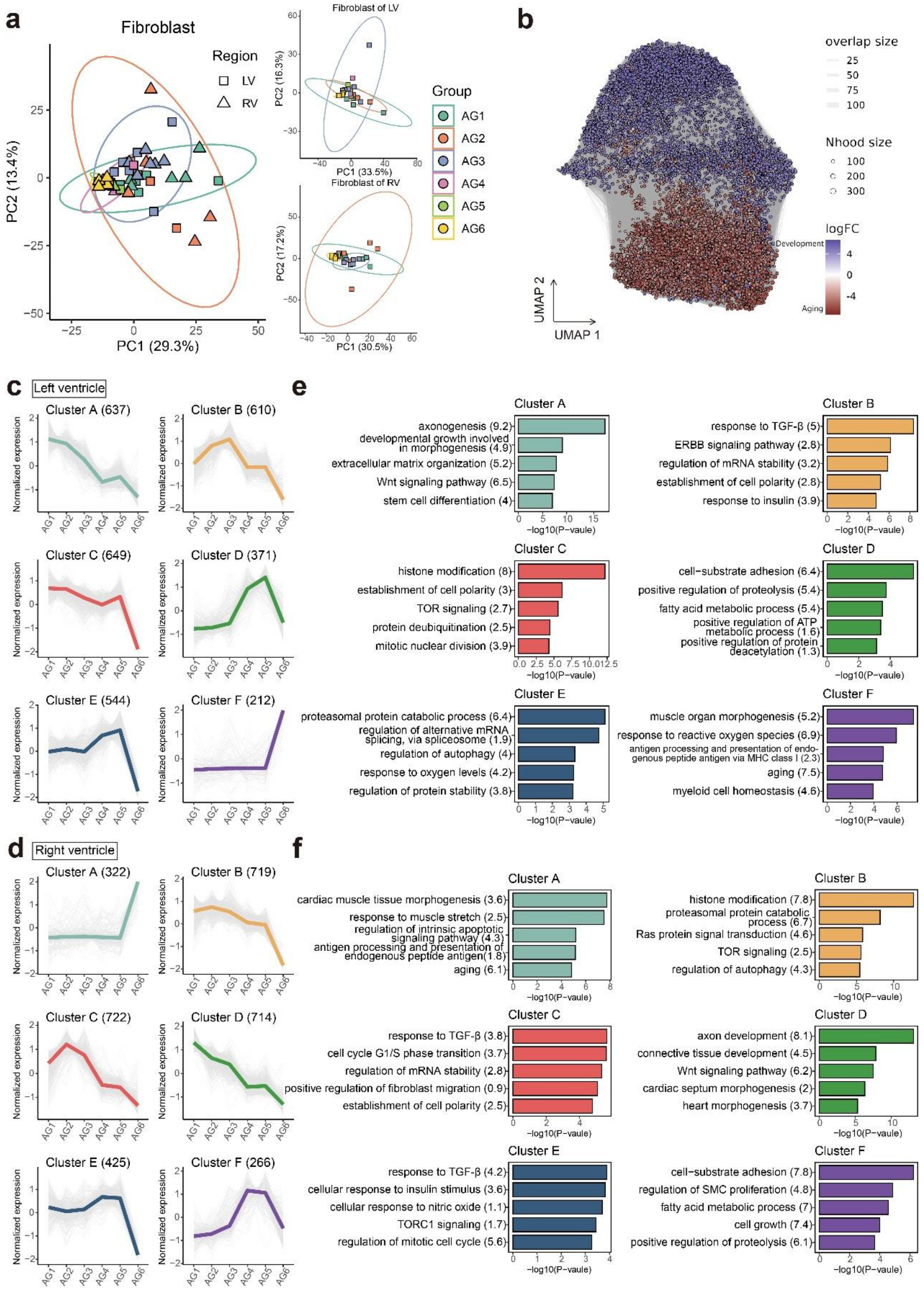

Fig. s21 | snRNA-seq showing the unique transcriptional signature of FBs across lifespan. a. PCA plots of pseudo-bulk RNA-seq analysis including FB nuclei. **b**. Embedding of Milo *k*-nearest neighbour differential abundance testing in FB nuclei. All nodes represented neighbourhoods. The layout of nodes was determined by UMAP embedding, as shown in **Fig. s20a.** Feature pattern curves generated by Mfuzz depicting dynamic gene expression changes across all 6 age groups AG1-6 in FBs of LV **(c)** and RV

(d). **e.** Six gene clusters in (based on dynamic gene expression changes in **(c)**) were identified with GO analysis. **f.** Six gene clusters in (based on dynamic gene expression changes in **(d)**) were identified with GO analysis. Numbers in parentheses in **c** and **d** represented the number of genes. Numbers in parentheses in **e** and **f** represented the GO term gene ratio.

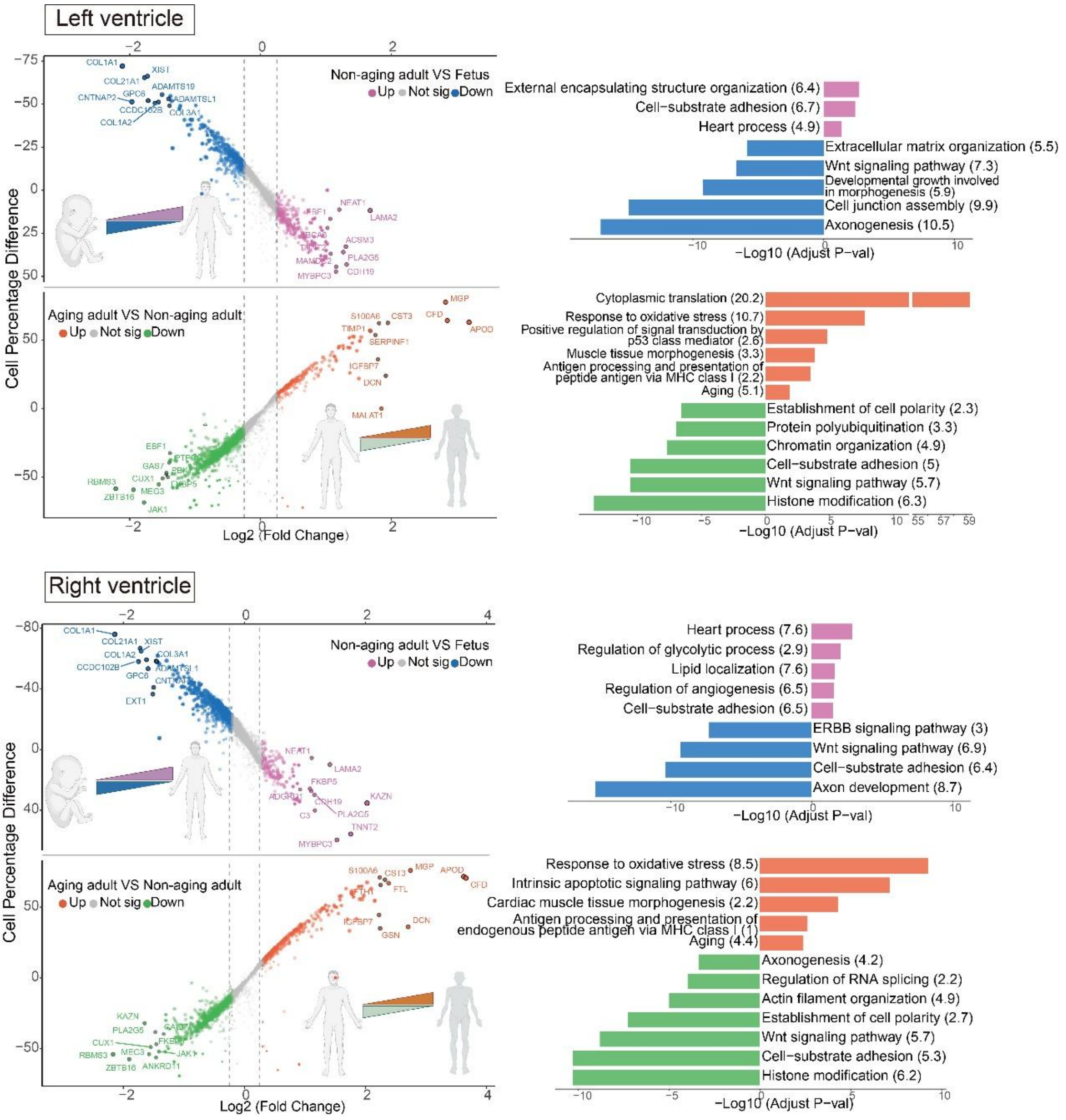

Fig. s22 | Pairwise comparisons of FBs across lifespan. Volcano plots depicting the differentially expressed genes (DEGs) signature between the dominant cell states of fetus (AG1, AG2, AG3 - FB1, FB2, FB4) and adolescent/adult (AG4, AG5 - FB0, FB3) and elderly (AG6 - FB5). Opaque dots represented DEGs with FDR < 0.01 and |Log2 (fold change) | > 0.25. GO terms showed the functional enrichment of DEGs, colored according to the designations in the volcano plots. Numbers in parentheses represented the gene ratio for the GO terms. Created in BioRender. Jia, H. (2026) https://BioRender.com/7bp9yao.

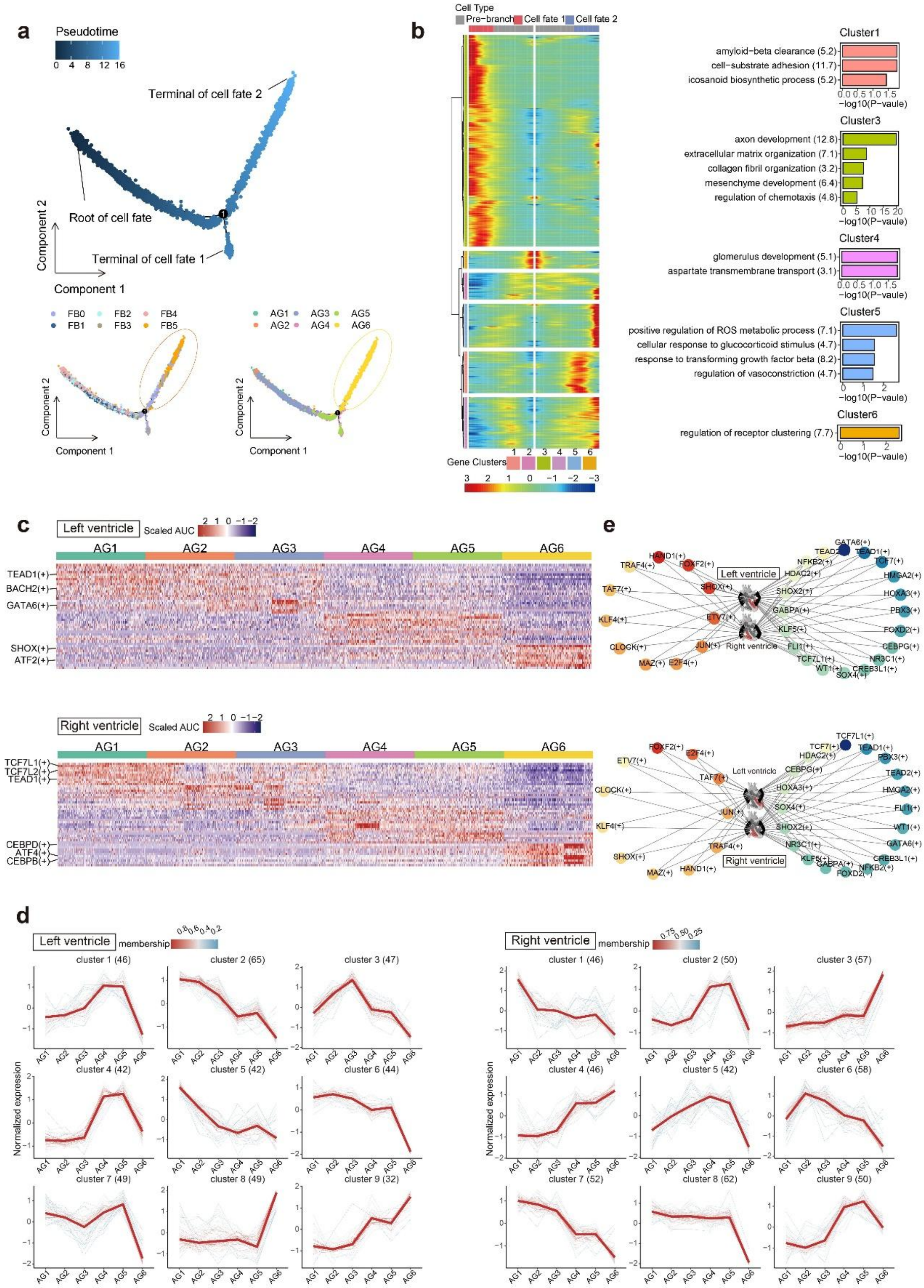

Fig. s23 | Pseudotime trajectory and transcription factors (TFs) signatures of FBs. **a.** Pseudotime trajectory of FBs. Circles were the regions where FB5 and AG6 groups were located. **b.** Heatmap showing differentially expressed genes (DEGs) alone with the pseudotime as in **(a)**, cataloged into six gene module clusters (left). Six gene clusters were identified with GO analysis (right). Numbers in parentheses represented the gene ratio for the GO terms. **c.** Heatmap depicting the Area Under Curve (AUC) scores for the

expression of genes regulated by TFs in FBs. **d.** Feature pattern curves of FBs visualized using Mfuzz depicting dynamic AUC score changes of TFs across the 6 age groups AG1-6. Left: LV. Right: RV. **e.** Network visualization of up- (left) and down-regulated (right) core regulatory TFs across lifespan, matching the results in **(d)**. The set of continuously up-regulated TFs in LV was Cluster 9, and the set continuously down-regulated was Cluster 2 (Top). The sets of continuously up-regulated TFs in RV were Cluster 3 and Cluster 4, and the set continuously down-regulated were Cluster 1 and Cluster 7 (bottom). Color keys from light to dark indicated the |Log2 (AG5 AUC score/AG6 AUC score) | of these TFs from low to high.

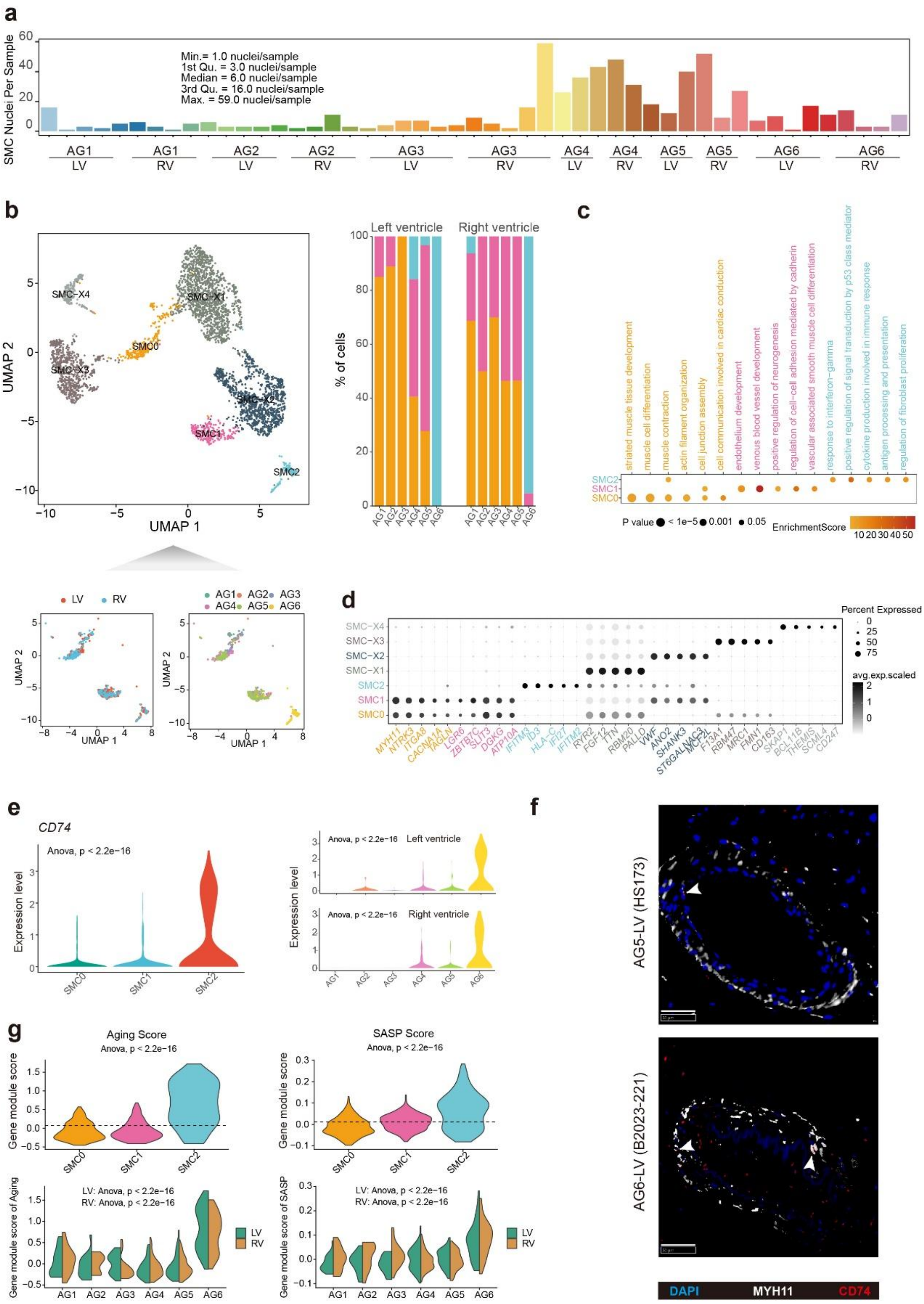

Fig. s24 | Characterization of SMCs. a. SMC nuclei number per sample. **b.** UMAP plot depicting the subclustering and cell states annotation of SMC (left, n = 617 excluding SMC-X1, X2, X3, and X4). UMAPs of SMC nuclei were colored by anatomical sites LV vs. RV (bottom-left) and the age group assignment AG1-6 (bottom-right). Distributions of SMC cell states in 6 age groups AG1-6 (right). **c.** Dot plot depicting Gene Ontology (GO) biological processes of each SMC cell state. **d.** Dot plots showing the marker genes of

each SMC cell state. **e.** Violin plots depicting CD74 expression levels in SMCs. **f.** Representative *in situ* immunostaining of CD74 in SMCs, co-stained with DAPI and *MYH11*. Scale bars, 50 μm**.** Arrowheads showed co-expression of *MYH11* and *CD74*. **g.** Violin plots showing age and SASP scores of SMCs stratified by cell states, age group assignment and cardiac chambers.

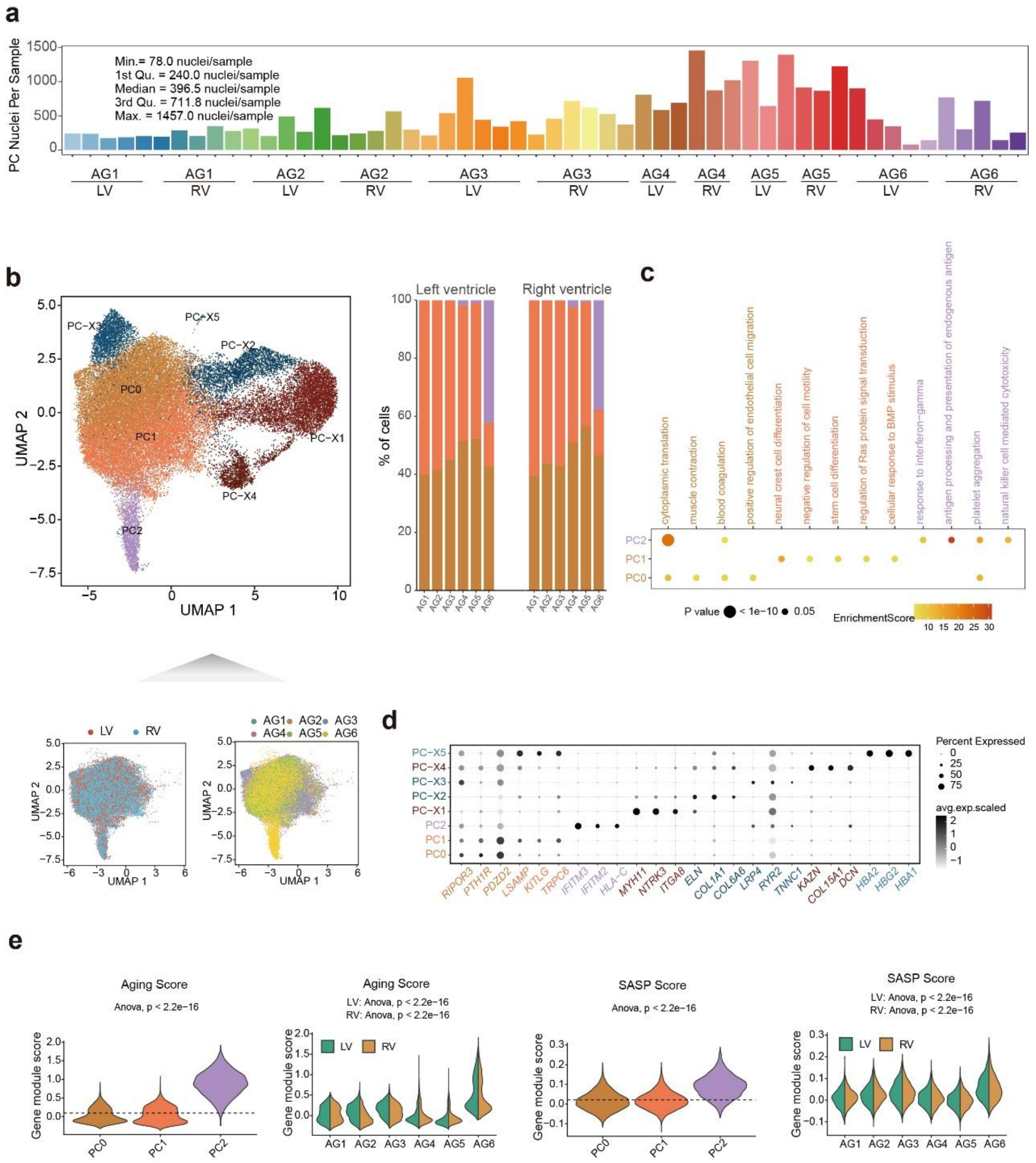

Fig. s25 | Characterization of PCs. a. PC nuclei number per sample. **b.** UMAP plot depicting the subclustering and cell states annotation of PC (left, n = 27,646, excluding PC-X1, X2, X3, X4, and X5). UMAPs of PC nuclei were colored by anatomical sites LV vs. RV (bottom-left) and the age group assignment AG1-6 (bottom-right). Proportion of PC cell states across all 6 age groups AG1-6 (right). **c.** Dot plot depicting Gene Ontology (GO) biological processes of each PC cell state. **d.** Dot plots showing the marker genes of each PC cell state. **e.** Violin plots showing age and SASP scores of PCs stratified by cell states, age group assignment and cardiac chambers.

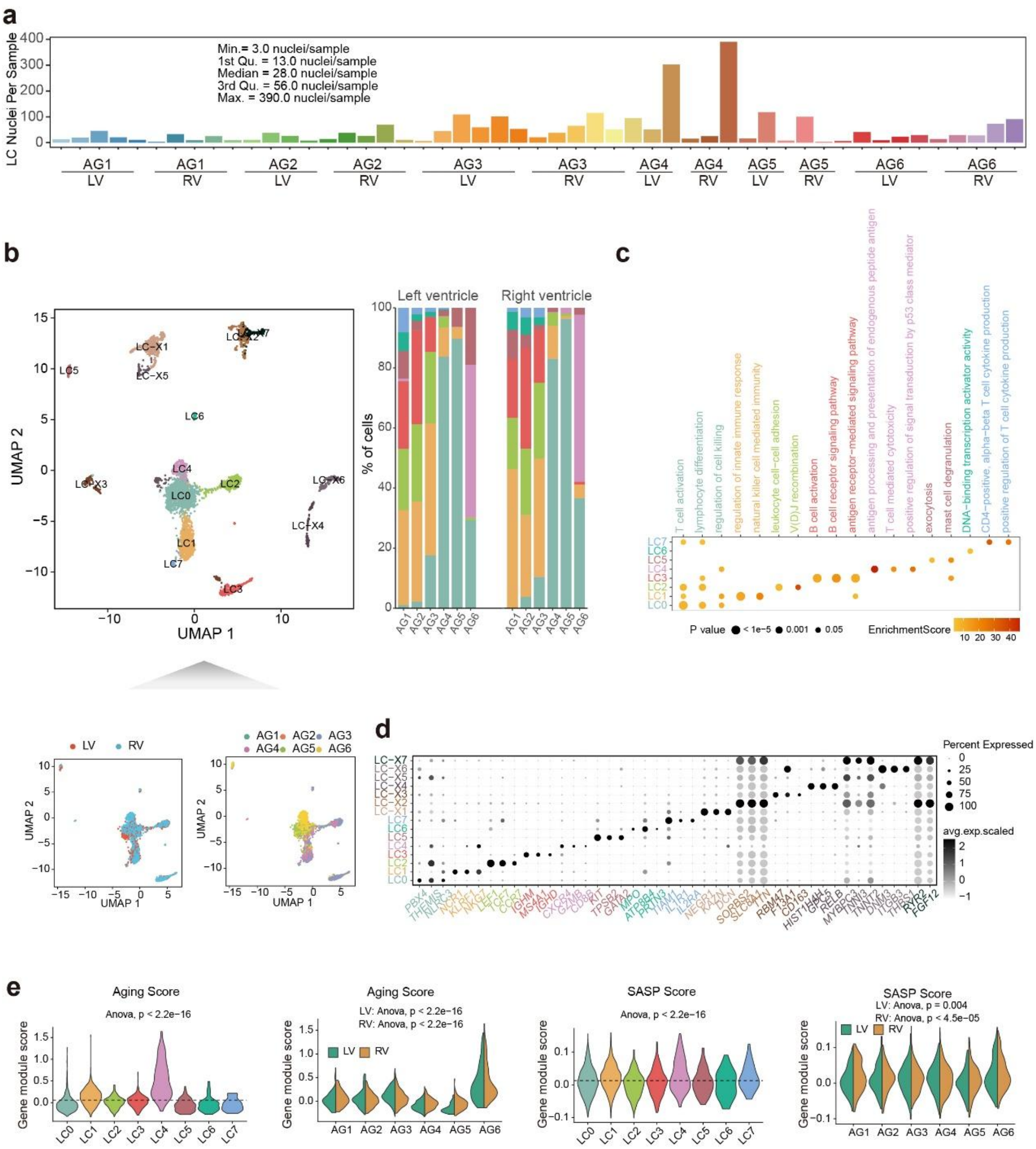

Fig. s26 | Characterization of LCs. a. LC nuclei number per sample. **b.** UMAP plot depicting the subclustering and cell states annotation of the LC lineage (left, n = 2,563 excluding LC-X1, X2, X3, X4, X5, X6, and X7). UMAPs of LC nuclei were colored by anatomical sites LV vs. RV (bottom-left) and the age group assignment AG1-6 (bottom-right). Proportion of LC cell states across all 6 age groups AG1-6 (right). **c.** Dot plot depicting Gene Ontology (GO) biological processes of each LC cell state. **d.** Dot plots showing the marker genes of each LC cell state. **e.** Violin plots showing age and SASP scores of LCs stratified by cell states, age group assignment and cardiac chambers.

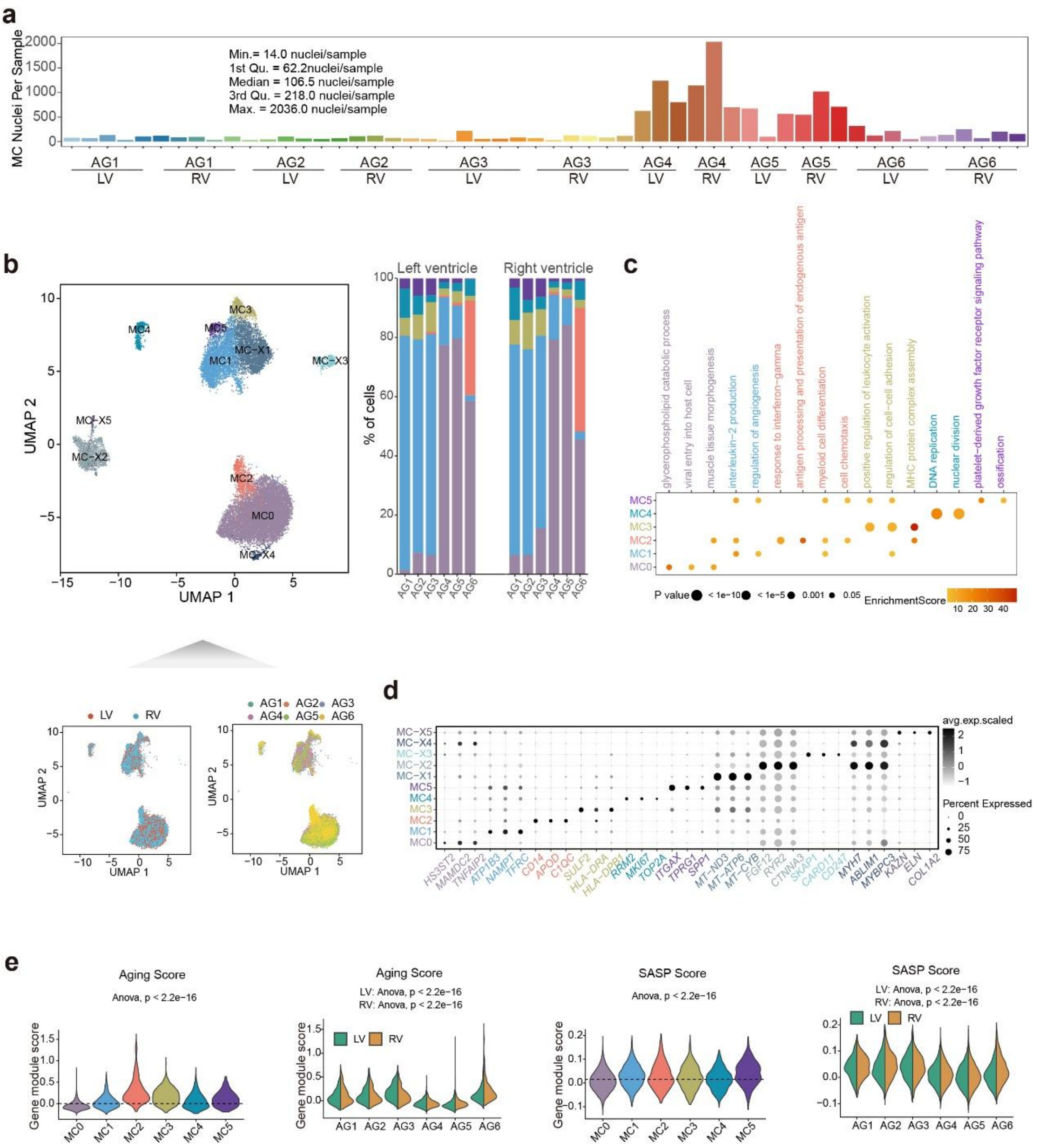

Fig. s27 | Characterization of MCs. a. MC nuclei number per sample. **b.** UMAP plot depicting the subclustering of the MC lineage (left, n = 14,289 excluding MC-X1, X2, X3, X4, and X5). UMAPs of MC nuclei were colored by anatomical sites (bottom-left) and age group assignment AG1-6 (bottom-right). Proportion of MC cell states across all 6 age groups AG1-6 (right). **c.** Dot plot depicting Gene Ontology (GO) biological processes of each MC cell state. **d.** Dot plots depicting the marker genes of each MC cell state. **e.** Violin plots showing age and SASP scores of MCs stratified by cell states, age group assignment and cardiac chambers.

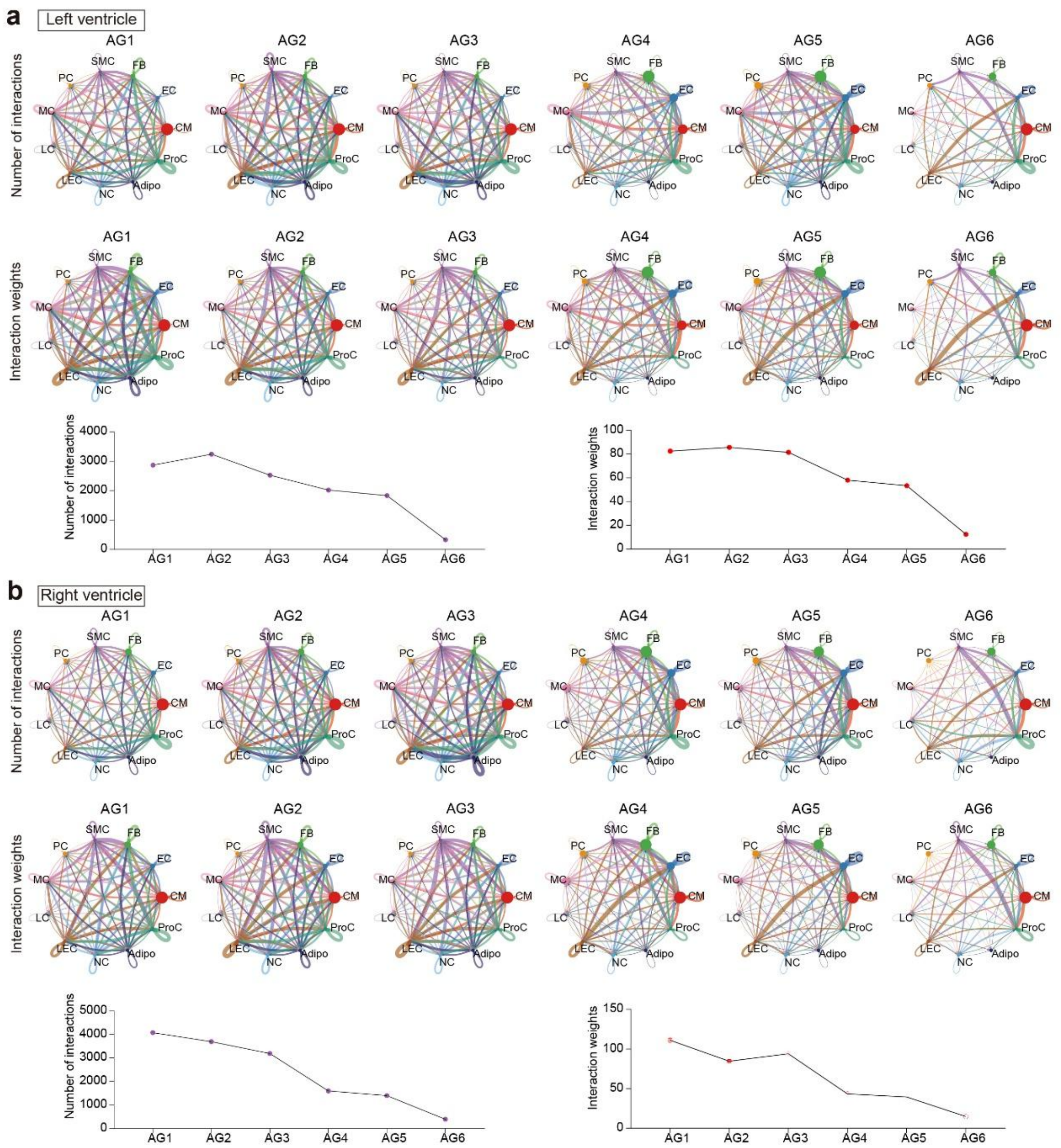

Fig. s28 | Cross-talk between cardiac cells. Circle plots visualizing the number (top) and the weights (middle) of ligand-receptor interactions in-between the cardiac cell types stratified by the age groups AG1-6. The number and the weights of ligand-receptor interactions were quantified (bottom). **A.** Ligand-receptor interaction in LV. **B.** Ligand-receptor interaction in RV.

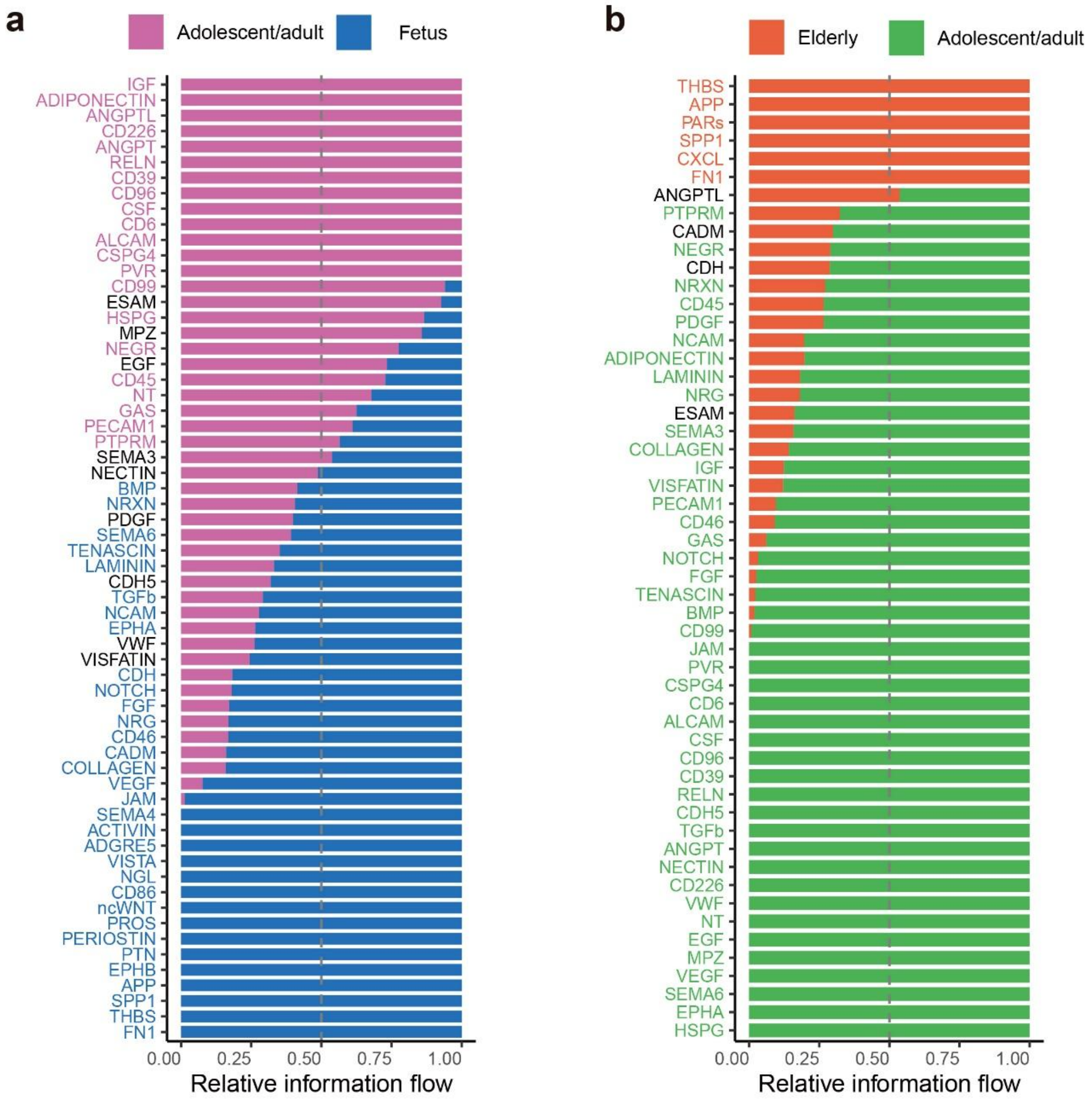

Fig. s29 | Signaling pathways. Signaling pathways were ranked according to their differences in overall information flow within the inferred networks. **a.** Comparison of adolescent/adult groups (AG4 and AG5) and fetus groups (AG1, AG2, and AG3). **b.** Comparison of adolescent/adult groups (AG4 and AG5) and AG6. Data source for analysis matched the results of **Fig. s28**. Signaling pathways with colored fonts were statistically significant, and black fonts meant not significant. Statistically credible changes were tested with two-tailed t tests, and significance was defined by *P* value less than 0.05.

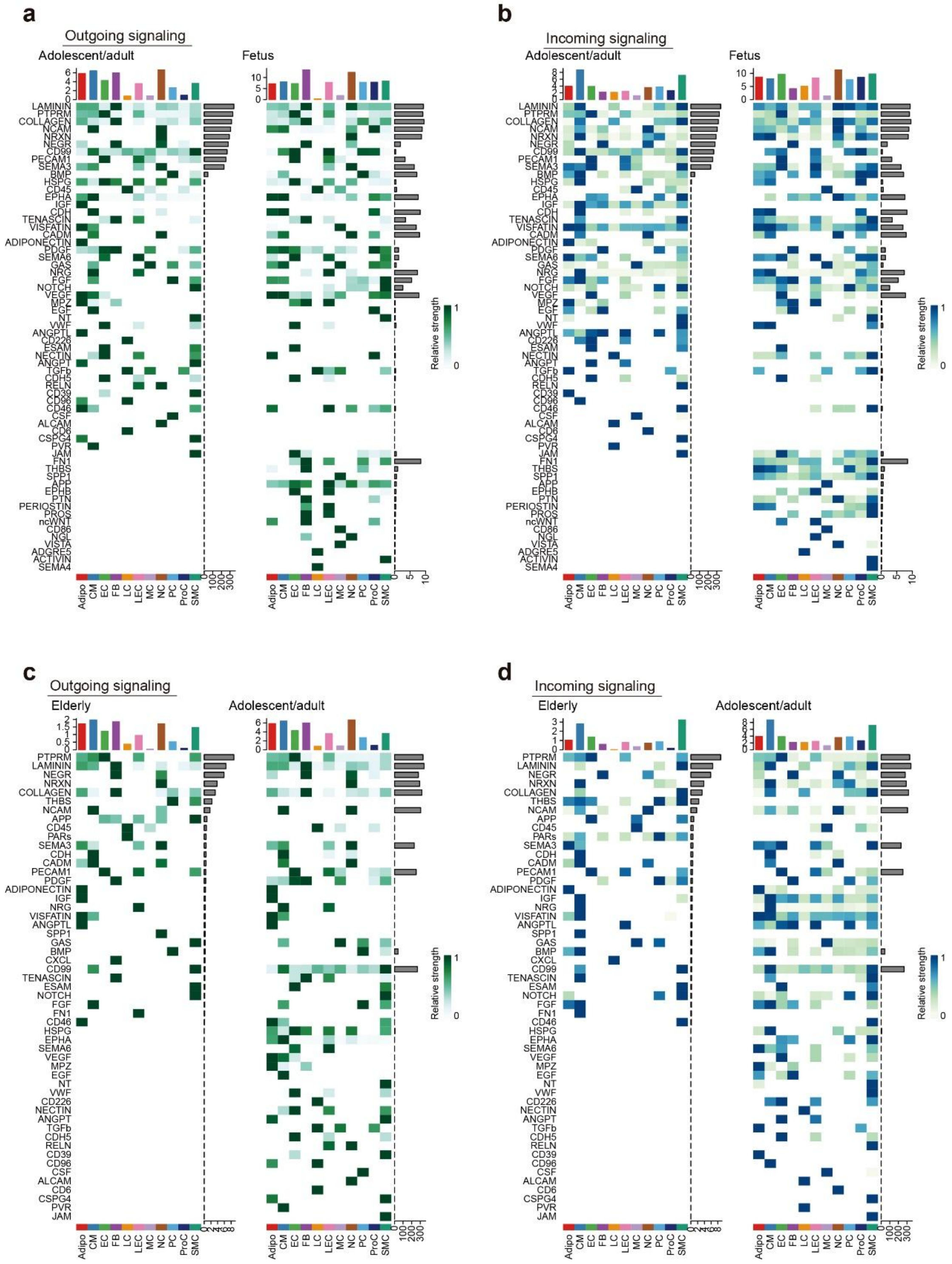

Fig. s30 | Supplementary information of signaling pathways. Heatmaps visualizing the relative signaling strength of signaling pathways across the main cell types, and values were row-scaled. The colored bar plots (top) showing the total signaling strength of a cell type by summarizing all signaling pathways displayed in the heatmaps. The grey bar plots (right) showing the total signaling strength of a signaling pathway by summarizing all cell types displayed in the heatmaps. **a.** Outgoing signaling strength comparing the adolescent/adult groups (AG4 and AG5) and fetus groups (AG1, AG2, and AG3). **b.** Incoming signaling strength comparing the adolescent/adult groups (AG4 and

AG5) and fetus groups (AG1, AG2, and AG3). **c.** Outgoing signaling strength comparing the adolescent/adult groups (AG4 and AG5) and AG6. **d.** Incoming signaling strength comparing the adolescent/adult groups (AG4 and AG5) and AG6.

Index for Tables S1 to S55

Supplementary_Table_1: detailed sample information, gender information, statistical analysis of gender
Supplementary_Table_2: gene sets of major cardiac cell types
Supplementary_Table_3: proportion of major cardiac cell types among samples
Supplementary_Table_4: proportion of CMs and non-CMs among samples with ProC excluded
Supplementary_Table_5: proportion of ProC in the major cell type level
Supplementary_Table_6: gene sets of ProC cell states
Supplementary_Table_7: proportion of ProC cell states
Supplementary_Table_8: gene sets of cardiomyocyte cell states
Supplementary_Table_9: proportion of cardiomyocyte cell states
Supplementary_Table_10: overlapping genes between CM4 (this study) and failing CMs (Liu *et al*. study)
Supplementary_Table_11: aging, SASP, dilated cardiomyopathy, heart failure, cardiac hypertrophy, and ventricular fibrillation gene sets
Supplementary_Table_12: gene sets of mfuzz analyses in CMs
Supplementary_Table_13: GO results of mfuzz gene sets in CMs
Supplementary_Table_14: differentially expressed genes of pairwise comparison in CMs
Supplementary_Table_15: GO results of differentially expressed genes of pairwise comparison in CMs
Supplementary_Table_16: gene sets and GO results of monocle 2 analyses in CMs
Supplementary_Table_17: SCENIC result of CMs
Supplementary_Table_18: mfuzz results of SCENIC gene sets in CMs
Supplementary_Table_19: FACS results of the percentage of cTnT+ cells
Supplementary_Table_20: RT-qPCR results in AC-16 cell line
Supplementary_Table_21: RT-qPCR results in hiPSC-derived CMs
Supplementary_Table_22: western blot results in AC-16 and hiPSC-derived CMs
Supplementary_Table_23: cell size results in AC-16 and hiPSC-derived CMs
Supplementary_Table_24: SA-β-Gal results in AC-16 and hiPSC-derived CMs
Supplementary_Table_25: BrdU results in hiPSC-derived CMs
Supplementary_Table_26: IL-8 results in AC-16 and hiPSC-derived CMs
Supplementary_Table_27: Seahorse results in AC-16 and hiPSC-derived CMs
Supplementary_Table_28: RNA-sequencing data of *PRDM16* knock-down CMs
Supplementary_Table_29: Lifespan snRNA-seq results of mouse heart: major cell type and CMs
Supplementary_Table_30: RT-qPCR results in the hearts of young and aging mice
Supplementary_Table_31: Echocardiography of mouse hearts
Supplementary_Table_32: Prdm16-overexpression snRNA-seq results of mouse heart
Supplementary_Table_33: gene sets of endothelial cell states
Supplementary_Table_34: proportion of endothelial cell states
Supplementary_Table_35: GO results of mfuzz gene sets in ECs
Supplementary_Table_36: differentially expressed genes and GO results of pairwise comparison in ECs

Supplementary_Table_37: gene sets and GO results of monocle 2 analyses in ECs
Supplementary_Table_38: SCENIC and mfuzz analyses of SCENIC results of ECs
Supplementary_Table_39: gene sets of fibroblast cell states
Supplementary_Table_40: proportion of fibroblast cell states
Supplementary_Table_41: GO results of mfuzz gene sets in FBs
Supplementary_Table_42: differentially expressed genes and GO results of pairwise comparison in FBs
Supplementary_Table_43: gene sets and GO results of monocle 2 analyses in FBs
Supplementary_Table_44: SCENIC and mfuzz analyses of SCENIC results of FBs
Supplementary_Table_45: gene sets of SMC cell states
Supplementary_Table_46: proportion of SMC cell states
Supplementary_Table_47: gene sets of pericyte cell states
Supplementary_Table_48: proportion of pericyte cell states
Supplementary_Table_49: gene sets of lymphoid cell states
Supplementary_Table_50: proportion of lymphoid cell states
Supplementary_Table_51: gene sets of myeloid cell states
Supplementary_Table_52: proportion of myeloid cell states
Supplementary_Table_53: cellchat reults of left ventricle
Supplementary_Table_54: cellchat reults of right ventricle
Supplementary_Table_55: key genes and GO results of prediction models

Uncropped image of fig.3G

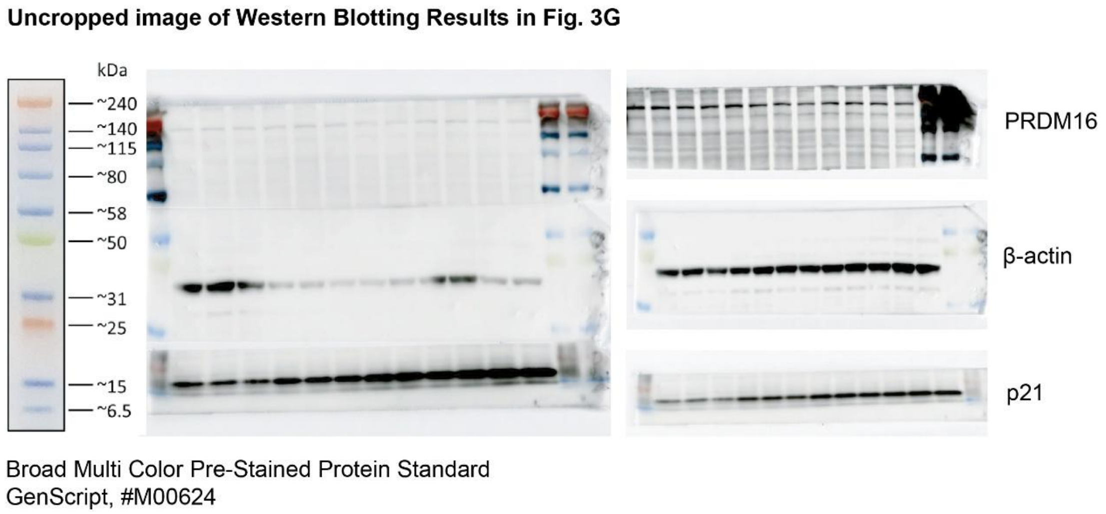

Uncropped image of fig.s13e

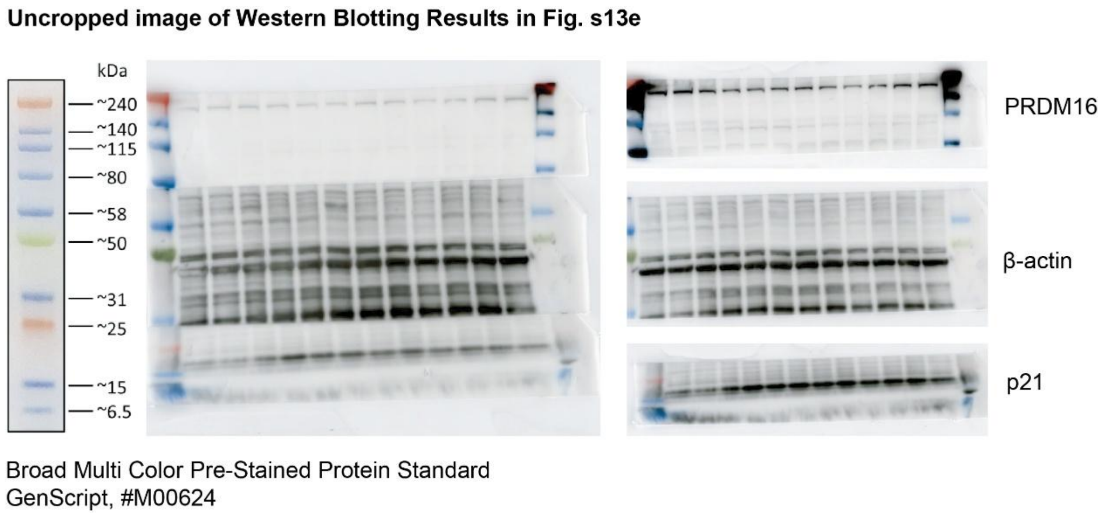

Supplement: Supplementary file 1 — Figs. S1 to S30 Legends for tables S1 to S55 Uncropped image of Fig. 3G Uncropped image of fig. S13E [file sciadv.aeg2614_sm.pdf]
